# Supplementary figures and images for: Evolutionary history of dimethylsulfoniopropionate (DMSP) demethylation enzyme DmdA in marine bacteria
Source: PeerJ. 2020 Sep 10;8:e9861. doi: 10.7717/peerj.9861 (PMC7487153; doi:10.7717/peerj.9861)

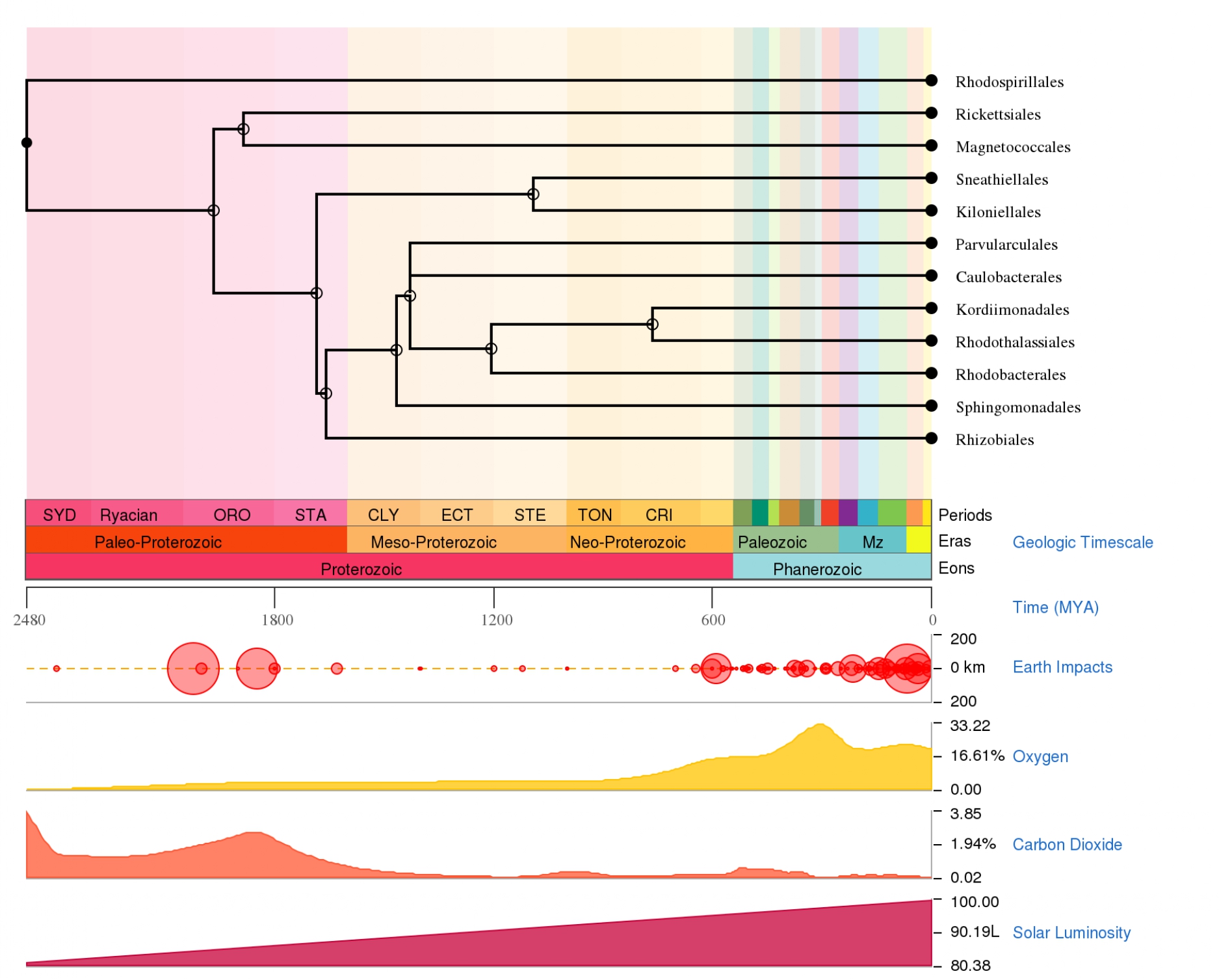

Supplement: Supplemental Information 1 — Solid circles indicate nodes that map directly to the NCBI Taxonomy and open circles nodes that were created during the polytomy resolution process as described in Hedges et al. (2015). [file peerj-08-9861-s001.jpg]

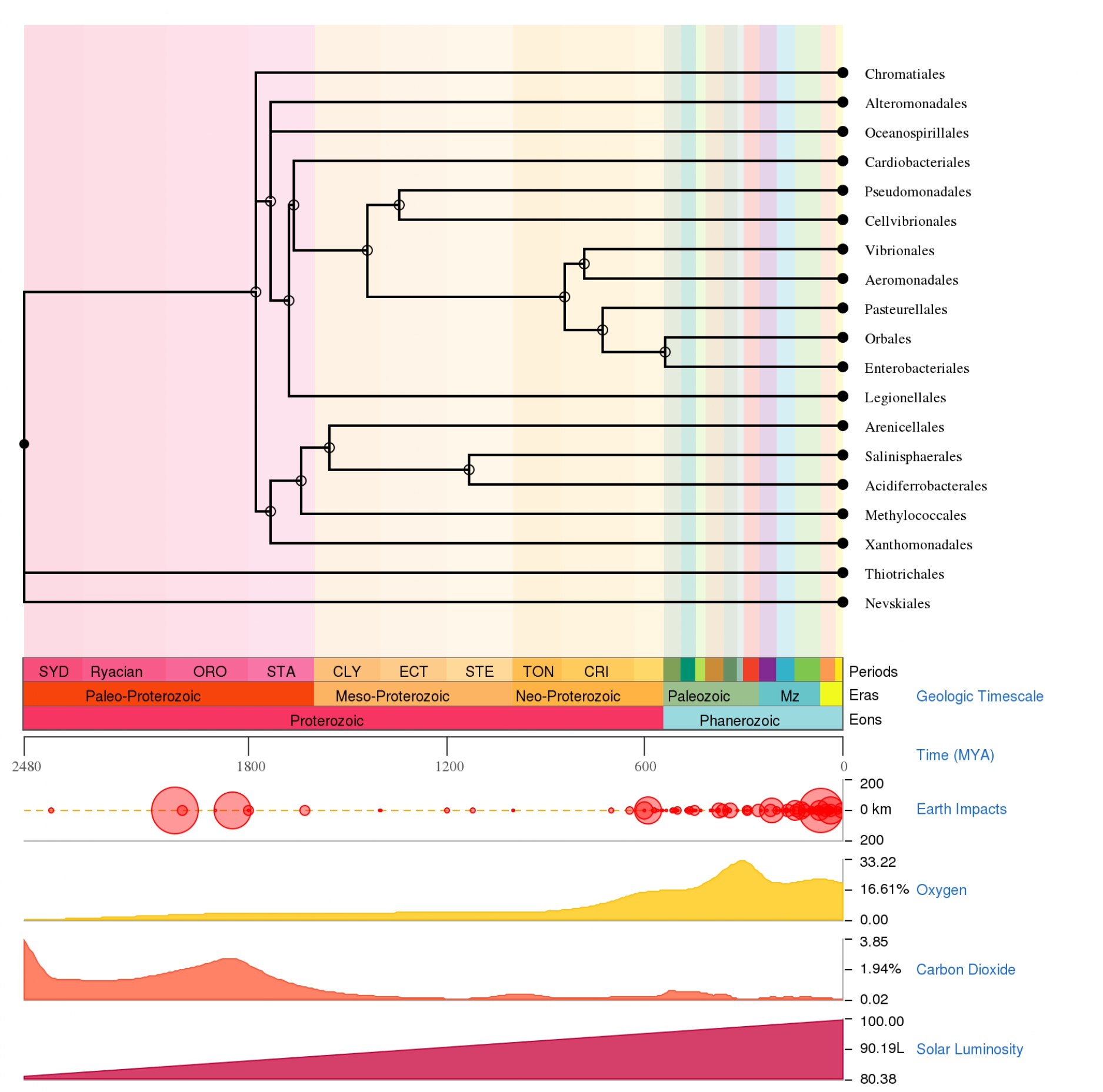

Supplement: Supplemental Information 2 — Solid circles indicate nodes that map directly to the NCBI Taxonomy and open circles nodes that were created during the polytomy resolution process as described in Hedges et al. (2015). [file peerj-08-9861-s002.jpg]

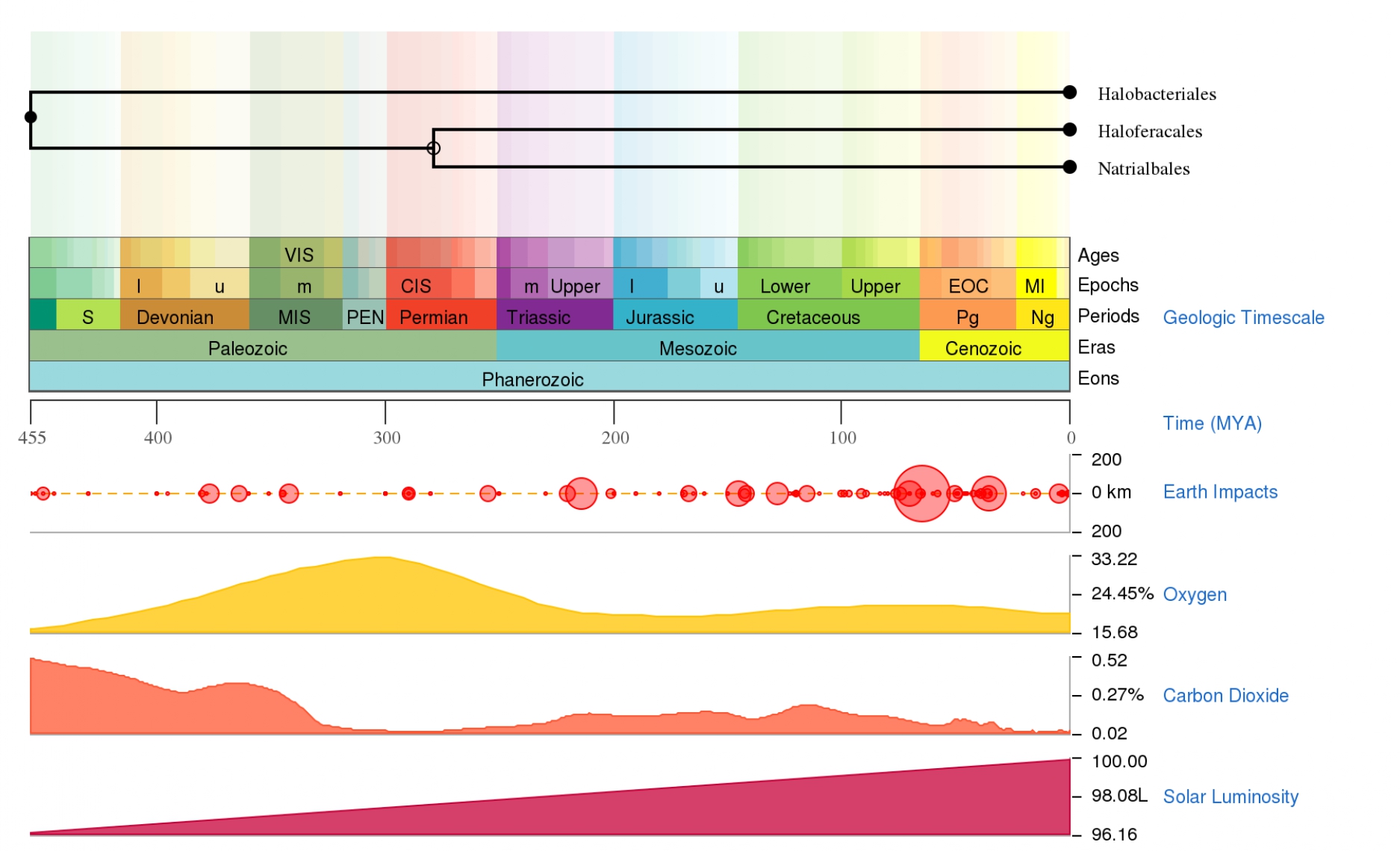

Supplement: Supplemental Information 3 — Solid circles indicate nodes that map directly to the NCBI Taxonomy and open circles nodes that were created during the polytomy resolution process as described in Hedges et al. (2015). [file peerj-08-9861-s003.jpg]

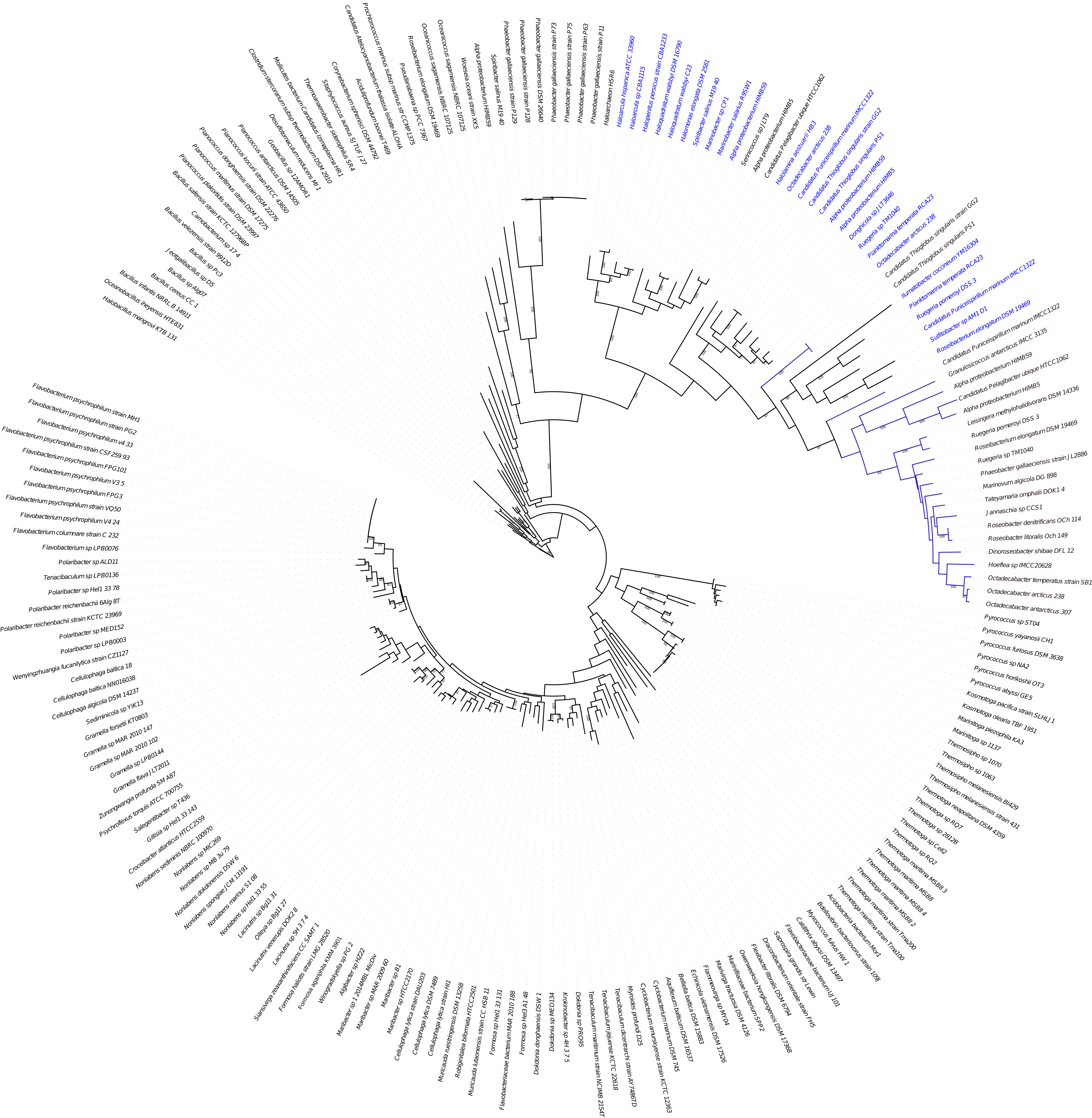

Supplement: Supplemental Information 4 — DmdA sequences are indicated with blue branches and the closer non-DmdA homologs in blue tip labels (maximum e-value of E−50). [file peerj-08-9861-s004.pdf]

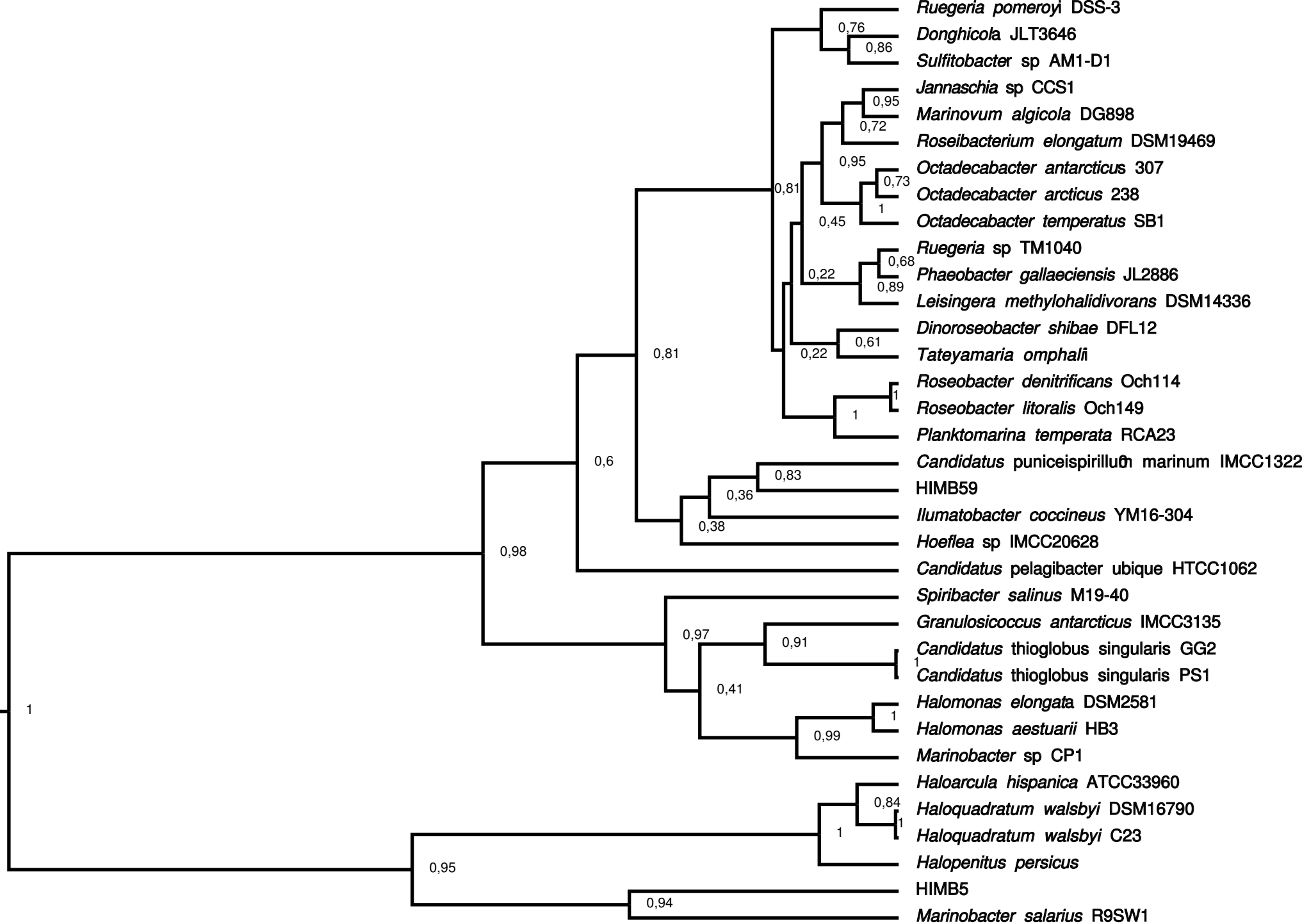

20.0

Supplement: Supplemental Information 6 [file peerj-08-9861-s006.pdf]

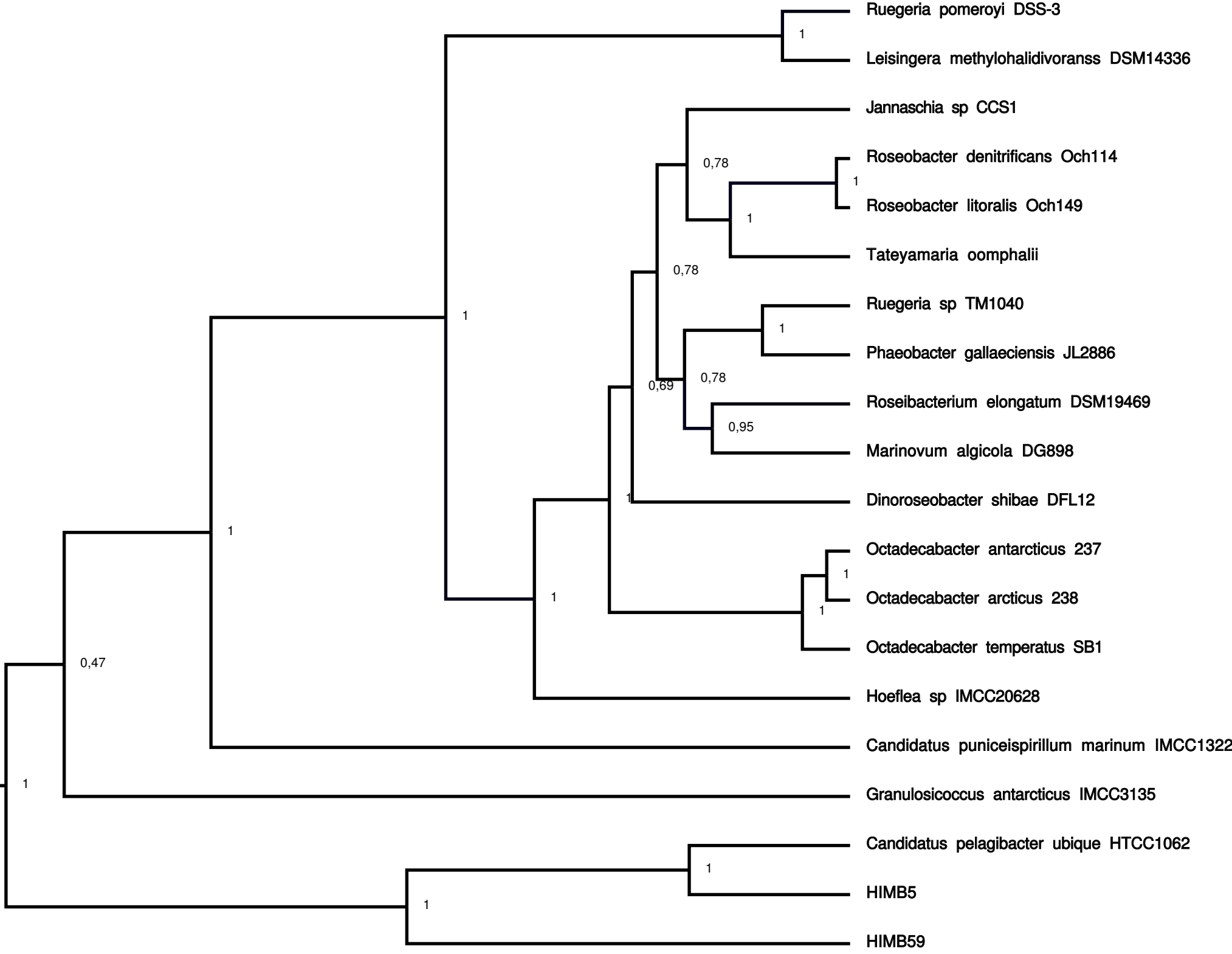

10.0

Supplement: Supplemental Information 7 — Tree was constructed by ML for topology tests and BI for an easily visualization of phylogenetic relationships in unrooted trees. [file peerj-08-9861-s007.pdf]

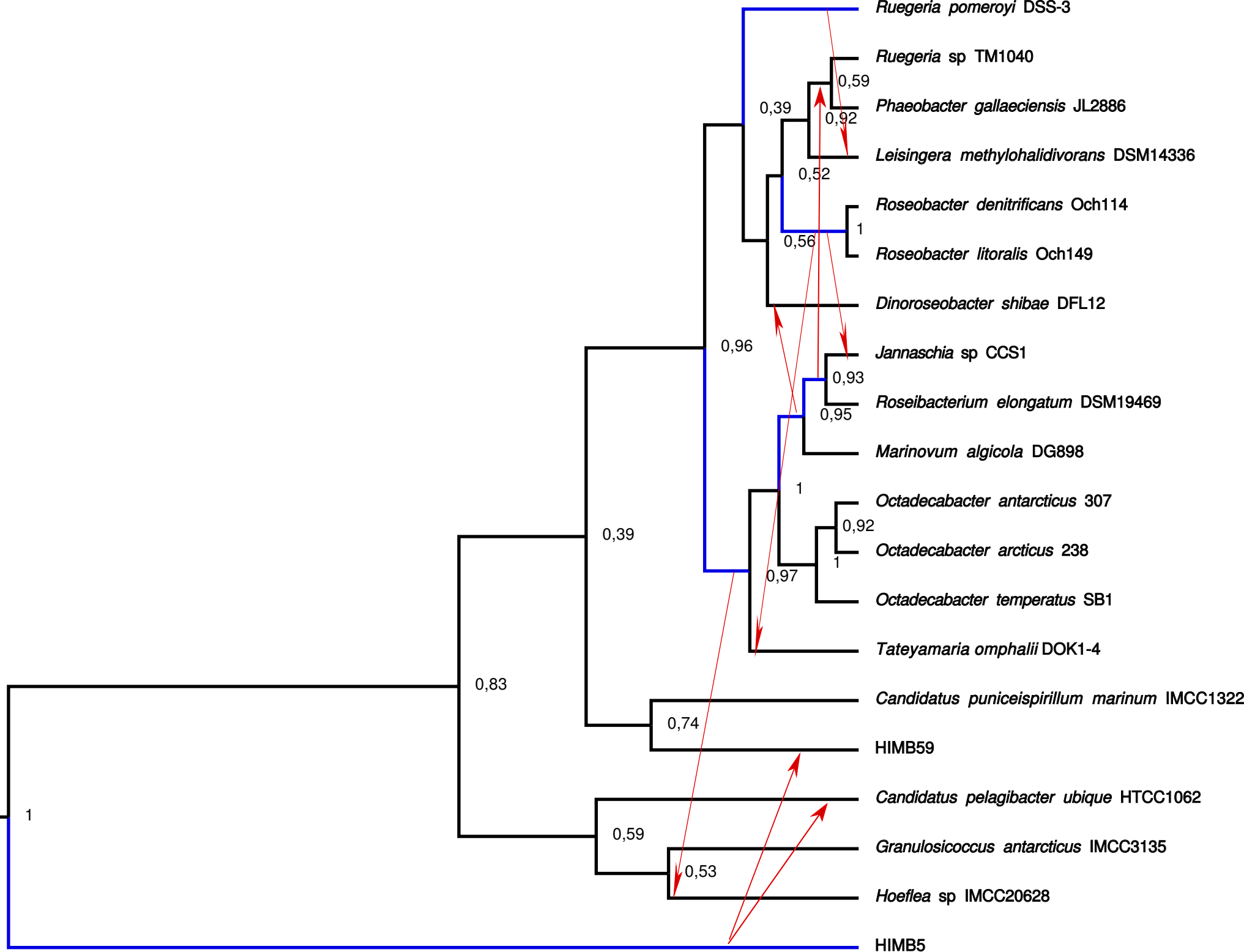

20.0

Supplement: Supplemental Information 8 — The blue branches denote HGT events and red arrows the direction of the jump which is quite small within Roseobacter or from SAR11 to Roseobacter. [file peerj-08-9861-s008.pdf]

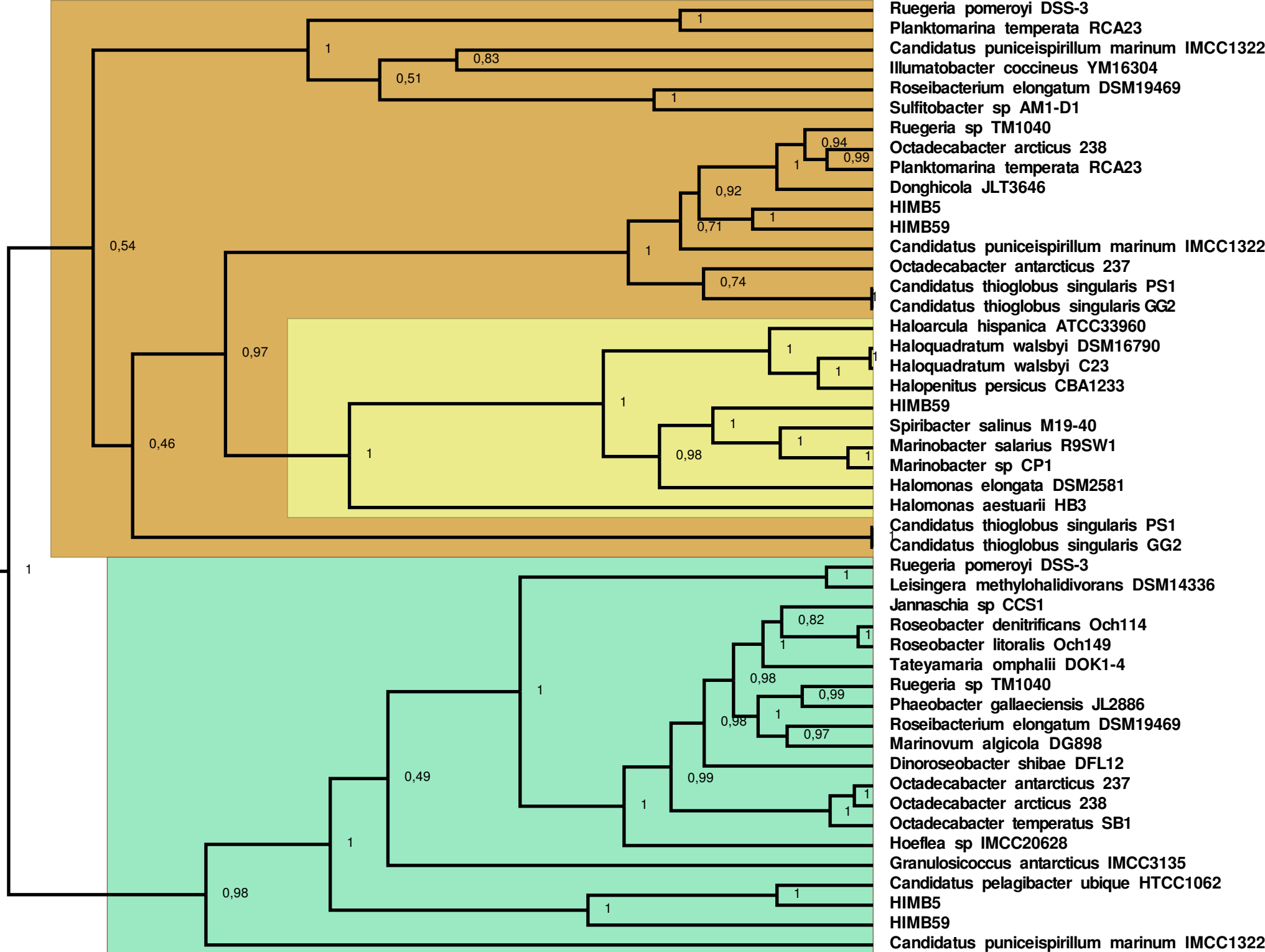

Supplement: Supplemental Information 9 — Bayesian posterior probabilities (PP) are shown to establish the support for the clades. Green color denote DmdA clade, brown color the non-DmdA clade and light yellow color the DmgdH clade. [file peerj-08-9861-s009.pdf]

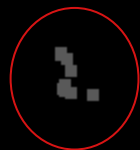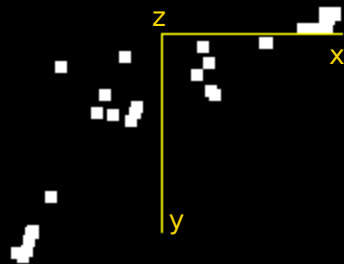

Supplement: Supplemental Information 11 — The relationships of the proteins within the alignment shown in Fig. S9b is explored using principal components analysis (PCA) and visualized based on their percent identity. The group of AEM59334.1, WP_096389816.1, CCC39909.1, CAJ51984.2, CBV41552.1, AFS48830.1, AGM40509.1, AHI3422.1, WP_053112835.1 and WP_071941841.1 (grey squares in the red circle which have DmgdH 3D structure in Table S4) are similar to each other but different from other (white squares) in the seed alignment. Each axis represents a property of the alignment common to some or all of the sequences. The most informative components to view for clustering sequences are dimensions 2, 3 and 4. Here, it shows components 2 in the axis X, 3 in the axis Y and 4 in the axis Z. The components are generated by an eigenvector decomposition of the matrix formed from the sum of substitution matrix scores at each aligned position between each pair of sequences, computed with the blosum62 matrix. [file peerj-08-9861-s011.pdf]

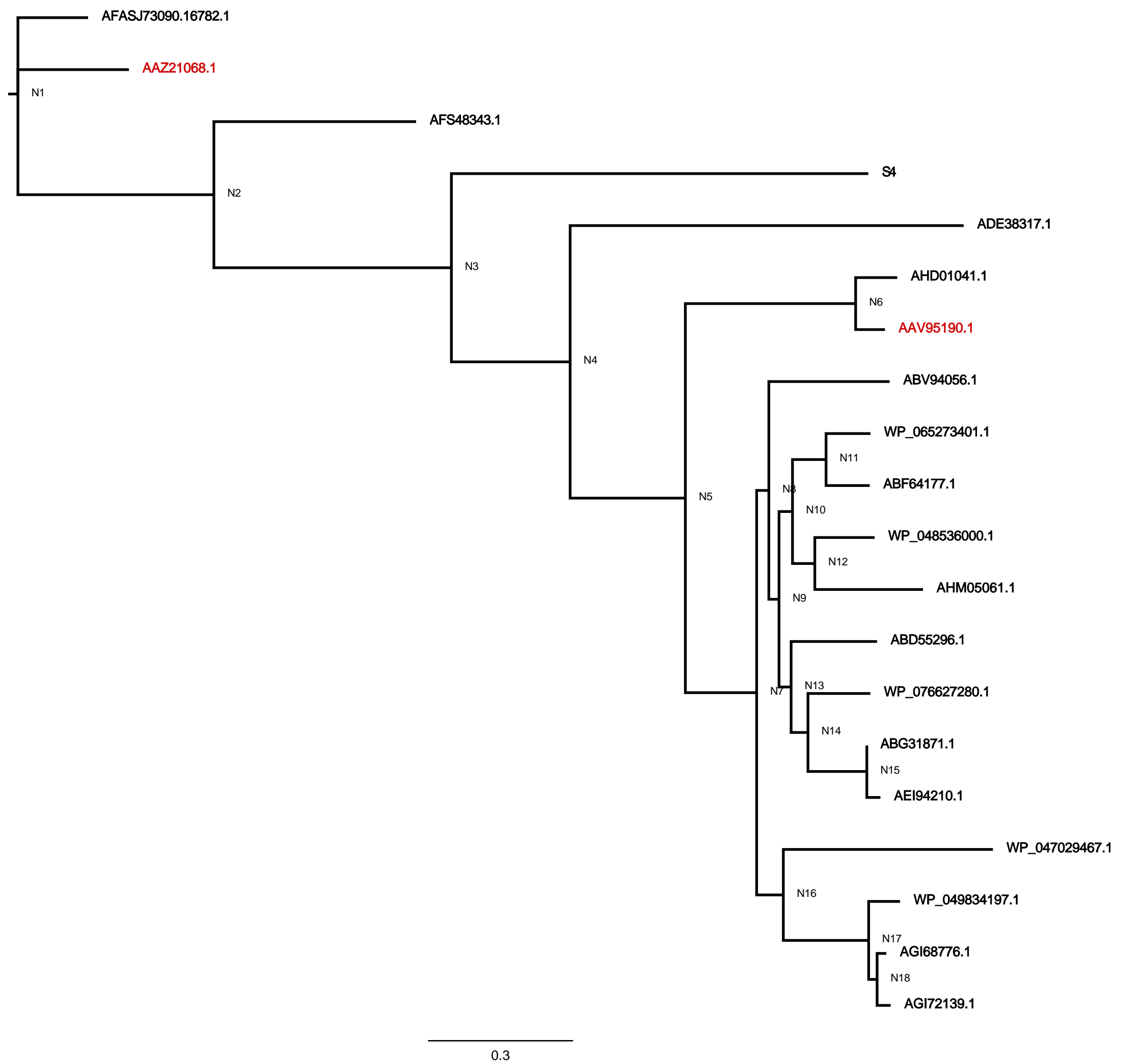

Supplement: Supplemental Information 12 — Internal nodes labels were inferred using FastML. N1is the oldest ancestor and from N2 to N18 are children. [file peerj-08-9861-s012.pdf]

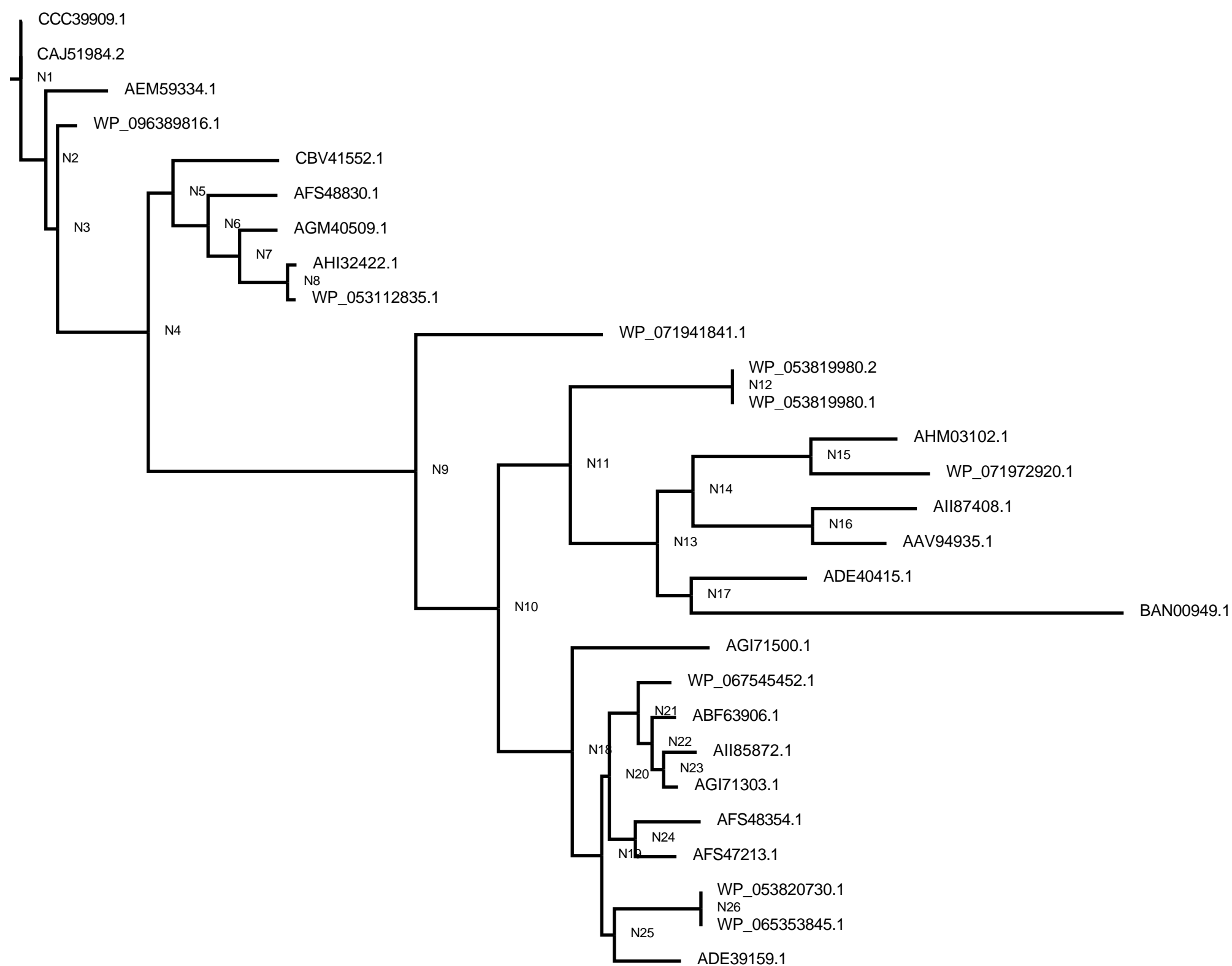

0.3

Supplement: Supplemental Information 14 — Internal nodes labels were inferred using FastML. N1 is the oldest ancestor and from N2 to N18 are children. [file peerj-08-9861-s014.pdf]

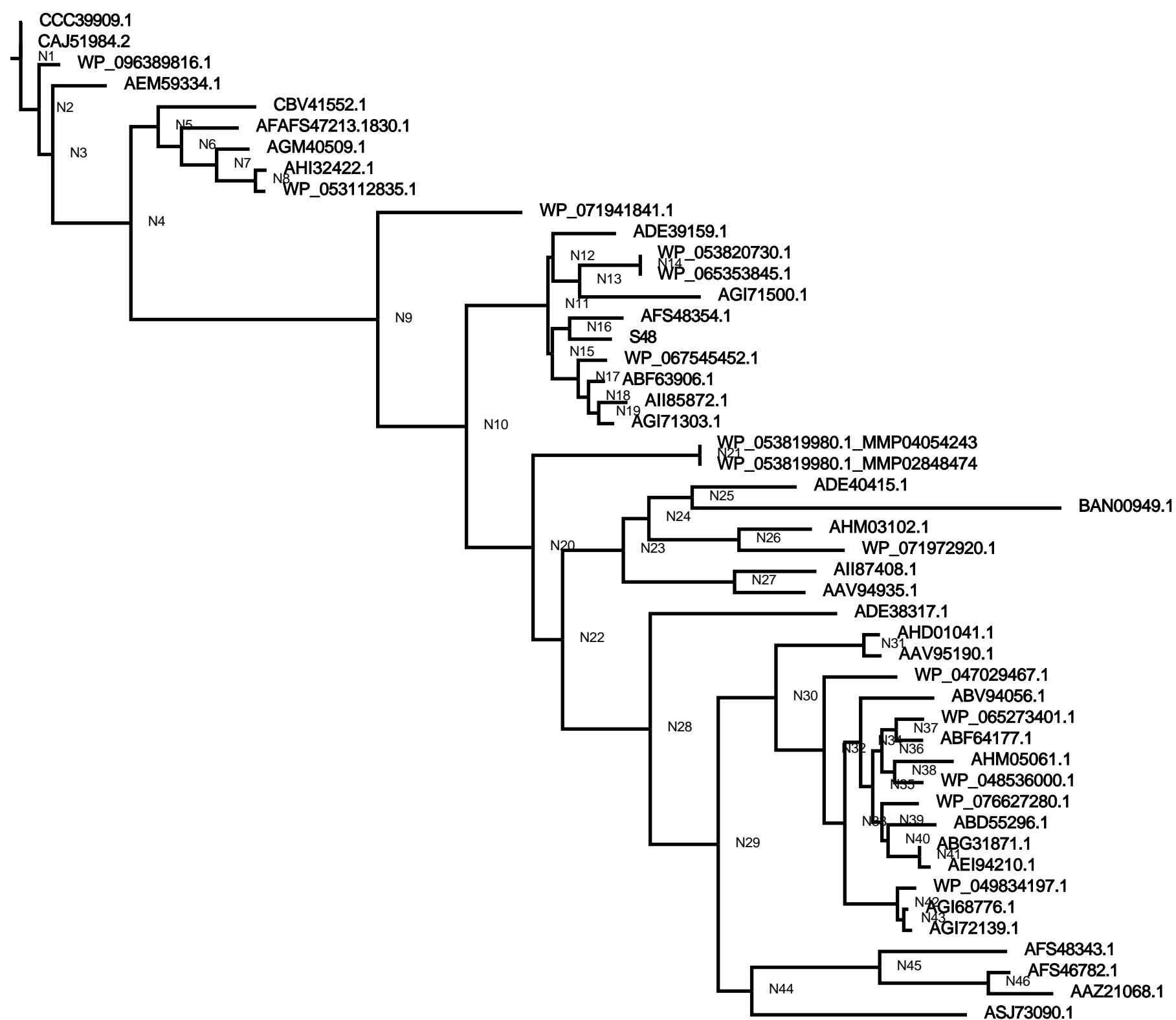

0.3

Supplement: Supplemental Information 15 — Internal nodes labels were inferred using FastML. N1 is the oldest ancestor and from N2 to N18 are children. [file peerj-08-9861-s015.pdf]

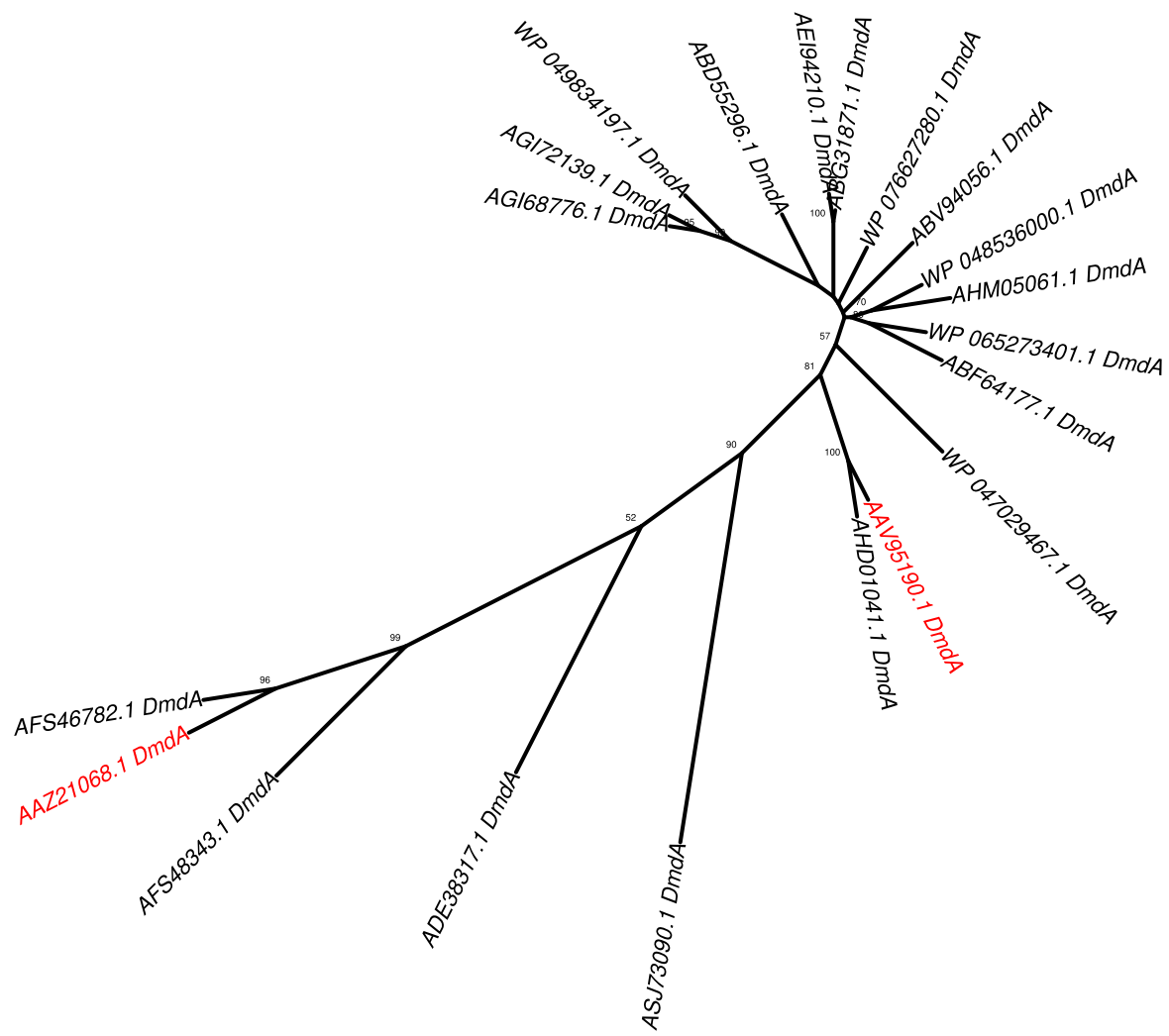

Supplement: Supplemental Information 16 — A non-parametric bootstrap is shown to establish the support for the clades. Tip labels show red color for the first dmdA gene identified (AAV95190.1: R. pomeroyi DSS-3, AAZ21068.1: Ca. P. ubique HTCC1062). [file peerj-08-9861-s016.pdf]

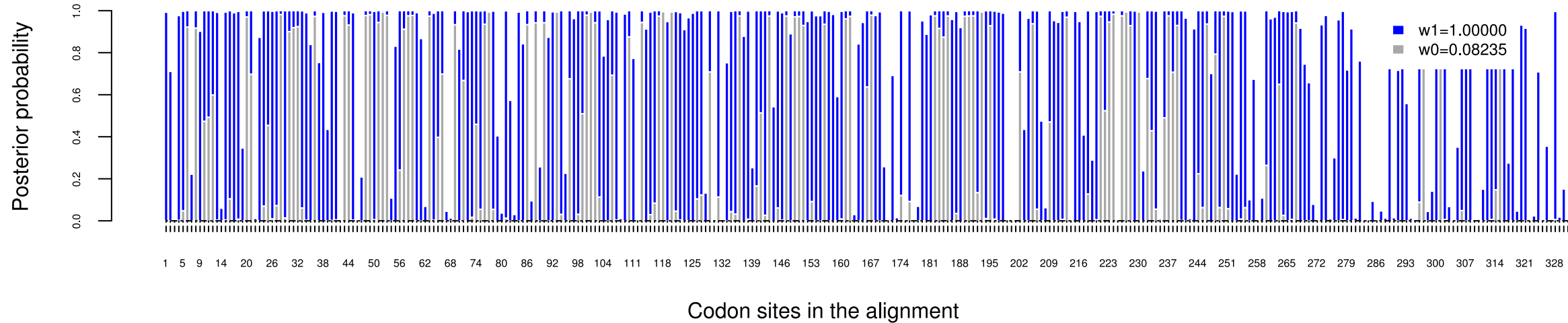

Supplement: Supplemental Information 17 — Blue bars depict the category with the dN/dS = 1 and grey bars the category with dN/dS << 1. Sites that are grey denote codons under strong purifying selection. [file peerj-08-9861-s017.pdf]

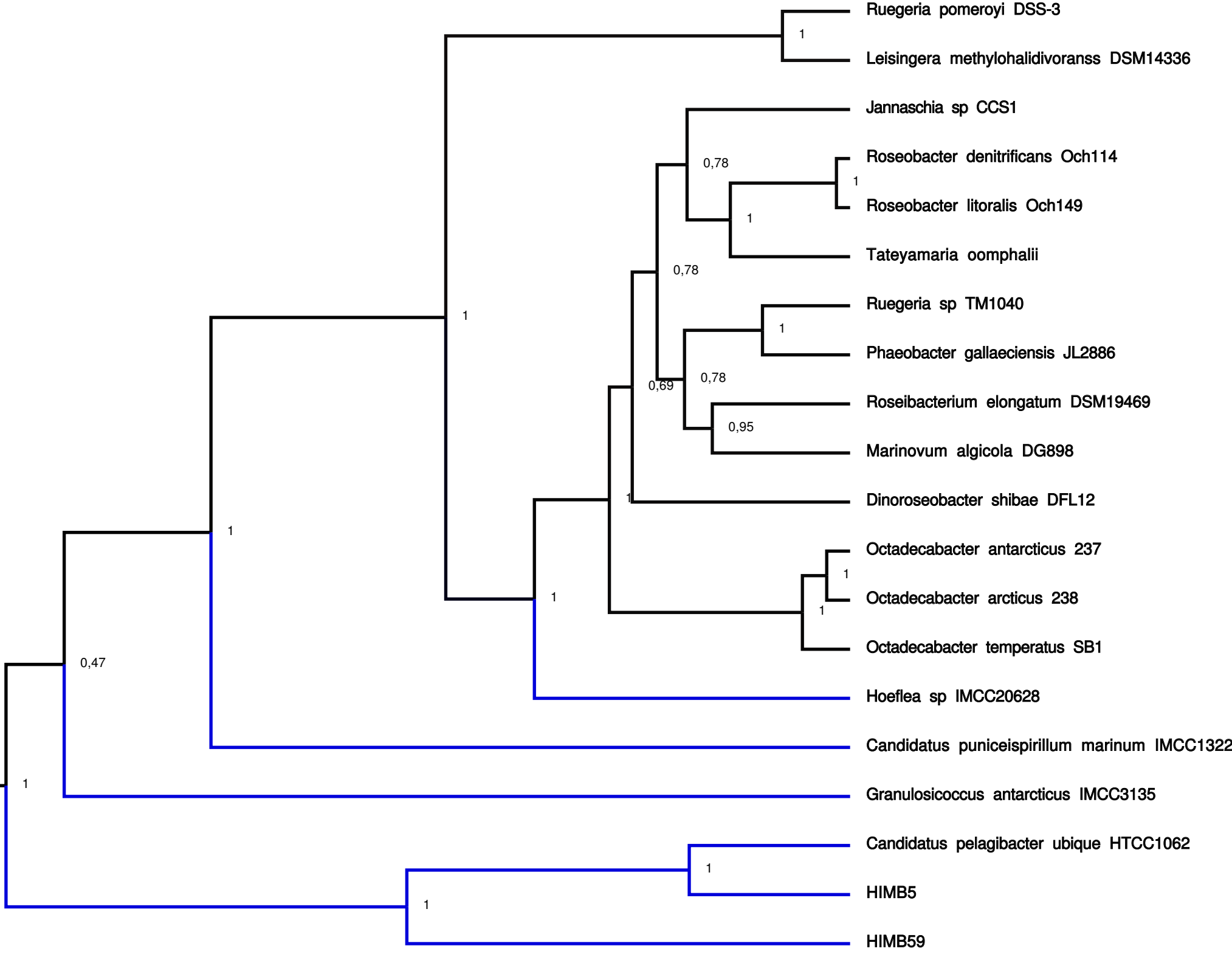

10.0

Supplement: Supplemental Information 18 — Blue color indicates the branches from group B which are compared with the rest of branches (group A: Roseobacter ) under two-ratio models. [file peerj-08-9861-s018.pdf]

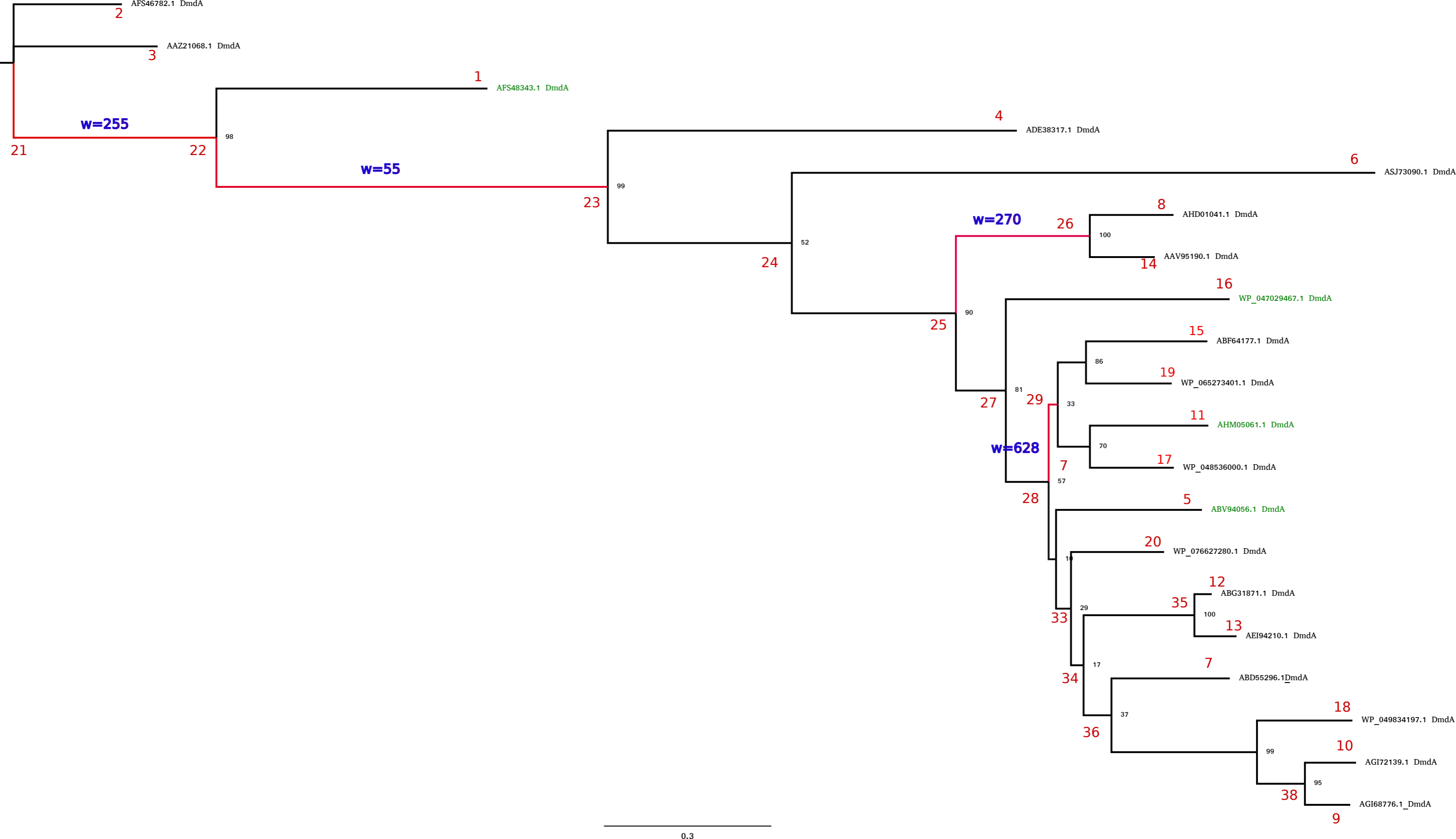

Supplement: Supplemental Information 19 — Red branches have a dN/dS value > 1. Red numbers indicate the branches. “ω” represents a dN/dS value where non-synonymous mutations are higher than synonymous mutations. Four sequences (WP_047029467, AHM05061,1, ABV94056,1, AFS48343,1) presented a significant LRT after correcting for multiple testing (green color). [file peerj-08-9861-s019.pdf]

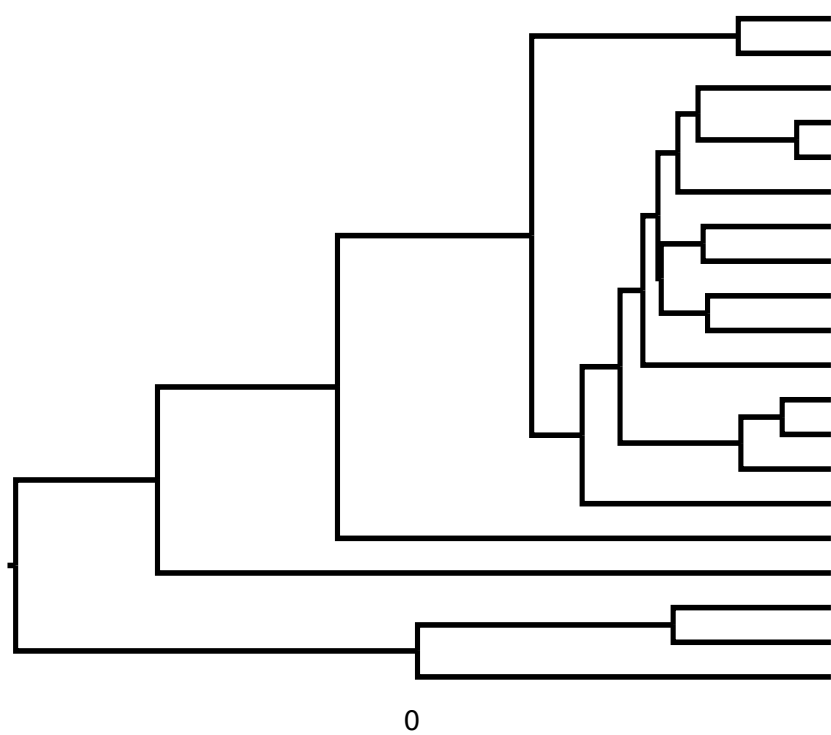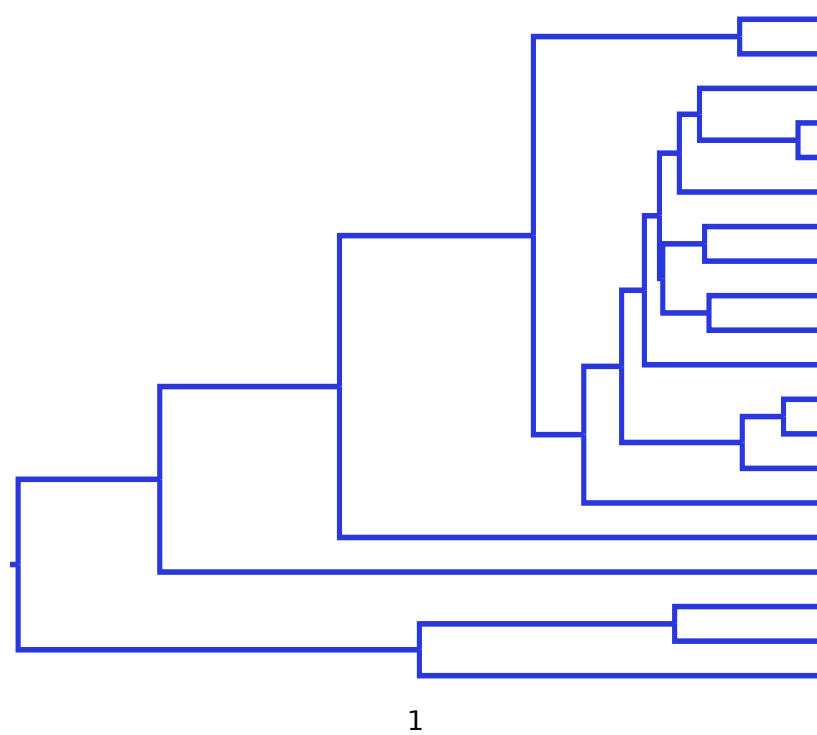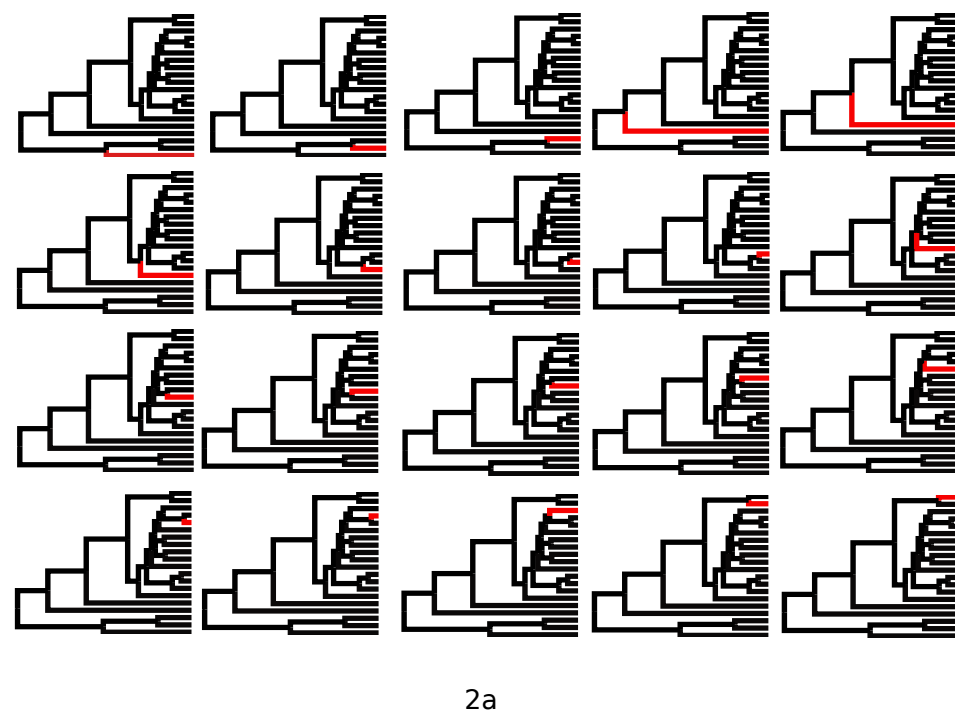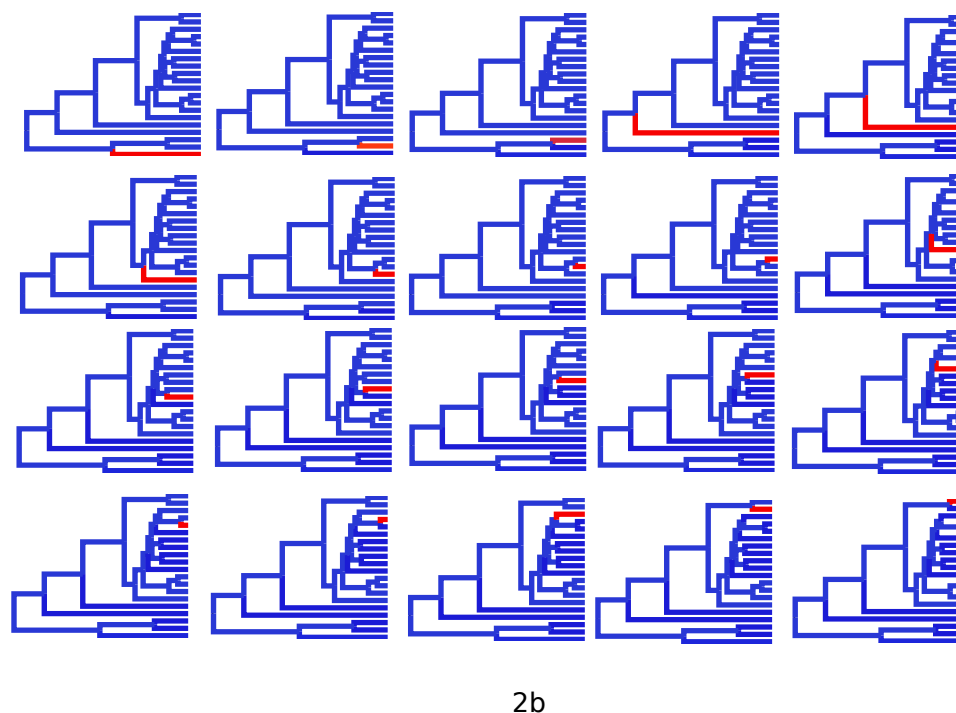

Purifying selection ○

Neutral selection ○

Positive selection ○

Supplement: Supplemental Information 20 — Red color indicates the branches of interest (foreground branches). We performed 20 tests, where only one of the branches indicated in red was considered at a time; all other branches are correspond to background-branches. [file peerj-08-9861-s020.pdf]

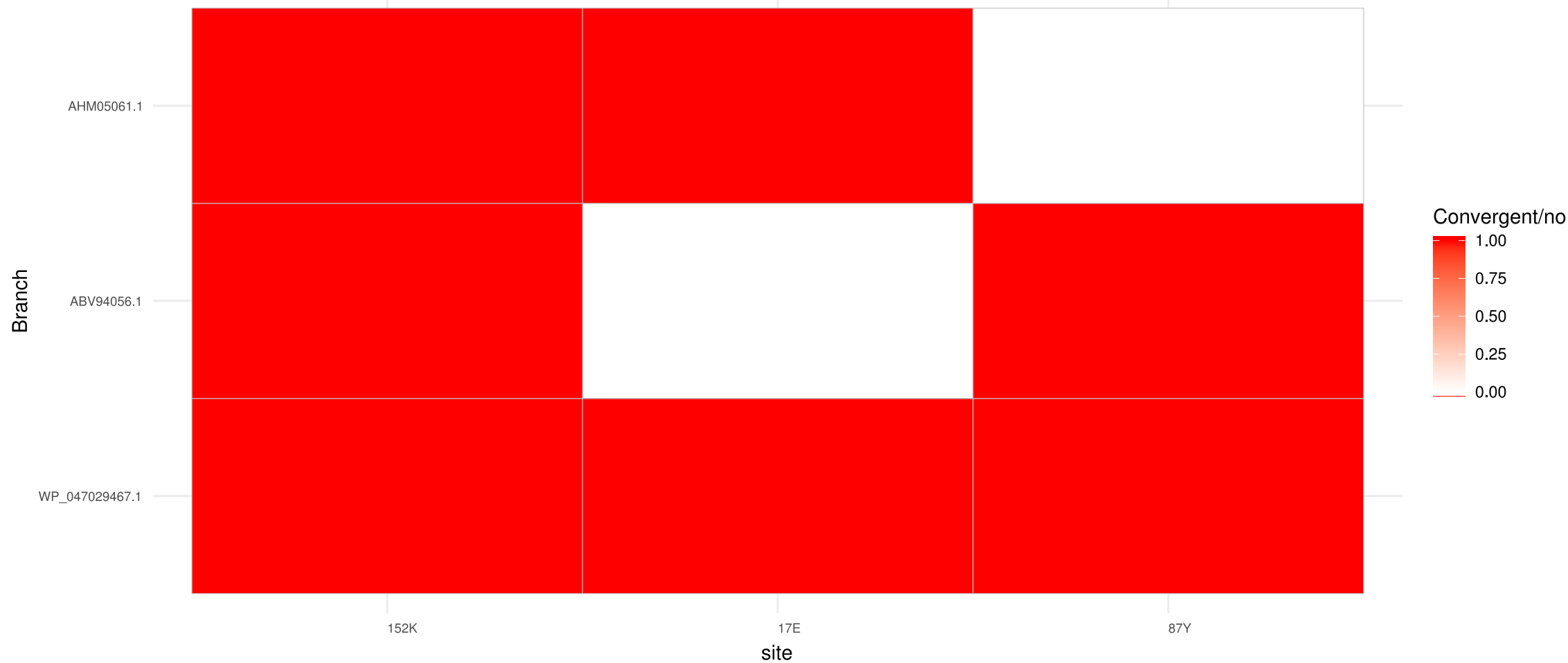

Supplement: Supplemental Information 22 — Red color identifies parallel mutational changes on specific branches of the dmdA phylogeny. The shared sites are under positive selection. Branch identifiers follow the nomenclature of Fig. S19. [file peerj-08-9861-s022.pdf]

Posterior probability

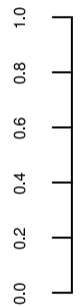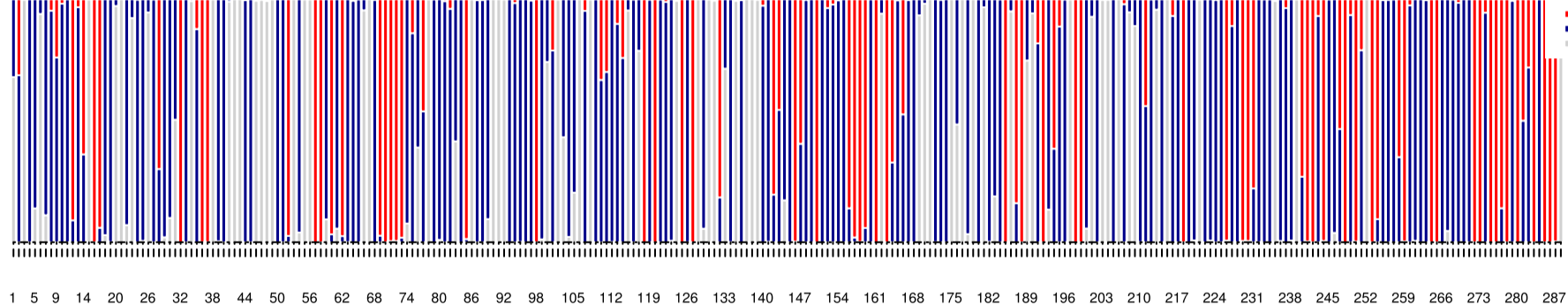

w2=0.13257  
w1=0.04569  
w0=0.00648

Codon sites in the alignment

Supplement: Supplemental Information 23 — Red and blue bars depict the categories with the highest dN/dS (values for each category are provide in the key). Sites that are mostly grey denote codons under strong purifying selection, whereas those predominantly red show codons under light purifying selection. [file peerj-08-9861-s023.pdf]

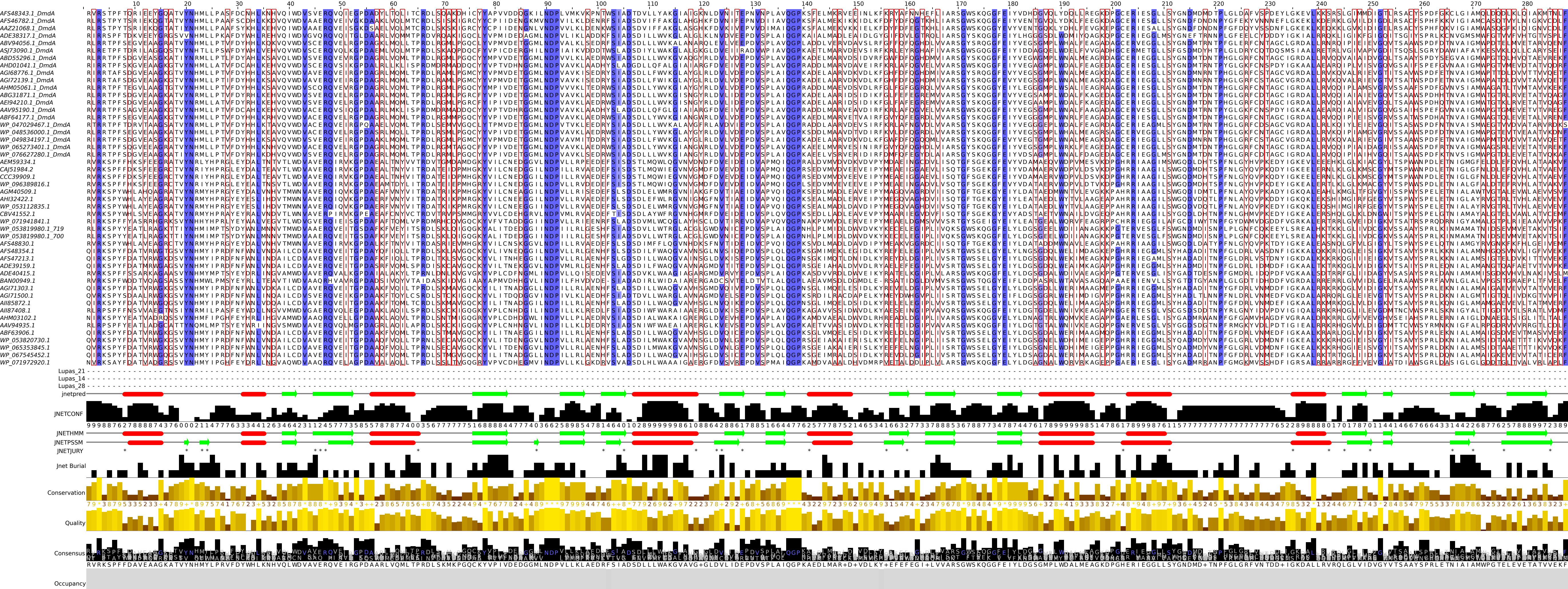

Supplement: Supplemental Information 24 — Multiple sequence alignment showing conserved regions (blue color) and codon sites evolving under divergent selective pressures (red colored columns). The secondary structure prediction using Jpred4 via Jalview is also shows for the alignment. [file peerj-08-9861-s024.pdf]

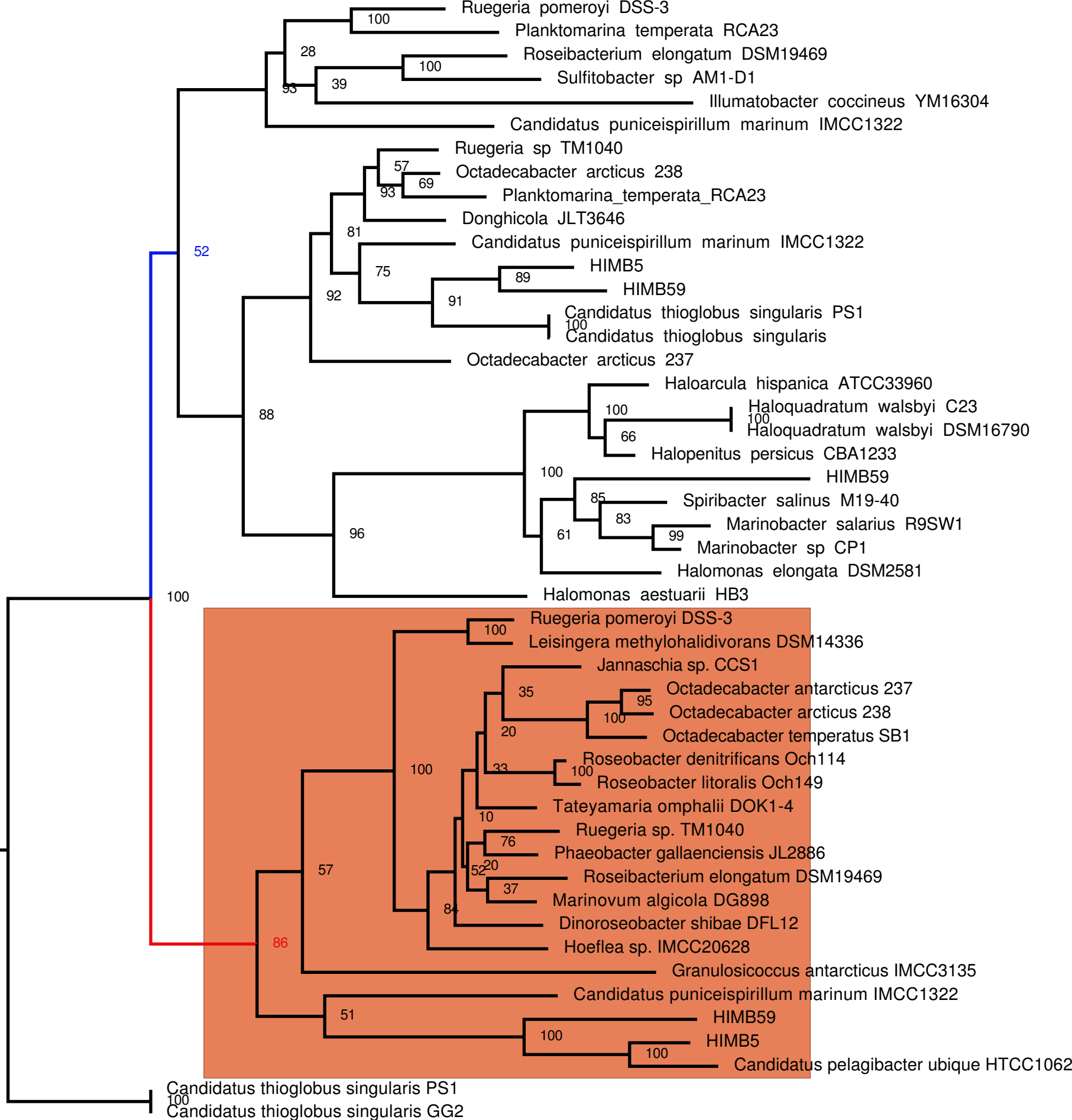

0.2

Supplement: Supplemental Information 25 — Ancestral branches to the DmdA clade and to non-DmdA clades, with red and blue colors respectively, are considered as foreground-branches in different branch-site selection models. [file peerj-08-9861-s025.pdf]
