# Supplementary material for: Evolutionary history of dimethylsulfoniopropionate (DMSP) demethylation enzyme DmdA in marine bacteria
Source: PeerJ. 2020 Sep 10;8:e9861. doi: 10.7717/peerj.9861 (PMC7487153; doi:10.7717/peerj.9861)
Supplement: Supplemental Information 10 — Blue color represents the highest level of conservation (100%) when the alignment is divided in the same four clades found in Fig. 4 and Fig. S9a (the black line indicates separation between clades). The intensity of the blue color is proportional to the percent of the residues in each column that are identical to the consensus sequence. Green color denotes ID from DmdA orthologs, brown color indicates non-DmdA clades and within it light yellow color denotes DmgdH clade. [file peerj-08-9861-s010.pdf]

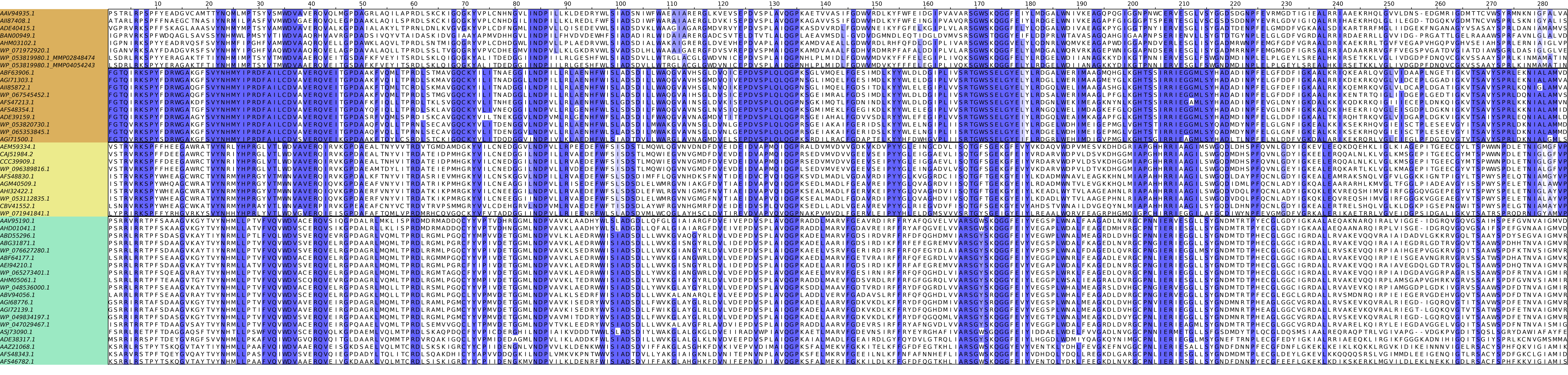
10
20
30
40
50
60
70
80
90
100
110
120
130
140
150
160
170
180
190
200
210
220
230
240
250
260
270
280
AAV94935.1
AII87408.1
ADE40415.1
BAN00949.1
AHM03102.1
WP_071972920.1
WP_053819980.1_MMP02848474
WP_053819980.1_MMP04054243
ABF63906.1
AGI71303.1
AII85872.1
WP_067545452.1
AFS47213.1
AFS48354.1
ADE39159.1
WP_053820730.1
WP_065353845.1
AGI71500.1
AEM59334.1
CAJ51984.2
CCC39909.1
WP_096389816.1
AFS48830.1
AGM40509.1
AHI32422.1
WP_053112835.1
CBV41552.1
WP_071941841.1
AAV95190.1
AHD01041.1
ABD55296.1
ABG31871.1
WP_076627280.1
ABF64177.1
AEI94210.1
WP_065273401.1
AHM05061.1
WP_048536000.1
ABV94056.1
AGI68776.1
AGI72139.1
WP_049834197.1
WP_047029467.1
ASJ73090.1
ADE38317.1
AAZ21068.1
AFS48343.1
AFS46782.1
