# Supplementary material for: Evolutionary history of dimethylsulfoniopropionate (DMSP) demethylation enzyme DmdA in marine bacteria
Source: PeerJ. 2020 Sep 10;8:e9861. doi: 10.7717/peerj.9861 (PMC7487153; doi:10.7717/peerj.9861)

PSI-BLAST (version: 2.2.26 [Sep-21-2011])
Database: uniprotkb
Sequence: (365 letters)
Length: 365

Fri, Mar 01, 2019 at 16:30:19
Fri, Mar 01, 2019 at 16:39:21

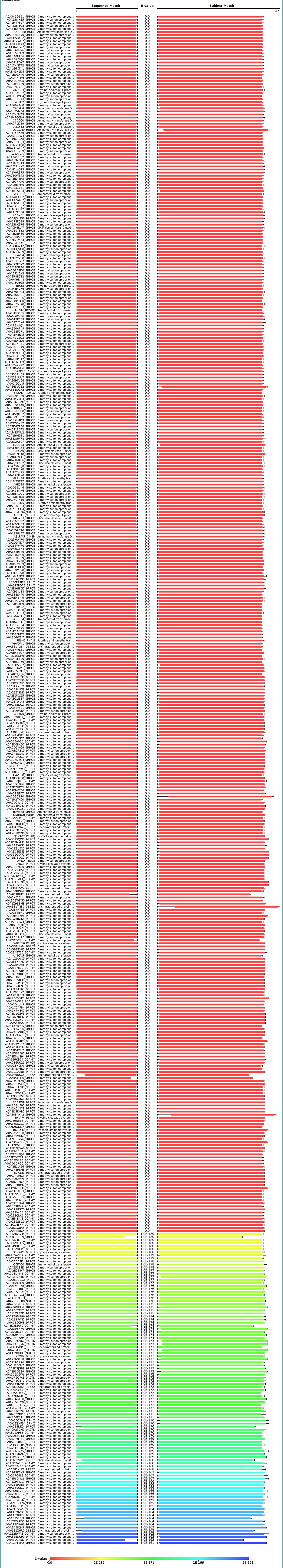

Supplement: Supplemental Information 13 [file peerj-08-9861-s013.pdf]
