# Supplementary material for: Evolutionary history of dimethylsulfoniopropionate (DMSP) demethylation enzyme DmdA in marine bacteria
Source: PeerJ. 2020 Sep 10;8:e9861. doi: 10.7717/peerj.9861 (PMC7487153; doi:10.7717/peerj.9861)
Supplement: Supplemental Information 21 — Blue color represent sites with the highest level of conservation (100%). Red squares represent sites under positive selection. The posterior probability of each site was calculated by BEB. Green asterisks indicate residues that have a conserved interaction with THF (Schuller et al., 2012). [file peerj-08-9861-s021.pdf]

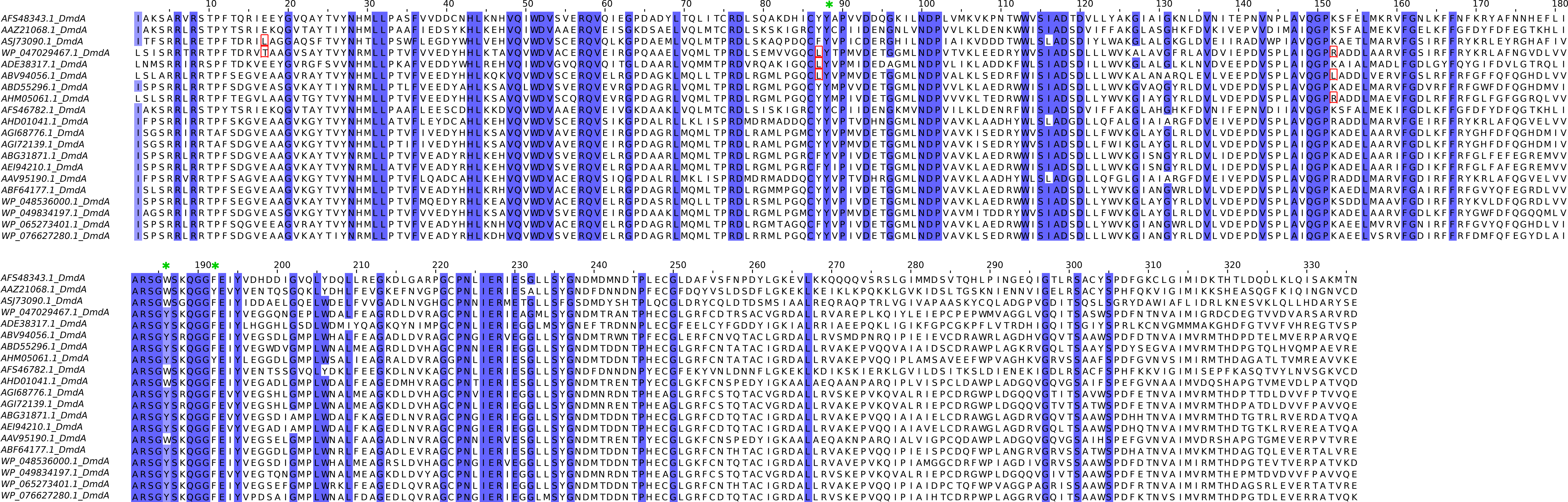
AFS48343.1_DmdA
AAZ21068.1_DmdA
ASJ73090.1_DmdA
WP_047029467.1_DmdA
ADE38317.1_DmdA
ABV94056.1_DmdA
ABD55296.1_DmdA
AHM05061.1_DmdA
AFS46782.1_DmdA
AHD01041.1_DmdA
AGI68776.1_DmdA
AGI72139.1_DmdA
ABG31871.1_DmdA
AEI94210.1_DmdA
AAV95190.1_DmdA
ABF64177.1_DmdA
WP_048536000.1_DmdA
WP_049834197.1_DmdA
WP_065273401.1_DmdA
WP_076627280.1_DmdA
10
20
30
40
50
60
70
80
90
100
110
120
130
140
150
160
170
180
190
200
210
220
230
240
250
260
270
280
290
300
310
320
330
