# Supplementary material for: Evolutionary history of dimethylsulfoniopropionate (DMSP) demethylation enzyme DmdA in marine bacteria
Source: PeerJ. 2020 Sep 10;8:e9861. doi: 10.7717/peerj.9861 (PMC7487153; doi:10.7717/peerj.9861)
Supplement: Supplemental Information 5 — DmdA sequences are indicated with blue and the first DmdA proteins identified in read color (AAV95190.1: Ruegeria pomeroyi DSS-3, AAZ21068.1: Ca. P. ubique HTCC1062). Tip labels include a maximum e-value of E−80. [file peerj-08-9861-s005.pdf]

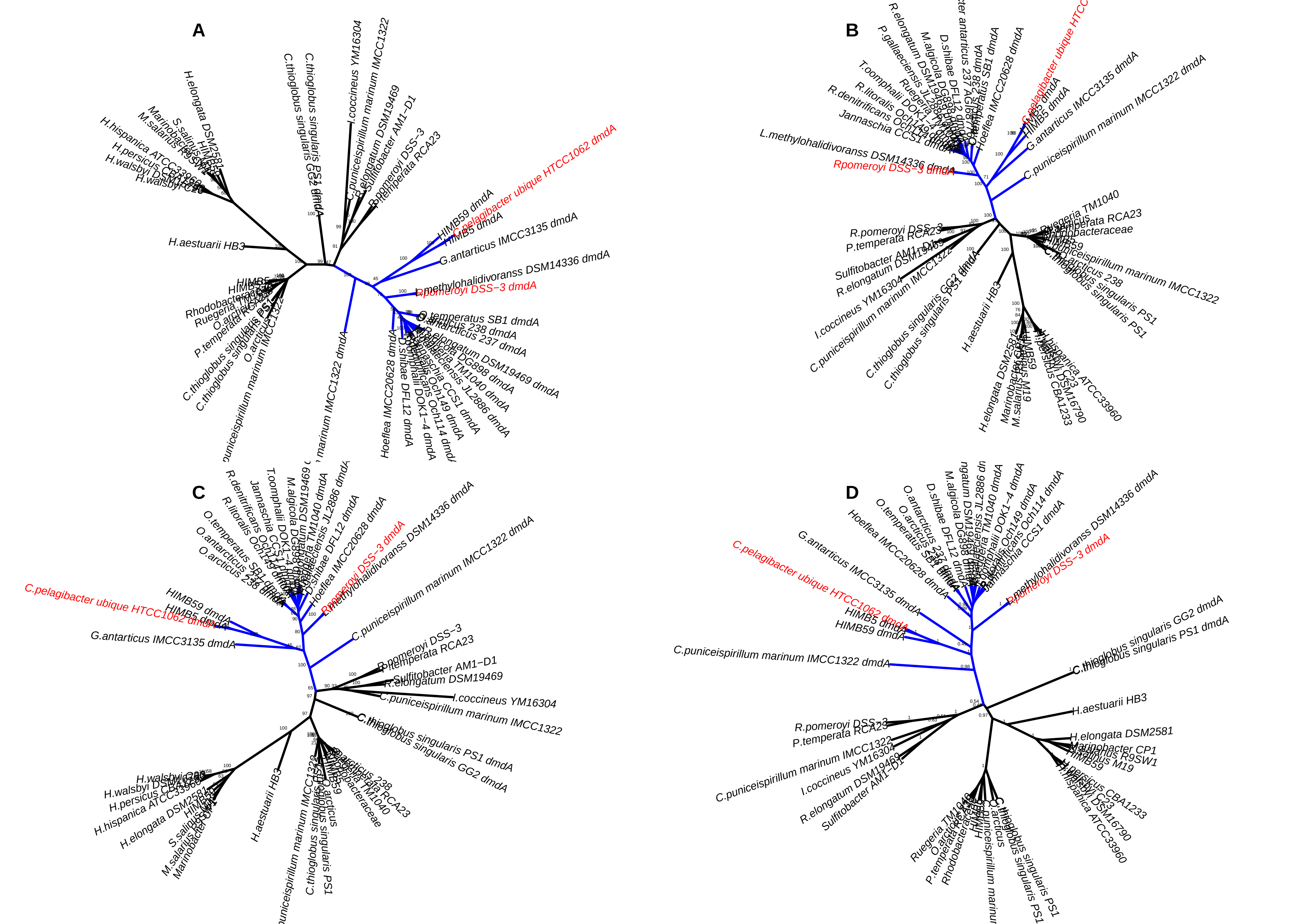

A
B
C
D
C.pelagibacter ubique HTCC1062 dmdA
Rpomeroyi DSS−3 dmdA
G.antarcticus IMCC3135 dmdA
L.methylohalidivoranss DSM14336 dmdA
HIMB59 dmdA
HIMB5 dmdA
C.puniceispirillum marinum IMCC1322 dmdA
C.puniceispirillum marinum IMCC1322
H.aestuarii HB3
I.coccineus YM16304
C.thioglobus singularis PS1 dmdA
C.thioglobus singularis GG2 dmdA
C.thioglobus singularis PS1
Hoeflea IMCC20628 dmdA
D.shibae DFL12 dmdA
O.temperatus SB1 dmdA
O.arcticus 238 dmdA
O.antarcticus 237 dmdA
R.elongatum DSM19469 dmdA
M.algicola DG898 dmdA
Ruegeria TM1040 dmdA
P.gallaeciensis JL2886 dmdA
T.oomphalii DOK1−4 dmdA
R.litoralis Och149 dmdA
R.denitrificans Och114 dmdA
Jannaschia CCS1 dmdA
R.pomeroyi DSS−3
P.temperata RCA23
Sulfitobacter AM1−D1
R.elongatum DSM19469
H.elongata DSM2581
H.hispanica ATCC33960
H.persicus CBA1233
H.walsbyi C23
H.walsbyi DSM16790
Marinobacter CP1
M.salarius R9SW1
S.salinus M19
HIMB59
Rhodobacteraceae
Ruegeria TM1040
O.arcticus
P.temperata RCA23
