# Supplementary material for: Evolutionary history of dimethylsulfoniopropionate (DMSP) demethylation enzyme DmdA in marine bacteria
Source: PeerJ. 2020 Sep 10;8:e9861. doi: 10.7717/peerj.9861 (PMC7487153; doi:10.7717/peerj.9861)
Supplement: Supplementary file 6 [file peerj-08-9861-s027.odt]

|  | Split Distance MM | Split Distance random |
| --- | --- | --- |
| Mean | SD | Mean | SD |
| BEAST2 vs BEAST2 | 0 | 0 | 0.9988 | 0.002 |
| BEAST2 vs RAxML | 0.1990 | 0 | 0.9988 | 0.002 |
