# Supplementary material for: Evolutionary history of dimethylsulfoniopropionate (DMSP) demethylation enzyme DmdA in marine bacteria
Source: PeerJ. 2020 Sep 10;8:e9861. doi: 10.7717/peerj.9861 (PMC7487153; doi:10.7717/peerj.9861)
Supplement: Supplementary file 7 [file peerj-08-9861-s028.odt]

|  | Split Distance MM | Split Distance random |
| --- | --- | --- |
| Mean | SD | Mean | SD |
| BEAST2 vs BEAST2 | 0.6178 | 0.108 | 0.9876 | 0.014 |
| BEAST2 vs Phylobayes | 0.6118 | 0.108 | 0.9870 | 0.016 |
| BEAST2 vs Phylip | 0.6077 | 0.099 | 0.9880 | 0.012 |
| BEAST2 vs RAxML | 0.6123 | 0.103 | 0.9880 | 0.012 |
| Phylip vs Phylobayes | 0.5891 | 0.115 | 0.9874 | 0.014 |
| Phylip vs RAxML | 0.6018 | 0.113 | 0.9880 | 0.013 |
| Phylobayes vs RAxML | 0.5923 | 0.112 | 0.9870 | 0.017 |
