# Supplementary material for: Evolutionary history of dimethylsulfoniopropionate (DMSP) demethylation enzyme DmdA in marine bacteria
Source: PeerJ. 2020 Sep 10;8:e9861. doi: 10.7717/peerj.9861 (PMC7487153; doi:10.7717/peerj.9861)
Supplement: Supplementary file 8 [file peerj-08-9861-s029.odt]

| Group | pKH* | pSH* | pAU* |
| --- | --- | --- | --- |
| DmdA family | 0.0010 | 0.0010 | 0.0001 |
