# Supplementary material for: Evolutionary history of dimethylsulfoniopropionate (DMSP) demethylation enzyme DmdA in marine bacteria
Source: PeerJ. 2020 Sep 10;8:e9861. doi: 10.7717/peerj.9861 (PMC7487153; doi:10.7717/peerj.9861)
Supplement: Supplementary file 9 [file peerj-08-9861-s030.odt]

| Sequence information | Predicted model | Best structural analog from PDB |
| --- | --- | --- |
| Gene name | ID | C-score1 | TM-score2 ± dev | Gene name | Organism | PDB ID3 | TM-score |
| dmdA4 | AAV95190.1 | 1.45 | 0.92 ± 0.06 | dmdA | Ca. P. ubique HTCC1062 | 3tfhA | 0.974 |
| dmdA | AHD01041.1 | 1.69 | 0.95 ± 0.05 | dmdA | Ca. P. ubique HTCC1062 | 3tfhA | 0.990 |
| dmdA | WP_047029467.1 | 2 | 0.99 ± 0.04 | dmdA | Ca. P. ubique HTCC1062 | 3tfhA | 0.997 |
| dmdA | WP_048536000.1 | 2 | 0.99 ± 0.04 | dmdA | Ca. P. ubique HTCC1062 | 3tfhA | 0.997 |
| dmdA | AHM05061.1 | 2 | 0.99 ± 0.04 | dmdA | Ca. P. ubique HTCC1062 | 3tfhA | 0.989 |
| dmdA | ABF64177.1 | 1.62 | 0.94 ± 0.05 | dmdA | Ca. P. ubique HTCC1062 | 3tfiA | 0.947 |
| dmdA | WP_065273401.1 | 2 | 0.99 ± 0.04 | dmdA | Ca. P. ubique HTCC1062 | 3tfhA | 0.997 |
| dmdA | WP_076627280.1 | 2 | 0.99 ± 0.04 | dmdA | Ca. P. ubique HTCC1062 | 3tfhA | 0.997 |
| dmdA | AEI94210.1 | 2 | 0.99 ± 0.04 | dmdA | Ca. P. ubique HTCC1062 | 3tfhA | 0.997 |
| dmdA | ABG31871.1 | 2 | 0.99 ± 0.04 | dmdA | Ca. P. ubique HTCC1062 | 3tfhA | 0.997 |
| dmdA | ABD55296.1 | 2 | 0.99 ± 0.04 | dmdA | Ca. P. ubique HTCC1062 | 3tfhA | 0.997 |
| dmdA | WP_049834197.1 | 2 | 0.99 ± 0.04 | dmdA | Ca. P. ubique HTCC1062 | 3tfhA | 0.997 |
| dmdA | AGI72139.1 | 2 | 0.99 ± 0.04 | dmdA | Ca. P. ubique HTCC1062 | 3tfhA | 0.997 |
| dmdA | ABV94056.1 | 2 | 0.99 ± 0.04 | dmdA | Ca. P. ubique HTCC1062 | 3tfhA | 0.998 |
| dmdA | AAZ21068.1 | 2 | 0.99 ± 0.04 | dmdA | Ca. P. ubique HTCC1062 | 3tfhA | 0.997 |
| dmdA | AFS46782.1 | 1.95 | 0.99 ± 0.04 | dmdA | Ca. P. ubique HTCC1062 | 3tfhA | 0.997 |
| dmdA | AFS48343.1 | 2 | 0.99 ± 0.04 | dmdA | Ca. P. ubique HTCC1062 | 3tfhA | 0.995 |
| dmdA | AGI68776.1 | 2 | 0.99 ± 0.04 | dmdA | Ca. P. ubique HTCC1062 | 3tfhA | 0.997 |
| dmdA | ASJ73090.1 | 1.77 | 0.96 ± 0.05 | dmdA | Ca. P. ubique HTCC1062 | 3tfhA | 0.956 |
| dmdA | ADE38317.1 | 1.96 | 0.99 ± 0.04 | dmdA | Ca. P. ubique HTCC1062 | 3tfhA | 0.992 |
| gcvT5 | AEM59334.1 | 2.53 | 0.42 ± 0.14 | dmgdh6 | Rattus norvegicus | 4p9sA | 0.637 |
| gcvT | WP_096389816.1 | 0.48 | 0.78 ± 0.10 | dmgdh | Rattus norvegicus | 4p9sA | 0.885 |
| gcvT | CAJ51984.2 | 0.23 | 0.68 ± 0.12 | dmgdh | Rattus norvegicus | 4p9sA | 0.855 |
| gcvT | CCC39909.1 | -0.06 | 0.71 ± 0.12 | dmgdh | Rattus norvegicus | 4p9sA | 0.865 |
| gcvT | AFS48830.1 | 0.64 | 0.80 ± 0.09 | dmgdh | Rattus norvegicus | 4p9sA | 0.894 |
| gcvT | AGM40509.1 | 0.55 | 0.79 ± 0.09 | dmgdh | Rattus norvegicus | 4p9sA | 0.887 |
| gcvT | AHI32422.1 | 0.61 | 0.80 ± 0.09 | dmgdh | Rattus norvegicus | 4p9sA | 0.896 |
| gcvT | WP_053112835.1 | 0.56 | 0.79 ± 0.09 | dmgdh | Rattus norvegicus | 4p9sA | 0.997 |
| gcvT | CBV41552.1 | 0.68 | 0.81 ± 0.09 | dmgdh | Rattus norvegicus | 4p9sA | 0.906 |
| gcvT | WP_071941841.1 | 1.11 | 0.87 ± 0.07 | dmgdh | Rattus norvegicus | 4p9sA | 0.997 |
| gcvT | AAV94935.1 | 1.96 | 0.99 ± 0.04 | dmdA | Ca. P. ubique HTCC1062 | 3tfhA | 0.994 |
| gcvT | AII87408.1 | 1.64 | 0.94 ± 0.05 | dmdA | Ca. P. ubique HTCC1062 | 3tfhA | 0.985 |
| gcvT | ADE40415.1 | 2 | 0.99 ± 0.04 | dmdA | Ca. P. ubique HTCC1062 | 3tfhA | 0.995 |
| gcvT | AHM03102.1 | 1.69 | 0.95 ± 0.05 | dmdA | Ca. P. ubique HTCC1062 | 3tfhA | 0.981 |
| gcvT | WP_071972920.1 | 1.99 | 0.99 ± 0.04 | dmdA | Ca. P. ubique HTCC1062 | 3tfhA | 0.988 |
| gcvT | BAN00949.1 | 1.13 | 0.87 ± 0.07 | dmg | Arthrobacter globiformis | 1pj6A | 0.948 |
| gcvT | WP_053819980.1 | 1.71 | 0.95 ± 0.05 | dmdA | Ca. P. ubique HTCC1062 | 3tfhA | 0.988 |
| gcvT | ABF63906.1 | 1.53 | 0.93 ± 0.06 | dmdA | Ca. P. ubique HTCC1062 | 3tfhA | 0.960 |
| gcvT | AGI71303.1 | 1.65 | 0.95 ± 0.05 | dmdA | Ca. P. ubique HTCC1062 | 3tfhA | 0.960 |
| gcvT | AII85872.1 | 1.52 | 0.93 ± 0.06 | dmdA | Ca. P. ubique HTCC1062 | 3tfhA | 0.960 |
| gcvT | WP_067545452.1 | 1.59 | 0.94 ± 0.05 | dmdA | Ca. P. ubique HTCC1062 | 3tfhA | 0.961 |
| gcvT | ADE39159.1 | 1.50 | 0.92 ± 0.06 | dmdA | Ca. P. ubique HTCC1062 | 3tfhA | 0.950 |
| gcvT | AGI71500.1 | 1.47 | 0.92 ± 0.06 | dmdA | Ca. P. ubique HTCC1062 | 3tfhA | 0.949 |
| gcvT | AFS47213.1 | 1.66 | 0.95 ± 0.05 | dmdA | Ca. P. ubique HTCC1062 | 3tfhA | 0.966 |
| gcvT | AFS48354.1 | 1.60 | 0.94 ± 0.05 | dmdA | Ca. P. ubique HTCC1062 | 3tfhA | 0.963 |
| gcvT | WP_053820730.1 | 0.34 | 0.67 ± 0.13 | dmgdh | Rattus norvegicus | 4p9sA | 0.874 |
| gcvT | WP_065353845.1 | 1.56 | 0.93 ± 0.06 | dmdA | Ca. P. ubique HTCC1062 | 3tfhA | 0.961 |
| gcvT | Ancestral DmdA and non-DmdA sequence | 1.25 | 0.89 ± 0.07 | gcvT | Thermotoga maritima | 1wooA | 0.960 |
| dmdA | Ancestral DmdA sequence | 2 | 0.99 ± 0.04 | dmdA | Ca. P. ubique HTCC10626 | 3tfhA | 0.997 |
| gcvT | Ancestral non-DmdA sequence | 0.76 | 0.82 ± 0.09 | dmgdh | Rattus norvegicus | 4p9sA | 0.940 |
