# Supplementary material for: Evolutionary history of dimethylsulfoniopropionate (DMSP) demethylation enzyme DmdA in marine bacteria
Source: PeerJ. 2020 Sep 10;8:e9861. doi: 10.7717/peerj.9861 (PMC7487153; doi:10.7717/peerj.9861)
Supplement: Supplementary file 10 [file peerj-08-9861-s031.odt]

| Taxonomic group of MRCA | Clade | Age | 95% HPD |
| --- | --- | --- | --- |
| Halobacteriales (455) | Mrca1 | 438 | 311.1 – 572.3 |
| SAR11 (826) | Mrca2 | 827.5 | 588.3 – 1089.8 |
| Alphaproteobacteria (2480) | Mrca3 | 2118.6 | 1543 – 2717.1 |
