# Supplementary material for: Evolutionary history of dimethylsulfoniopropionate (DMSP) demethylation enzyme DmdA in marine bacteria
Source: PeerJ. 2020 Sep 10;8:e9861. doi: 10.7717/peerj.9861 (PMC7487153; doi:10.7717/peerj.9861)
Supplement: Supplementary file 11 [file peerj-08-9861-s032.odt]

| Taxonomy | Identification | PI1 | Mw2 | Instability index3 | Aliphatic index4 | Location (PSORTb v.3.0) |
| --- | --- | --- | --- | --- | --- | --- |
| ASR5 | Root marginal sequences of DmdA family | 6.5* | 41334.4 | 39.5 | stable | 91.32 | Cytoplasmic |
| Ca. P. ubique HTCC1062 | AAZ21068.1 | 6.47* | 41831.81 | 32.73 | stable | 86.1 | Cytoplasmic |
| HIMB59 | AFS48343.1 | 5.17 | 41499.43 | 39.62 | stable | 86.1 | Cytoplasmic |
| HIMB5 | AFS46782.1 | 6.99* | 41692.14 | 39.23 | stable | 91.9 | Cytoplasmic |
| G. antarcticus IMCC3135 | ASJ73090.1 | 4.91 | 43371.33 | 33.58 | stable | 92.19 | Cytoplasmic |
| Ca. puniceispirillum marinum IMCC1322 | ADE38317.1 | 5.55 | 41421.73 | 43.47 | unstable | 92.21 | Cytoplasmic |
| L. methylohalidivorans DSM14336 | AHD01041.1 | 4.93 | 40057.61 | 39.14 | stable | 86.57 | Cytoplasmic |
| R. pomeroyi DSS-3 | AAV95190.1 | 5.27 | 39895.45 | 37.59 | stable | 84.4 | Cytoplasmic |
| Hoeflea sp IMCC20628 | WP_047029467.1 | 4.98 | 40736.4 | 35.67 | stable | 87.91 | Cytoplasmic |
| D. shibae DFL12 | ABV94056.1 | 4.81 | 41294.27 | 39.05 | stable | 90.03 | Cytoplasmic |
| O. temperatus SB1 | WP_049834197.1 | 5.03 | 40693.65 | 27.46 | stable | 85.68 | - |
| O. antarcticus 307 | AGI68776.1 | 5.32 | 40692.51 | 26.43 | stable | 86.75 | Cytoplasmic |
| O. arcticus 238 | AGI72139.1 | 5.5 | 40570.47 | 28.03 | stable | 87.32 | Cytoplasmic |
| R. elongatum DSM19469 | AHM05061.1 | 5.49 | 40459.36 | 40.05 | unstable | 88.15 | Cytoplasmic |
| M. algicola DG898 | WP_048536000.1 | 5.12 | 40770.54 | 35.43 | stable | 85.17 | Cytoplasmic |
| Jannaschia sp CCS1 | ABD55296.1 | 5.05 | 40852.7 | 42.04 | unstable | 86.57 | Cytoplasmic |
| P. gallaeciensis JL2886 | WP_065273401.1 | 5.58 | 41133.97 | 40.89 | unstable | 82.07 | Cytoplasmic |
| Ruegeria sp TM1040 | ABF64177.1 | 5.78 | 42951.08 | 38.16 | stable | 82.26 | Cytoplasmic |
| T. oomphalii DOK1-4 | WP_076627280.1 | 5.24 | 41152 | 41.96 | unstable | 84.71 | Cytoplasmic |
| R. denitrificans Och114 | ABG31871.1 | 5.15 | 40785.38 | 30.1 | stable | 86.62 | Cytoplasmic |
| R. litoralis Och149 | AEI94210.1 | 5.09 | 40648.28 | 27.93 | stable | 87.71 | Cytoplasmic |
| ASR | Root marginal sequence of Dmda and non-DmdA families | 4.43 | 31948.03 | 45.46 | unstable | 89.16 | Cytoplasmic |
| ASR | Root marginal sequence of non-DmdA family | 4.3 | 40334.85 | 43.72 | unstable | 84.44 | Cytoplasmic |
| N10 | Non-DmdA tree | 4.69 | 39908.25 | 41.21 | unstable | 92.38 |  |
| N3 | DmdA tree | 4.85 | 41479.21 | 40.25 | unstable | 86.56 |  |
