# Supplementary material for: Evolutionary history of dimethylsulfoniopropionate (DMSP) demethylation enzyme DmdA in marine bacteria
Source: PeerJ. 2020 Sep 10;8:e9861. doi: 10.7717/peerj.9861 (PMC7487153; doi:10.7717/peerj.9861)
Supplement: Supplementary file 12 [file peerj-08-9861-s033.odt]

| Model | ω1 | ω2 | -lnL1 | LRT2 | P-value |
| --- | --- | --- | --- | --- | --- |
| One ω (one-ratio) | 0.08518 | NA | -14580.019867 | NA | NA |
| Two ω (two-ratio) | 0.0767 | 0.1494 | -14568.131038 | 23.777658 | 0.0 |
| 38 ω (free-ratio) | * | * | -14428.881747 | 302.27624 | 0 |
