# Supplementary material for: Evolutionary history of dimethylsulfoniopropionate (DMSP) demethylation enzyme DmdA in marine bacteria
Source: PeerJ. 2020 Sep 10;8:e9861. doi: 10.7717/peerj.9861 (PMC7487153; doi:10.7717/peerj.9861)
Supplement: Supplementary file 13 [file peerj-08-9861-s034.odt]

| Branch | Ho (-lnL)1 | Ha (-lnL)2 | LRT3 | P-value4 | CorrectedP-value5 | Pos. Selected sites* (BEB>0.95) |
| --- | --- | --- | --- | --- | --- | --- |
| ADE38317.1 | -14465.244 | -14463.099 | 4.290 | 0.038 | 0.767 | NA |
| AAV95190.1 | -14476.763 | -14476.763 | 0 | 1 | 1 | NA |
| AHD01041.1 | -14476.763 | -14476.763 | 0 | 1 | 1 | NA |
| WP_047029467.1 | -14476.763 | -14437.565 | 78.397 | 0.00 | 0.00 | 7V; 17E; 47H; 65D; 68Y; 87Y; 89A; 152K; 157M; 163N; 203V; 279G; 290P; 319T; 320H |
| WP_048536000.1 | -14476.763 | -14476.763 | 0 | 1 | 1 | NA |
| AHM05061.1 | -14466.948 | -14460.844 | 12.206 | 0.000 | 0.000 | 17E ; 152K; 178E; 285V |
| ABF64177.1 | -14476.763 | -14476.763 | 0 | 1 | 1 | NA |
| WP_065273401.1 | -14476.763 | 14476.763 | 0 | 1 | 1 | NA |
| WP_076627280.1 | -14476.763 | 14476.763 | 0 | 1 | 1 | NA |
| AEI94210.1 | -14476.763 | -14476.763 | 0 | 1 | 1 | NA |
| ABG31871.1 | -14476.763 | -14476.763 | 0 | 1 | 1 | NA |
| ABD55296.1 | -14476.764 | -14476.764 | 0 | 1 | 1 | NA |
| WP_049834197.1 | -14476.763 | -14476.763 | 0 | 1 | 1 | NA |
| AGI72139.1 | -14476.763 | -14476.763 | 0 | 1 | 1 | NA |
| AGI68776.1 | -14476.763 | -14476.763 | 0 | 1 | 1 | NA |
| ABV94056.1 | -14462.942 | -14454.885 | 16.112 | 0.000 | 0.000 | 87Y; 152K; 243N; 247L; 257F |
| ASJ730990.1 | -14463.474 | -14461.176 | 4.595 | 0.032 | 0.641 | NA |
| AAZ21068.1 | -14465.122 | -14462.171 | 5.902 | 0.015 | 0.302 | NA |
| AFS46782.1 | -14467.961 | -14464.484 | 6.954 | 0.008 | 0.167 | NA |
| AFS48343.1 | -14460.566 | -14425.923 | 31.802 | 0.000 | 0.000 | 4S; 5A; 9S; 35S; 38V; 70T; 83D; 84H; 85I; 91V; 94D; 95Q; 103L; 109P; 119T; 139T; 155E; 158K; 168N; 176N; 179F; 210L; 211R; 217G;  231S; 253A; 259P; 270Q; 274V; 277S; 292N; 298T; 305S; 311C; 321T |
| Ancestral branch to the DmdA clade | -28761.935 | -28758.081 | 7.7084 | 0.005 | 0.010 | 39Q |
| Ancestral branch to the non-DmdA clade | -28770.533 | -28766.874 | 7.3182 | 0.006 | 0.013 | - |
