# Supplementary material for: Evolutionary history of dimethylsulfoniopropionate (DMSP) demethylation enzyme DmdA in marine bacteria
Source: PeerJ. 2020 Sep 10;8:e9861. doi: 10.7717/peerj.9861 (PMC7487153; doi:10.7717/peerj.9861)
Supplement: Supplementary file 14 [file peerj-08-9861-s035.odt]

| Model | NP1 | ω2 | Site class 0 | Site class 1 | Site class 2 | K3 | -LnL4 | LTR5 | P-value | Divergent sites* |
| --- | --- | --- | --- | --- | --- | --- | --- | --- | --- | --- |
|  |  |  | ω0 | po | ω1 | p1 | ω2 | p2 |  |  |  |  |  |
| M0 | 95 | 0.053 |  |  |  |  |  |  | 1.341 | -28818.866 | na | na |  |
| M3 (k=3) | 99 | 0.058 | 0.006 | 0.238 | 0.045 | 0.506 | 0.132 | 0.255 | 1.342 | -28079.171 | 1479.391 | 0.00 |  |
| MD (k=3) | 100 |  | 0.006 | 0.235 | 0.042 | 0.492 | ω2a0.100 ω2b:0.169 | 0.272 | 1.337 | -28061.808 | 34.725 | 0.00 | 2V, 9Q, 12E, 14Y, 16Q, 17A, 28S, 32N, 36N, 37H, 52E, 57D, 58Y, 60T, 62L, 69S, 70Q, 71A, 72K, 73D, 77Y, 85Q, 98K, 101T, 118I, 127T, 132N, 142F, 146K, 147R, 150E, 156K, 157R, 158Y, 159A, 161N, 163H, 164E, 166L, 185D, 187V, 188Q, 192Q, 194L, 198K, 199D, 211S, 218M, 226A, 229S, 230P, 240K, 241K, 242S, 244S, 247I, 248M, 250D, 253T, 254L, 258C, 259Y, 264G, 265K, 272Q, 273L, 274D, 275Q, 276D, 277L, 278K, 280Q, 283K, 285T, 286N, 287L |
