# Supplementary material for: Evolutionary history of dimethylsulfoniopropionate (DMSP) demethylation enzyme DmdA in marine bacteria
Source: PeerJ. 2020 Sep 10;8:e9861. doi: 10.7717/peerj.9861 (PMC7487153; doi:10.7717/peerj.9861)
Supplement: Supplementary file 15 [file peerj-08-9861-s036.odt]

| Model | ω1 | ω2 | -lnL4 | LRT5 | P-value |
| --- | --- | --- | --- | --- | --- |
| One ω (one-ratio) | 0.05348 | NA | -31199.102911 | NA | NA |
| Two ω (two-ratio)1 | 0.05367 | 999 | -31197.315923 | 3.573976 | 0.0587 |
| Two ω (two-ratio)2 | 0.05399 | 0.00 | -31197.838823 | 2.528176 | 0.1118 |
| Two ω (two-ratio)3 | 0.05362 | 999 | -31197.199937 | 0.000012 | 0.9972 |
