# Supplementary material for: Evolutionary history of dimethylsulfoniopropionate (DMSP) demethylation enzyme DmdA in marine bacteria
Source: PeerJ. 2020 Sep 10;8:e9861. doi: 10.7717/peerj.9861 (PMC7487153; doi:10.7717/peerj.9861)
Supplement: Supplementary file 16 [file peerj-08-9861-s037.odt]

Table 1. Up: Top ten analogs identified by LOMETS for threading alignments. Case of AAV94935.1. In the middle: Model predicted by I-TASSER. Down: Top ten identified structural analogs in PDB by TM-align

| Rank | Class | Gene name | Organism | PDB ID | Iden1 (%)a | Iden2 (%)b | Covc | Norm. Z-scored |
| --- | --- | --- | --- | --- | --- | --- | --- | --- |
| 1 | AMTe | dmdAf | Pelagibacter ubique (HTCC1062) | 3tfhA | 40 | 40 | 1.00 | 4.58 |
| 2 | AMT | dmdA | Pelagibacter ubique (HTCC1062) | 3tfhA | 39 | 40 | 1.00 | 5.33 |
| 3 | AMT | dmdA | Pelagibacter ubique (HTCC1062) | 3tfhA | 38 | 40 | 0.99 | 5.44 |
| 4 | AMT | dmdA | Pelagibacter ubique (HTCC1062) | 3tfh | 39 | 40 | 0.99 | 3.25 |
| 5 | Oxidoreductase | dmgdHh | Rattus norvegicus | 4p9sA | 21 | 25 | 0.97 | 2.40 |
| 6 | AMT | dmdA | Pelagibacter ubique (HTCC1062) | 3tfhA | 38 | 40 | 0.98 | 5.58 |
| 7 | AMT | dmdA | Pelagibacter ubique (HTCC1062) | 3tfh | 40 | 40 | 0.98 | 3.61 |
| 8 | AMT | gcvTg | Bacillus subtilis | 1yx2A | 24 | 24 | 0.96 | 6.66 |
| 9 | AMT | dmdA | Pelagibacter ubique (HTCC1062) | 3tfhA | 39 | 40 | 0.99 | 7.29 |
| 10 | AMT | dmdA | Pelagibacter ubique (HTCC1062) | 3tfhA | 39 | 40 | 1.00 | 6.72 |

aIden1 is the percentage sequence identity of the templates in the threading aligned region with the query sequence

bIden2 is the percentage sequence identity of the whole template chains with query sequence 

cCov-Represents the coverage of the threading alignment and is equal to the number of aligned residues divided by the length of the query protein

dNorm. Z-score is the normalized Z-score of the threading alignments. Alignment with a normalized Z-score>1 means a good alignment and vice versa.

eAminomethyltransferase

fDmdA DMSP-dependent demethylase

gGlycine cleavage system T protein

hDimethylglycine dehydrogenase complexed with tetrahydrofolate 

Predicted model: C-sore= 1.96; TM-score=0.99 dev= 0.04

| Rank | Class | Gene name | Organism | PDB ID | TM-scorea | RMSDb | IDENc | Covd |
| --- | --- | --- | --- | --- | --- | --- | --- | --- |
| 1 | AMT | dmdA | Pelagibacter ubique (HTCC1062) | 3tfhA | 0.994 | 0.43 | 0.393 | 0.997 |
| 2 | Oxidoreductase | dmgdH | Rattus norvegicus | 4p9sA2 | 0.927 | 1.80 | 0.217 | 0.981 |
| 3 | Oxidoreductase | dmge | Arthrobacter globiformis | 1pj6A | 0.914 | 2.16 | 0.249 | 0.984 |
| 4 | AMT | gcvT | Pyrococcus horikoshii | 1v5vA | 0.901 | 1.96 | 0.240 | 0.965 |
| 5 | AMT | gcvT | Thermotoga maritima | 1wooA | 0.898 | 1.73 | 0.261 | 0.948 |
| 6 | Oxidoreductase | soxAf | Stenotrophomonas maltophilia | 2gagA | 0.898 | 2.30 | 0.224 | 0.976 |
| 7 | Oxidoreductase | soxA | Corynebacterium sp. | 1vrqA | 0.897 | 2.28 | 0.235 | 0.976 |
| 8 | AMT | AMT | Homo sapiens | 1wsrA | 0.888 | 2.09 | 0.222 | 0.956 |
| 9 | AMT | gcvT | Bartonella henselae | 3girA | 0.882 | 1.96 | 0.237 | 0.943 |
| 10 | AMT | gcvT | Bacillus subtilis | 1yx2B | 0.877 | 2.09 | 0.245 | 0.946 |

abIt is a standard for measuring structural similarity between two structures

cIt is the percentage sequence identity in the structurally aligned region 

dIt represents the coverage of the alignment by TM-align and is equal to the number of structurally aligned residues divided by length of the query protein

eDimethylglycine oxidase

fHeterotetrameric sarcosine oxidase alpha-subunit

Table 2. Up: Top ten analogs identified by LOMETS for threading alignments. Case of AII87408.1. In the middle: Model predicted by I-TASSER. Down: Top ten identified structural analogs in PDB by TM-align

| Rank | Class | Gene name | Organism | PDB ID | Iden1 (%)a | Iden2 (%)b | Covc | Norm. Z-scored |
| --- | --- | --- | --- | --- | --- | --- | --- | --- |
| 1 | AMTe | dmdAf | Pelagibacter ubique (HTCC1062) | 3tfhA | 36 | 37 | 0.99 | 4.64 |
| 2 | AMT | dmdA | Pelagibacter ubique (HTCC1062) | 3tfhA | 36 | 37 | 1.00 | 5.27 |
| 3 | AMT | dmdA | Pelagibacter ubique (HTCC1062) | 3tfhA | 36 | 37 | 0.99 | 5.57 |
| 4 | Oxidoreductase | dmgdHh | Rattus norvegicus | 4p9s | 22 | 31 | 0.96 | 3.26 |
| 5 | Oxidoreductase | dmgdH | Rattus norvegicus | 4p9sA | 21 | 24 | 0.98 | 2.42 |
| 6 | AMT | dmdA | Pelagibacter ubique (HTCC1062) | 3tfhA | 36 | 37 | 0.99 | 5.74 |
| 7 | AMT | dmdA | Pelagibacter ubique (HTCC1062) | 3tfh | 36 | 37 | 0.98 | 3.65 |
| 8 | AMT | gcvTg | Bacillus subtilis | 1yx2A | 26 | 28 | 0.96 | 6.47 |
| 9 | AMT | dmdA | Pelagibacter ubique (HTCC1062) | 3tfhA | 37 | 37 | 0.99 | 7.38 |
| 10 | AMT | dmdA | Pelagibacter ubique (HTCC1062) | 3tfhA | 36 | 37 | 0.99 | 6.67 |

aIden1 is the percentage sequence identity of the templates in the threading aligned region with the query sequence

bIden2 is the percentage sequence identity of the whole template chains with query sequence 

cCov-Represents the coverage of the threading alignment and is equal to the number of aligned residues divided by the length of the query protein

dNorm. Z-score is the normalized Z-score of the threading alignments. Alignment with a normalized Z-score>1 means a good alignment and vice versa.

eAminomethyltransferase

fDmdA DMSP-dependent demethylase

gGlycine cleavage system T protein

hDimethylglycine dehydrogenase complexed with tetrahydrofolate 

Predicted model: C-sore= 1.64; TM-score=0.94 dev= 0.05

| Rank | Class | Gene name | Organism | PDB ID | TM-scorea | RMSDb | IDENc | Covd |
| --- | --- | --- | --- | --- | --- | --- | --- | --- |
| 1 | AMT | dmdA | Pelagibacter ubique (HTCC1062) | 3tfhA | 0.985 | 0.81 | 0.365 | 0.997 |
| 2 | Oxidoreductase | dmgdH | Rattus norvegicus | 4p9sA2 | 0.938 | 1.60 | 0.230 | 0.981 |
| 3 | Oxidoreductase | dmge | Arthrobacter globiformis | 1pj6A | 0.920 | 2.01 | 0.260 | 0.981 |
| 4 | AMT | gcvT | Pyrococcus horikoshii | 1v5vA | 0.908 | 1.86 | 0.225 | 0.965 |
| 5 | AMT | gcvT | Thermotoga maritima | 1wooA | 0.902 | 1.68 | 0.244 | 0.948 |
| 6 | Oxidoreductase | soxAf | Stenotrophomonas maltophilia | 2gagA | 0.899 | 2.28 | 0.213 | 0.973 |
| 7 | Oxidoreductase | soxA | Corynebacterium sp. | 1vrqA | 0.898 | 2.25 | 0.221 | 0.973 |
| 8 | AMT | AMT | Homo sapiens | 1wsrA | 0.893 | 1.96 | 0.231 | 0.954 |
| 9 | AMT | gcvT | Bartonella henselae | 3girA | 0.886 | 1.89 | 0.216 | 0.943 |
| 10 | AMT | gcvT | Bacillus subtilis | 1yx2B | 0.882 | 2.07 | 0.266 | 0.948 |

abIt is a standard for measuring structural similarity between two structures

cIt is the percentage sequence identity in the structurally aligned region 

dIt represents the coverage of the alignment by TM-align and is equal to the number of structurally aligned residues divided by length of the query protein

eDimethylglycine oxidase

fHeterotetrameric sarcosine oxidase alpha-subunit

Table 3. Up: Top ten analogs identified by LOMETS for threading alignments. Case of ADE40415.1. In the middle: Model predicted by I-TASSER. Down: Top ten identified structural analogs in PDB by TM-align

| Rank | Class | Gene name | Organism | PDB ID | Iden1 (%)a | Iden2 (%)b | Covc | Norm. Z-scored |
| --- | --- | --- | --- | --- | --- | --- | --- | --- |
| 1 | AMTe | dmdAf | Pelagibacter ubique (HTCC1062) | 3tfhA | 38 | 39 | 1.00 | 4.72 |
| 2 | AMT | dmdA | Pelagibacter ubique (HTCC1062) | 3tfhA | 38 | 39 | 1.00 | 5.45 |
| 3 | AMT | dmdA | Pelagibacter ubique (HTCC1062) | 3tfhA | 37 | 39 | 0.99 | 5.59 |
| 4 | AMT | dmdA | Pelagibacter ubique (HTCC1062) | 3tfh | 38 | 39 | 0.99 | 3.28 |
| 5 | Oxidoreductase | dmgdHh | Rattus norvegicus | 4p9sA | 24 | 27 | 0.98 | 2.41 |
| 6 | AMT | dmdA | Pelagibacter ubique (HTCC1062) | 3tfhA | 38 | 39 | 0.99 | 5.78 |
| 7 | AMT | dmdA | Pelagibacter ubique (HTCC1062) | 3tfh | 37 | 39 | 0.98 | 3.63 |
| 8 | AMT | gcvTg | Bacillus subtilis | 1yx2A | 24 | 25 | 0.95 | 6.60 |
| 9 | AMT | dmdA | Pelagibacter ubique (HTCC1062) | 3tfhA | 38 | 39 | 1.00 | 7.52 |
| 10 | AMT | dmdA | Pelagibacter ubique (HTCC1062) | 3tfhA | 38 | 39 | 1.00 | 6.89 |

aIden1 is the percentage sequence identity of the templates in the threading aligned region with the query sequence

bIden2 is the percentage sequence identity of the whole template chains with query sequence 

cCov-Represents the coverage of the threading alignment and is equal to the number of aligned residues divided by the length of the query protein

dNorm. Z-score is the normalized Z-score of the threading alignments. Alignment with a normalized Z-score>1 means a good alignment and vice versa.

eAminomethyltransferase

fDmdA DMSP-dependent demethylase

gGlycine cleavage system T protein

hDimethylglycine dehydrogenase complexed with tetrahydrofolate 

Predicted model: C-sore= 2; TM-score=0.99 dev= 0.04

| Rank | Class | Gene name | Organism | PDB ID | TM-scorea | RMSDb | IDENc | Covd |
| --- | --- | --- | --- | --- | --- | --- | --- | --- |
| 1 | AMT | dmdA | Pelagibacter ubique (HTCC1062) | 3tfhA | 0.995 | 0.37 | 0.375 | 0.997 |
| 2 | Oxidoreductase | dmgdH | Rattus norvegicus | 4p9sA2 | 0.925 | 1.88 | 0.235 | 0.981 |
| 3 | Oxidoreductase | dmge | Arthrobacter globiformis | 1pj6A | 0.908 | 2.25 | 0.254 | 0.981 |
| 4 | AMT | gcvT | Pyrococcus horikoshii | 1v5vA | 0.900 | 1.94 | 0.262 | 0.962 |
| 6 | Oxidoreductase | soxAf | Stenotrophomonas maltophilia | 2gagA | 0.895 | 2.31 | 0.212 | 0.970 |
| 7 | Oxidoreductase | soxA | Corynebacterium sp. | 1vrqA | 0.894 | 2.41 | 0.220 | 0.976 |
|  | AMT | gcvT | Thermotoga maritima | 1wooA | 0.893 | 1.74 | 0.276 | 0.943 |
| 8 | AMT | AMT | Homo sapiens | 1wsrA | 0.887 | 2.08 | 0.199 | 0.954 |
| 9 | AMT | gcvT | Bartonella henselae | 3girA | 0.881 | 1.93 | 0.219 | 0.940 |
| 10 | AMT | gcvT | Bacillus subtilis | 1yx2B | 0.875 | 2.09 | 0.247 | 0.943 |

abIt is a standard for measuring structural similarity between two structures

cIt is the percentage sequence identity in the structurally aligned region 

dIt represents the coverage of the alignment by TM-align and is equal to the number of structurally aligned residues divided by length of the query protein

eDimethylglycine oxidase

fHeterotetrameric sarcosine oxidase alpha-subunit

Table 4. Up: Top ten analogs identified by LOMETS for threading alignments. Case of BAN00949.1. In the middle: Model predicted by I-TASSER. Down: Top ten identified structural analogs in PDB by TM-align

| Rank | Class | Gene name | Organism | PDB ID | Iden1 (%)a | Iden2 (%)b | Covc | Norm. Z-scored |
| --- | --- | --- | --- | --- | --- | --- | --- | --- |
| 1 | AMTe | gcvT | Thermotoga maritima (ATCC 43589) | 1wopA | 25 | 25 | 0.88 | 4.29 |
| 2 | AMTe | dmdAf | Pelagibacter ubique (HTCC1062) | 3tfhA | 33 | 30 | 0.90 | 5.18 |
| 3 | AMTe | dmdA | Pelagibacter ubique (HTCC1062) | 3tfhA | 31 | 30 | 0.90 | 5.03 |
| 4 | Oxidoreductase | dmgi | Arthrobacter globiformis | 1pj6 | 23 | 27 | 0.97 | 3.32 |
| 5 | Oxidoreductase | dmgdHh | Rattus norvegicus | 4p9sA | 20 | 23 | 0.92 | 2.40 |
| 6 | AMTe | dmdA | Pelagibacter ubique (HTCC1062) | 3tfhA | 31 | 30 | 0.90 | 5.14 |
| 7 | Oxidoreductase | dmg | Arthrobacter globiformis | 1pj6 | 23 | 27 | 0.95 | 3.62 |
| 8 | AMTe | gcvTg | Bacillus subtilis | 1yx2A | 25 | 22 | 0.87 | 6.32 |
| 9 | AMTe | dmdA | Pelagibacter ubique (HTCC1062) | 3tfhA | 32 | 30 | 0.91 | 6.84 |
| 10 | AMTe | dmdA | Pelagibacter ubique (HTCC1062) | 3tfhA | 32 | 30 | 0.91 | 6.17 |

aIden1 is the percentage sequence identity of the templates in the threading aligned region with the query sequence

bIden2 is the percentage sequence identity of the whole template chains with query sequence 

cCov-Represents the coverage of the threading alignment and is equal to the number of aligned residues divided by the length of the query protein

dNorm. Z-score is the normalized Z-score of the threading alignments. Alignment with a normalized Z-score>1 means a good alignment and vice versa.

eAminomethyltransferase

fDmdA DMSP-dependent demethylase

gGlycine cleavage system T protein

hDimethylglycine dehydrogenase complexed with tetrahydrofolate 

iDimethylglycine oxidase

Predicted model: C-sore= 1.13; TM-score=0.87 dev= 0.07

| Rank | Class | Gene name | Organism | PDB ID | TM-scorea | RMSDb | IDENc | Covd |
| --- | --- | --- | --- | --- | --- | --- | --- | --- |
| 1 | Oxidoreductase | dmg | Arthrobacter globiformis | 1pj6A | 0.948 | 1.62 | 0.229 | 0.988 |
| 2 | Oxidoreductase | dmgdH | Rattus norvegicus | 4p9sA2 | 0.943 | 1.63 | 0.192 | 0.985 |
| 3 | AMT | dmdA | Pelagibacter ubique HTCC1062 | 3tfhA | 0.879 | 1.38 | 0.326 | 0.908 |
| 6 | Oxidoreductase | soxAe | Stenotrophomonas maltophilia | 2gagA | 0.859 | 2.18 | 0.180 | 0.920 |
| 7 | Oxidoreductase | soxA | Corynebacterium sp. | 1vrqA | 0.858 | 2.21 | 0.185 | 0.920 |
|  | AMT | gcvT | Thermotoga maritima | 1wooA | 0.853 | 1.43 | 0.254 | 0.883 |
|  | AMT | gcvT | Pyrococcus horikoshii | 1v5vA | 0.850 | 1.96 | 0.238 | 0.898 |
| 8 | AMT | AMT | Homo sapiens | 1wsrA | 0.839 | 1.77 | 0.208 | 0.883 |
| 9 | AMT | gcvT | Bartonella henselae | 3girA | 0.831 | 1.85 | 0.207 | 0.878 |
| 10 | AMT | gcvT | Bacillus subtilis | 1yx2B | 0.825 | 1.70 | 0.247 | 0.866 |

abIt is a standard for measuring structural similarity between two structures

cIt is the percentage sequence identity in the structurally aligned region 

dIt represents the coverage of the alignment by TM-align and is equal to the number of structurally aligned residues divided by length of the query protein

eHeterotetrameric sarcosine oxidase alpha-subunit

Table 5. Up: Top ten analogs identified by LOMETS for threading alignments. Case of AII85872.1. In the middle: Model predicted by I-TASSER. Down: Top ten identified structural analogs in PDB by TM-align

| Rank | Class | Gene name | Organism | PDB ID | Iden1 (%)a | Iden2 (%)b | Covc | Norm. Z-scored |
| --- | --- | --- | --- | --- | --- | --- | --- | --- |
| 1 | AMTe | dmdAf | Pelagibacter ubique (HTCC1062) | 3tfhA | 36 | 36 | 0.97 | 4.77 |
| 2 | AMT | dmdA | Pelagibacter ubique (HTCC1062) | 3tfhA | 36 | 36 | 0.97 | 5.42 |
| 3 | AMT | dmdA | Pelagibacter ubique (HTCC1062) | 3tfhA | 36 | 36 | 0.97 | 5.49 |
| 4 | Oxidoreductase | dmgi | Arthrobacter globiformis | 1pj6 | 26 | 30 | 0.98 | 3.25 |
| 5 | Oxidoreductase | dmgdHh | Rattus norvegicus | 4p9sA | 22 | 26 | 0.97 | 2.40 |
| 6 | AMT | dmdA | Pelagibacter ubique (HTCC1062) | 3tfhA | 36 | 36 | 0.97 | 5.73 |
| 7 | Oxidoreductase | dmg | Arthrobacter globiformis | 1pj6 | 25 | 30 | 0.97 | 3.63 |
| 8 | AMT | gcvTg | Bacillus subtilis | 1yx2A | 28 | 28 | 0.93 | 6.47 |
| 9 | AMT | dmdA | Pelagibacter ubique (HTCC1062) | 3tfhA | 36 | 36 | 0.97 | 7.77 |
| 10 | AMT | dmdA | Pelagibacter ubique (HTCC1062) | 3tfhA | 36 | 36 | 0.97 | 6.72 |

aIden1 is the percentage sequence identity of the templates in the threading aligned region with the query sequence

bIden2 is the percentage sequence identity of the whole template chains with query sequence 

cCov-Represents the coverage of the threading alignment and is equal to the number of aligned residues divided by the length of the query protein

dNorm. Z-score is the normalized Z-score of the threading alignments. Alignment with a normalized Z-score>1 means a good alignment and vice versa.

eAminomethyltransferase

fDmdA DMSP-dependent demethylase

gGlycine cleavage system T protein

hDimethylglycine dehydrogenase complexed with tetrahydrofolate 

iDimethylglycine oxidase

Predicted model: C-sore= 1.52; TM-score=0.93 dev= 0.06

| Rank | Class | Gene name | Organism | PDB ID | TM-scorea | RMSDb | IDENc | Covd |
| --- | --- | --- | --- | --- | --- | --- | --- | --- |
| 1 | AMT | dmdA | Pelagibacter ubique (HTCC1062) | 3tfhA | 0.960 | 0.76 | 0.358 | 0.971 |
| 2 | Oxidoreductase | dmgdH | Rattus norvegicus | 4p9sA2 | 0.929 | 1.85 | 0.228 | 0.982 |
| 3 | Oxidoreductase | dmg | Arthrobacter globiformis | 1pj6A | 0.926 | 1.95 | 0.255 | 0.982 |
| 6 | Oxidoreductase | soxAe | Stenotrophomonas maltophilia | 2gagA | 0.896 | 2.25 | 0.224 | 0.968 |
| 7 | AMT | gcvT | Pyrococcus horikoshii ATCC700860 | 1v5vA | 0.895 | 2.10 | 0.269 | 0.958 |
|  | Oxidoreductase | soxA | Corynebacterium sp. | 1vrqA | 0.893 | 2.25 | 0.222 | 0.966 |
|  | AMT | gcvT | Thermotoga maritima | 1wooA | 0.888 | 1.73 | 0.256 | 0.934 |
| 8 | AMT | AMT | Homo sapiens | 1wsrA | 0.884 | 2.02 | 0.226 | 0.945 |
| 9 | AMT | gcvT | Bartonella henselae | 3girA | 0.875 | 1.86 | 0.218 | 0.929 |
| 10 | AMT | gcvT | Bacillus subtilis | 1yx2B | 0.864 | 1.99 | 0.279 | 0.924 |

abIt is a standard for measuring structural similarity between two structures

cIt is the percentage sequence identity in the structurally aligned region 

dIt represents the coverage of the alignment by TM-align and is equal to the number of structurally aligned residues divided by length of the query protein

eHeterotetrameric sarcosine oxidase alpha-subunit

Table 6. Up: Top ten analogs identified by LOMETS for threading alignments. Case of WP_071972920. In the middle: Model predicted by I-TASSER. Down: Top ten identified structural analogs in PDB by TM-align

| Rank | Class | Gene name | Organism | PDB ID | Iden1 (%)a | Iden2 (%)b | Covc | Norm. Z-scored |
| --- | --- | --- | --- | --- | --- | --- | --- | --- |
| 1 | AMTe | dmdAf | Pelagibacter ubique (HTCC1062) | 3tfhA | 35 | 34 | 0.99 | 4.54 |
| 2 | AMT | dmdA | Pelagibacter ubique (HTCC1062) | 3tfhA | 35 | 34 | 0.99 | 5.33 |
| 3 | AMT | dmdA | Pelagibacter ubique (HTCC1062) | 3tfhA | 34 | 34 | 0.99 | 5.59 |
| 4 | Oxidoreductase | dmgi | Arthrobacter globiformis | 3tfh | 34 | 34 | 0.99 | 3.31 |
| 5 | Oxidoreductase | dmgdHh | Rattus norvegicus | 4p9sA | 19 | 23 | 0.97 | 2.40 |
| 6 | AMT | dmdA | Pelagibacter ubique (HTCC1062) | 3tfhA | 34 | 34 | 0.99 | 5.62 |
| 7 | Oxidoreductase | mgdHh | Rattus norvegicus | 4p9sA | 19 | 23 | 0.96 | 3.59 |
| 8 | AMT | gcvTg | Bacillus subtilis | 1yx2A | 24 | 25 | 0.95 | 6.58 |
| 9 | AMT | dmdA | Pelagibacter ubique (HTCC1062) | 3tfhA | 35 | 34 | 0.99 | 7.07 |
| 10 | AMT | dmdA | Pelagibacter ubique (HTCC1062) | 3tfhA | 34 | 34 | 0.99 | 6.73 |

aIden1 is the percentage sequence identity of the templates in the threading aligned region with the query sequence

bIden2 is the percentage sequence identity of the whole template chains with query sequence 

cCov-Represents the coverage of the threading alignment and is equal to the number of aligned residues divided by the length of the query protein

dNorm. Z-score is the normalized Z-score of the threading alignments. Alignment with a normalized Z-score>1 means a good alignment and vice versa.

eAminomethyltransferase

fDmdA DMSP-dependent demethylase

gGlycine cleavage system T protein

hDimethylglycine dehydrogenase complexed with tetrahydrofolate 

iDimethylglycine oxidase

Predicted model: C-sore= 1.99; TM-score=0.99 dev= 0.04

| Rank | Class | Gene name | Organism | PDB ID | TM-scorea | RMSDb | IDENc | Covd |
| --- | --- | --- | --- | --- | --- | --- | --- | --- |
| 1 | AMT | dmdA | Pelagibacter ubique (HTCC1062) | 3tfhA | 0.988 | 0.42 | 0.346 | 0.992 |
| 2 | Oxidoreductase | dmgdH | Rattus norvegicus | 4p9sA2 | 0.921 | 1.82 | 0.197 | 0.976 |
| 3 | Oxidoreductase | dmg | Arthrobacter globiformis | 1pj6A | 0.908 | 2.18 | 0.224 | 0.978 |
| 4 | AMT | gcvT | Pyrococcus horikoshii ATCC700860 | 1v5vA | 0.901 | 2.06 | 0.240 | 0.968 |
| 5 | Oxidoreductase | soxAe | Stenotrophomonas maltophilia | 2gagA | 0.893 | 2.31 | 0.242 | 0.968 |
| 6 | Oxidoreductase | soxA | Corynebacterium sp. | 1vrqA | 0.892 | 2.27 | 0.237 | 0.968 |
| 7 | AMT | gcvT | Thermotoga maritima | 1wooA | 0.890 | 1.80 | 0.244 | 0.943 |
| 8 | AMT | AMT | Homo sapiens | 1wsrA | 0.884 | 2.02 | 0.208 | 0.949 |
| 9 | AMT | gcvT | Bartonella henselae | 3girA | 0.878 | 1.90 | 0.202 | 0.935 |
| 10 | AMT | gcvT | Bacillus subtilis | 1yx2B | 0.869 | 2.16 | 0.239 | 0.941 |

abIt is a standard for measuring structural similarity between two structures

cIt is the percentage sequence identity in the structurally aligned region 

dIt represents the coverage of the alignment by TM-align and is equal to the number of structurally aligned residues divided by length of the query protein

eHeterotetrameric sarcosine oxidase alpha-subunit

Table 7. Up: Top ten analogs identified by LOMETS for threading alignments. Case of AHM03102.1. In the middle: Model predicted by I-TASSER. Down: Top ten identified structural analogs in PDB by TM-align

| Rank | Class | Gene name | Organism | PDB ID | Iden1 (%)a | Iden2 (%)b | Covc | Norm. Z-scored |
| --- | --- | --- | --- | --- | --- | --- | --- | --- |
| 1 | AMTe | dmdAf | Pelagibacter ubique (HTCC1062) | 3tfhA | 37 | 36 | 0.99 | 4.64 |
| 2 | AMT | dmdA | Pelagibacter ubique (HTCC1062) | 3tfhA | 37 | 36 | 0.99 | 5.36 |
| 3 | AMT | dmdA | Pelagibacter ubique (HTCC1062) | 3tfhA | 36 | 36 | 0.99 | 5.47 |
| 4 | Oxidoreductase | dmgi | Rattus norvegicus | 4p9s | 21 | 30 | 0.97 | 3.25 |
| 5 | Oxidoreductase | dmgdHh | Rattus norvegicus | 4p9sA | 20 | 24 | 0.98 | 2.42 |
| 6 | AMT | dmdA | Pelagibacter ubique (HTCC1062) | 3tfhA | 36 | 36 | 0.98 | 5.68 |
| 7 | Oxidoreductase | dmgdHh | Rattus norvegicus | 4p9s | 19 | 30 | 0.97 | 3.59 |
| 8 | AMT | gcvTg | Bacillus subtilis | 1yx2A | 24 | 26 | 0.95 | 6.66 |
| 9 | AMT | dmdA | Pelagibacter ubique (HTCC1062) | 3tfhA | 37 | 36 | 0.99 | 7.38 |
| 10 | AMT | dmdA | Pelagibacter ubique (HTCC1062) | 3tfhA | 36 | 36 | 0.99 | 6.61 |

aIden1 is the percentage sequence identity of the templates in the threading aligned region with the query sequence

bIden2 is the percentage sequence identity of the whole template chains with query sequence 

cCov-Represents the coverage of the threading alignment and is equal to the number of aligned residues divided by the length of the query protein

dNorm. Z-score is the normalized Z-score of the threading alignments. Alignment with a normalized Z-score>1 means a good alignment and vice versa.

eAminomethyltransferase

fDmdA DMSP-dependent demethylase

gGlycine cleavage system T protein

hDimethylglycine dehydrogenase complexed with tetrahydrofolate 

iDimethylglycine oxidase

Predicted model: C-sore= 1.69; TM-score=0.95 dev= 0.05

| Rank | Class | Gene name | Organism | PDB ID | TM-scorea | RMSDb | IDENc | Covd |
| --- | --- | --- | --- | --- | --- | --- | --- | --- |
| 1 | AMT | dmdA | Pelagibacter ubique (HTCC1062) | 3tfhA | 0.981 | 0.64 | 0.367 | 0.989 |
| 2 | Oxidoreductase | dmgdH | Rattus norvegicus | 4p9sA2 | 0.931 | 1.65 | 0.209 | 0.976 |
| 3 | Oxidoreductase | dmg | Arthrobacter globiformis | 1pj6A | 0.912 | 2.10 | 0.259 | 0.976 |
| 4 | AMT | gcvT | Pyrococcus horikoshii ATCC700860 | 1v5vA | 0.906 | 1.94 | 0.226 | 0.965 |
| 5 | Oxidoreductase | soxAe | Stenotrophomonas maltophilia | 2gagA | 0.894 | 2.24 | 0.217 | 0.965 |
| 6 | Oxidoreductase | soxA | Corynebacterium sp. | 1vrqA | 0.894 | 2.22 | 0.228 | 0.965 |
| 7 | AMT | gcvT | Thermotoga maritima | 1wooA | 0.894 | 1.69 | 0.251 | 0.941 |
| 8 | AMT | AMT | Homo sapiens | 1wsrA | 0.888 | 1.98 | 0.232 | 0.949 |
| 9 | AMT | gcvT | Bartonella henselae | 3girA | 0.880 | 1.87 | 0.210 | 0.935 |
| 10 | AMT | gcvT | Bacillus subtilis | 1yx2B | 0.875 | 2.09 | 0.243 | 0.941 |

abIt is a standard for measuring structural similarity between two structures

cIt is the percentage sequence identity in the structurally aligned region 

dIt represents the coverage of the alignment by TM-align and is equal to the number of structurally aligned residues divided by length of the query protein

eHeterotetrameric sarcosine oxidase alpha-subunit

Table 8. Up: Top ten analogs identified by LOMETS for threading alignments. Case of AGI71500. In the middle: Model predicted by I-TASSER. Down: Top ten identified structural analogs in PDB by TM-align

| Rank | Class | Gene name | Organism | PDB ID | Iden1 (%)a | Iden2 (%)b | Covc | Norm. Z-scored |
| --- | --- | --- | --- | --- | --- | --- | --- | --- |
| 1 | AMTe | dmdAf | Pelagibacter ubique (HTCC1062) | 3tfhA | 36 | 35 | 0.96 | 4.55 |
| 2 | AMT | dmdA | Pelagibacter ubique (HTCC1062) | 3tfhA | 36 | 35 | 0.96 | 5.33 |
| 3 | AMT | dmdA | Pelagibacter ubique (HTCC1062) | 3tfhA | 36 | 35 | 0.96 | 5.44 |
| 4 | Oxidoreductase | dmgi | Rattus norvegicus | 4p9sA | 22 | 27 | 0.97 | 3.28 |
| 5 | Oxidoreductase | dmgdHh | Rattus norvegicus | 4p9sA | 36 | 35 | 0.96 | 2.41 |
| 6 | AMT | dmdA | Pelagibacter ubique (HTCC1062) | 3tfhA | 36 | 35 | 0.96 | 5.66 |
| 7 | Oxidoreductase | dmgdH | Rattus norvegicus | 4p9s | 22 | 29 | 0.98 | 3.64 |
| 8 | AMT | gcvTg | Bacillus subtilis | 1yx2A | 22 | 29 | 0.92 | 6.64 |
| 9 | AMT | dmdA | Pelagibacter ubique (HTCC1062) | 3tfhA | 36 | 35 | 0.95 | 7.57 |
| 10 | AMT | dmdA | Pelagibacter ubique (HTCC1062) | 3tfhA | 36 | 35 | 0.96 | 6.53 |

aIden1 is the percentage sequence identity of the templates in the threading aligned region with the query sequence

bIden2 is the percentage sequence identity of the whole template chains with query sequence 

cCov-Represents the coverage of the threading alignment and is equal to the number of aligned residues divided by the length of the query protein

dNorm. Z-score is the normalized Z-score of the threading alignments. Alignment with a normalized Z-score>1 means a good alignment and vice versa.

eAminomethyltransferase

fDmdA DMSP-dependent demethylase

gGlycine cleavage system T protein

hDimethylglycine dehydrogenase complexed with tetrahydrofolate 

iDimethylglycine oxidase

Predicted model: C-sore= 1.47; TM-score=0.92 dev= 0.06

| Rank | Class | Gene name | Organism | PDB ID | TM-scorea | RMSDb | IDENc | Covd |
| --- | --- | --- | --- | --- | --- | --- | --- | --- |
| 1 | AMT | dmdA | Pelagibacter ubique (HTCC1062) | 3tfhA | 0.949 | 0.81 | 0.360 | 0.961 |
| 2 | Oxidoreductase | dmgdH | Rattus norvegicus | 4p9sA | 0.937 | 1.74 | 0.228 | 0.982 |
| 3 | Oxidoreductase | dmg | Arthrobacter globiformis | 1pj6A | 0.907 | 2.34 | 0.264 | 0.977 |
| 4 | AMT | gcvT | Pyrococcus horikoshii ATCC700860 | 1v5vA | 0.886 | 2.09 | 0.245 | 0.948 |
| 5 | Oxidoreductase | soxAe | Stenotrophomonas maltophilia | 2gagA | 0.882 | 2.38 | 0.212 | 0.956 |
| 6 | Oxidoreductase | soxA | Corynebacterium sp. | 1vrqA | 0.881 | 2.37 | 0.231 | 0.956 |
| 7 | AMT | gcvT | Thermotoga maritima | 1wooA | 0.877 | 1.75 | 0.279 | 0.924 |
| 8 | AMT | AMT | Homo sapiens | 1wsrA | 0.874 | 2.05 | 0.256 | 0.935 |
| 9 | AMT | gcvT | Bartonella henselae | 3girA | 0.865 | 1.91 | 0.235 | 0.919 |
| 10 | AMT | gcvT | Bacillus subtilis | 1yx2B | 0.853 | 2.06 | 0.302 | 0.914 |

abIt is a standard for measuring structural similarity between two structures

cIt is the percentage sequence identity in the structurally aligned region 

dIt represents the coverage of the alignment by TM-align and is equal to the number of structurally aligned residues divided by length of the query protein

eHeterotetrameric sarcosine oxidase alpha-subunit

Table 9. Up: Top ten analogs identified by LOMETS for threading alignments. Case of WP_067545452. In the middle: Model predicted by I-TASSER. Down: Top ten identified structural analogs in PDB by TM-align

| Rank | Class | Gene name | Organism | PDB ID | Iden1 (%)a | Iden2 (%)b | Covc | Norm. Z-scored |
| --- | --- | --- | --- | --- | --- | --- | --- | --- |
| 1 | AMTe | dmdAf | Pelagibacter ubique (HTCC1062) | 3tfhA | 36 | 35 | 0.97 | 4.66 |
| 2 | AMT | dmdA | Pelagibacter ubique (HTCC1062) | 3tfhA | 36 | 35 | 0.97 | 5.36 |
| 3 | AMT | dmdA | Pelagibacter ubique (HTCC1062) | 3tfhA | 35 | 35 | 0.97 | 5.56 |
| 4 | Oxidoreductase | dmgi | Rattus norvegicus | 4p9s | 24 | 33 | 0.97 | 3.26 |
| 5 | Oxidoreductase | dmgdHh | Rattus norvegicus | 4p9sA | 21 | 26 | 0.98 | 2.40 |
| 6 | AMT | dmdA | Pelagibacter ubique (HTCC1062) | 3tfhA | 35 | 35 | 0.97 | 5.67 |
| 7 | Oxidoreductase | dmgdH | Rattus norvegicus | 4p9s | 22 | 33 | 0.97 | 3.61 |
| 8 | AMT | gcvTg | Bacillus subtilis | 1yx2A | 28 | 29 | 0.92 | 6.72 |
| 9 | AMT | dmdA | Pelagibacter ubique (HTCC1062) | 3tfhA | 36 | 35 | 0.97 | 7.48 |
| 10 | AMT | dmdA | Pelagibacter ubique (HTCC1062) | 3tfhA | 36 | 35 | 0.97 | 6.67 |

aIden1 is the percentage sequence identity of the templates in the threading aligned region with the query sequence

bIden2 is the percentage sequence identity of the whole template chains with query sequence 

cCov-Represents the coverage of the threading alignment and is equal to the number of aligned residues divided by the length of the query protein

dNorm. Z-score is the normalized Z-score of the threading alignments. Alignment with a normalized Z-score>1 means a good alignment and vice versa.

eAminomethyltransferase

fDmdA DMSP-dependent demethylase

gGlycine cleavage system T protein

hDimethylglycine dehydrogenase complexed with tetrahydrofolate 

iDimethylglycine oxidase

Predicted model: C-sore= 1.59; TM-score=0.94 dev= 0.05

| Rank | Class | Gene name | Organism | PDB ID | TM-scorea | RMSDb | IDENc | Covd |
| --- | --- | --- | --- | --- | --- | --- | --- | --- |
| 1 | AMT | dmdA | Pelagibacter ubique (HTCC1062) | 3tfhA | 0.961 | 0.85 | 0.355 | 0.974 |
| 2 | Oxidoreductase | dmgdH | Rattus norvegicus | 4p9sA | 0.942 | 1.53 | 0.226 | 0.982 |
| 3 | Oxidoreductase | dmg | Arthrobacter globiformis | 1pj6A | 0.916 | 2.11 | 0.264 | 0.979 |
| 4 | AMT | gcvT | Pyrococcus horikoshii ATCC700860 | 1v5vA | 0.898 | 2.07 | 0.266 | 0.960 |
| 5 | Oxidoreductase | soxAe | Stenotrophomonas maltophilia | 2gagA | 0.895 | 2.35 | 0.233 | 0.968 |
| 6 | Oxidoreductase | soxA | Corynebacterium sp. | 1vrqA | 0.894 | 2.27 | 0.231 | 0.966 |
| 7 | AMT | gcvT | Thermotoga maritima | 1wooA | 0.892 | 1.67 | 0.268 | 0.937 |
| 8 | AMT | AMT | Homo sapiens | 1wsrA | 0.887 | 2.02 | 0.234 | 0.947 |
| 9 | AMT | gcvT | Bartonella henselae | 3girA | 0.877 | 1.89 | 0.215 | 0.931 |
| 10 | AMT | gcvT | Bacillus subtilis | 1yx2B | 0.864 | 2.03 | 0.282 | 0.926 |

abIt is a standard for measuring structural similarity between two structures

cIt is the percentage sequence identity in the structurally aligned region 

dIt represents the coverage of the alignment by TM-align and is equal to the number of structurally aligned residues divided by length of the query protein

eHeterotetrameric sarcosine oxidase alpha-subunit

Table 10. Up: Top ten analogs identified by LOMETS for threading alignments. Case of ABF63906.1. In the middle: Model predicted by I-TASSER. Down: Top ten identified structural analogs in PDB by TM-align

| Rank | Class | Gene name | Organism | PDB ID | Iden1 (%)a | Iden2 (%)b | Covc | Norm. Z-scored |
| --- | --- | --- | --- | --- | --- | --- | --- | --- |
| 1 | AMTe | dmdAf | Pelagibacter ubique (HTCC1062) | 3tfhA | 37 | 35 | 0.97 | 4.67 |
| 2 | AMT | dmdA | Pelagibacter ubique (HTCC1062) | 3tfhA | 36 | 35 | 0.97 | 5.39 |
| 3 | AMT | dmdA | Pelagibacter ubique (HTCC1062) | 3tfhA | 36 | 35 | 0.97 | 5.50 |
| 4 | Oxidoreductase | dmgi | Rattus norvegicus | 4p9s | 25 | 34 | 0.97 | 3.27 |
| 5 | Oxidoreductase | dmgdHh | Rattus norvegicus | 4p9sA | 22 | 27 | 0.98 | 2.41 |
| 6 | AMT | dmdA | Pelagibacter ubique (HTCC1062) | 3tfhA | 36 | 35 | 0.96 | 5.62 |
| 7 | Oxidoreductase | dmg | Arthrobacter globiformis | 1pj6 | 25 | 32 | 0.97 | 3.62 |
| 8 | AMT | gcvTg | Bacillus subtilis | 1yx2A | 29 | 29 | 0.93 | 6.64 |
| 9 | AMT | dmdA | Pelagibacter ubique (HTCC1062) | 3tfhA | 37 | 35 | 0.97 | 7.50 |
| 10 | AMT | dmdA | Pelagibacter ubique (HTCC1062) | 3tfhA | 37 | 35 | 0.97 | 6.64 |

aIden1 is the percentage sequence identity of the templates in the threading aligned region with the query sequence

bIden2 is the percentage sequence identity of the whole template chains with query sequence 

cCov-Represents the coverage of the threading alignment and is equal to the number of aligned residues divided by the length of the query protein

dNorm. Z-score is the normalized Z-score of the threading alignments. Alignment with a normalized Z-score>1 means a good alignment and vice versa.

eAminomethyltransferase

fDmdA DMSP-dependent demethylase

gGlycine cleavage system T protein

hDimethylglycine dehydrogenase complexed with tetrahydrofolate 

iDimethylglycine oxidase

Predicted model: C-sore= 1.53; TM-score=0.93 dev= 0.06

| Rank | Class | Gene name | Organism | PDB ID | TM-scorea | RMSDb | IDENc | Covd |
| --- | --- | --- | --- | --- | --- | --- | --- | --- |
| 1 | AMT | dmdA | Pelagibacter ubique (HTCC1062) | 3tfhA | 0.960 | 0.68 | 0.361 | 0.968 |
| 2 | Oxidoreductase | dmgdH | Rattus norvegicus | 4p9sA | 0.935 | 1.72 | 0.244 | 0.982 |
| 3 | Oxidoreductase | dmg | Arthrobacter globiformis | 1pj6A | 0.916 | 2.14 | 0.260 | 0.982 |
| 4 | AMT | gcvT | Pyrococcus horikoshii ATCC700860 | 1v5vA | 0.895 | 2.03 | 0.267 | 0.955 |
| 5 | Oxidoreductase | soxAe | Stenotrophomonas maltophilia | 2gagA | 0.891 | 2.37 | 0.235 | 0.966 |
| 6 | Oxidoreductase | soxA | Corynebacterium sp. | 1vrqA | 0.889 | 2.40 | 0.233 | 0.966 |
| 7 | AMT | gcvT | Thermotoga maritima | 1wooA | 0.886 | 1.75 | 0.268 | 0.934 |
| 8 | AMT | AMT | Homo sapiens | 1wsrA | 0.881 | 2.03 | 0.254 | 0.942 |
| 9 | AMT | gcvT | Bartonella henselae | 3girA | 0.873 | 1.92 | 0.235 | 0.929 |
| 10 | AMT | gcvT | Bacillus subtilis | 1yx2B | 0.861 | 2.06 | 0.288 | 0.924 |

abIt is a standard for measuring structural similarity between two structures

cIt is the percentage sequence identity in the structurally aligned region 

dIt represents the coverage of the alignment by TM-align and is equal to the number of structurally aligned residues divided by length of the query protein

eHeterotetrameric sarcosine oxidase alpha-subunit

Table 11. Up: Top ten analogs identified by LOMETS for threading alignments. Case of AGI71303.1. In the middle: Model predicted by I-TASSER. Down: Top ten identified structural analogs in PDB by TM-align

| Rank | Class | Gene name | Organism | PDB ID | Iden1 (%)a | Iden2 (%)b | Covc | Norm. Z-scored |
| --- | --- | --- | --- | --- | --- | --- | --- | --- |
| 1 | AMTe | dmdAf | Pelagibacter ubique (HTCC1062) | 3tfhA | 36 | 36 | 0.97 | 4.74 |
| 2 | AMT | dmdA | Pelagibacter ubique (HTCC1062) | 3tfhA | 36 | 36 | 0.97 | 5.39 |
| 3 | AMT | dmdA | Pelagibacter ubique (HTCC1062) | 3tfhA | 36 | 36 | 0.97 | 5.60 |
| 4 | Oxidoreductase | dmgi | Rattus norvegicus | 4p9s | 25 | 33 | 0.97 | 3.27 |
| 5 | Oxidoreductase | dmgdHh | Rattus norvegicus | 4p9sA | 22 | 26 | 0.97 | 2.39 |
| 6 | AMT | dmdA | Pelagibacter ubique (HTCC1062) | 3tfhA | 36 | 36 | 0.97 | 5.72 |
| 7 | Oxidoreductase | dmg | Arthrobacter globiformis | 1pj6 | 26 | 32 | 0.97 | 3.62 |
| 8 | AMT | gcvTg | Bacillus subtilis | 1yx2A | 29 | 29 | 0.92 | 6.58 |
| 9 | AMT | dmdA | Pelagibacter ubique (HTCC1062) | 3tfhA | 36 | 36 | 0.97 | 7.67 |
| 10 | AMT | dmdA | Pelagibacter ubique (HTCC1062) | 3tfhA | 36 | 36 | 0.97 | 6.70 |

aIden1 is the percentage sequence identity of the templates in the threading aligned region with the query sequence

bIden2 is the percentage sequence identity of the whole template chains with query sequence 

cCov-Represents the coverage of the threading alignment and is equal to the number of aligned residues divided by the length of the query protein

dNorm. Z-score is the normalized Z-score of the threading alignments. Alignment with a normalized Z-score>1 means a good alignment and vice versa.

eAminomethyltransferase

fDmdA DMSP-dependent demethylase

gGlycine cleavage system T protein

hDimethylglycine dehydrogenase complexed with tetrahydrofolate 

iDimethylglycine oxidase

Predicted model: C-sore= 1.65; TM-score=0.95 dev= 0.05

| Rank | Class | Gene name | Organism | PDB ID | TM-scorea | RMSDb | IDENc | Covd |
| --- | --- | --- | --- | --- | --- | --- | --- | --- |
| 1 | AMT | dmdA | Pelagibacter ubique (HTCC1062) | 3tfhA | 0.960 | 0.68 | 0.363 | 0.971 |
| 2 | Oxidoreductase | dmgdH | Rattus norvegicus | 4p9sA | 0.931 | 1.84 | 0.239 | 0.982 |
| 3 | Oxidoreductase | dmg | Arthrobacter globiformis | 1pj6A | 0.916 | 2.220 | 0.260 | 0.982 |
| 4 | Oxidoreductase | soxAe | Stenotrophomonas maltophilia | 2gagA | 0.899 | 2.31 | 0.229 | 0.971 |
| 5 | AMT | gcvT | Pyrococcus horikoshii ATCC700860 | 1v5vA | 0.896 | 2.09 | 0.277 | 0.958 |
| 6 | Oxidoreductase | soxA | Corynebacterium sp. | 1vrqA | 0.896 | 2.28 | 0.230 | 0.968 |
| 7 | AMT | gcvT | Thermotoga maritima | 1wooA | 0.888 | 1.68 | 0.268 | 0.932 |
| 8 | AMT | AMT | Homo sapiens | 1wsrA | 0.883 | 2.05 | 0.240 | 0.945 |
| 9 | AMT | gcvT | Bartonella henselae | 3girA | 0.874 | 1.89 | 0.224 | 0.929 |
| 10 | AMT | gcvT | Bacillus subtilis | 1yx2B | 0.861 | 2.07 | 0.291 | 0.924 |

abIt is a standard for measuring structural similarity between two structures

cIt is the percentage sequence identity in the structurally aligned region 

dIt represents the coverage of the alignment by TM-align and is equal to the number of structurally aligned residues divided by length of the query protein

eHeterotetrameric sarcosine oxidase alpha-subunit

Table 12. Up: Top ten analogs identified by LOMETS for threading alignments. Case of ADE39159.1. In the middle: Model predicted by I-TASSER. Down: Top ten identified structural analogs in PDB by TM-align

| Rank | Class | Gene name | Organism | PDB ID | Iden1 (%)a | Iden2 (%)b | Covc | Norm. Z-scored |
| --- | --- | --- | --- | --- | --- | --- | --- | --- |
| 1 | AMTe | dmdAf | Pelagibacter ubique (HTCC1062) | 3tfhA | 34 | 34 | 0.96 | 4.55 |
| 2 | AMT | dmdA | Pelagibacter ubique (HTCC1062) | 3tfhA | 34 | 34 | 0.96 | 5.36 |
| 3 | AMT | dmdA | Pelagibacter ubique (HTCC1062) | 3tfhA | 34 | 34 | 0.96 | 5.57 |
| 4 | Oxidoreductase | dmgi | Arthrobacter globiformis | 1pj6 | 26 | 32 | 0.97 | 3.24 |
| 5 | Oxidoreductase | dmgdHh | Rattus norvegicus | 4p9sA | 22 | 26 | 0.97 | 2.41 |
| 6 | AMT | dmdA | Pelagibacter ubique (HTCC1062) | 3tfhA | 34 | 34 | 0.95 | 5.56 |
| 7 | Oxidoreductase | dmg | Arthrobacter globiformis | 1pj6 | 27 | 32 | 0.96 | 3.60 |
| 8 | AMT | gcvTg | Pyrococcus horikoshii | 1v5vA | 26 | 29 | 0.95 | 6.32 |
| 9 | AMT | dmdA | Pelagibacter ubique (HTCC1062) | 3tfhA | 34 | 34 | 0.96 | 7.48 |
| 10 | AMT | dmdA | Pelagibacter ubique (HTCC1062) | 3tfhA | 34 | 34 | 0.96 | 6.56 |

aIden1 is the percentage sequence identity of the templates in the threading aligned region with the query sequence

bIden2 is the percentage sequence identity of the whole template chains with query sequence 

cCov-Represents the coverage of the threading alignment and is equal to the number of aligned residues divided by the length of the query protein

dNorm. Z-score is the normalized Z-score of the threading alignments. Alignment with a normalized Z-score>1 means a good alignment and vice versa.

eAminomethyltransferase

fDmdA DMSP-dependent demethylase

gGlycine cleavage system T protein

hDimethylglycine dehydrogenase complexed with tetrahydrofolate 

iDimethylglycine oxidase

Predicted model: C-sore= 1.50; TM-score=0.92 dev= 0.06

| Rank | Class | Gene name | Organism | PDB ID | TM-scorea | RMSDb | IDENc | Covd |
| --- | --- | --- | --- | --- | --- | --- | --- | --- |
| 1 | AMT | dmdA | Pelagibacter ubique (HTCC1062) | 3tfhA | 0.950 | 0.79 | 0.341 | 0.961 |
| 2 | Oxidoreductase | dmgdH | Rattus norvegicus | 4p9sA | 0.933 | 1.77 | 0.231 | 0.982 |
| 3 | Oxidoreductase | dmg | Arthrobacter globiformis | 1pj6A | 0.928 | 1.89 | 0.249 | 0.982 |
| 4 | Oxidoreductase | soxAe | Stenotrophomonas maltophilia | 2gagA | 0.888 | 2.23 | 0.206 | 0.958 |
| 5 | AMT | gcvT | Pyrococcus horikoshii ATCC700860 | 1v5vA | 0.887 | 2.08 | 0.261 | 0.948 |
| 6 | Oxidoreductase | soxA | Corynebacterium sp. | 1vrqA | 0.886 | 2.22 | 0.222 | 0.956 |
| 7 | AMT | gcvT | Thermotoga maritima | 1wooA | 0.880 | 1.70 | 0.265 | 0.924 |
| 8 | AMT | AMT | Homo sapiens | 1wsrA | 0.877 | 1.99 | 0.248 | 0.935 |
| 9 | AMT | gcvT | Bartonella henselae | 3girA | 0.868 | 1.84 | 0.218 | 0.919 |
| 10 | AMT | gcvT | Bacillus subtilis | 1yx2B | 0.857 | 1.97 | 0.279 | 0.914 |

abIt is a standard for measuring structural similarity between two structures

cIt is the percentage sequence identity in the structurally aligned region 

dIt represents the coverage of the alignment by TM-align and is equal to the number of structurally aligned residues divided by length of the query protein

eHeterotetrameric sarcosine oxidase alpha-subunit

Table 13. Up: Top ten analogs identified by LOMETS for threading alignments. Case of AFS47213.1. In the middle: Model predicted by I-TASSER. Down: Top ten identified structural analogs in PDB by TM-align

| Rank | Class | Gene name | Organism | PDB ID | Iden1 (%)a | Iden2 (%)b | Covc | Norm. Z-scored |
| --- | --- | --- | --- | --- | --- | --- | --- | --- |
| 1 | AMTe | dmdAf | Pelagibacter ubique (HTCC1062) | 3tfhA | 38 | 37 | 0.97 | 4.75 |
| 2 | AMT | dmdA | Pelagibacter ubique (HTCC1062) | 3tfhA | 37 | 37 | 0.97 | 5.39 |
| 3 | AMT | dmdA | Pelagibacter ubique (HTCC1062) | 3tfhA | 38 | 37 | 0.97 | 5.54 |
| 4 | AMT | dmdA | Pelagibacter ubique (HTCC1062) | 3tfh | 38 | 37 | 0.97 | 3.26 |
| 5 | Oxidoreductase | dmgdHh | Rattus norvegicus | 4p9sA | 22 | 25 | 0.98 | 2.40 |
| 6 | AMT | dmdA | Pelagibacter ubique (HTCC1062) | 3tfhA | 38 | 37 | 0.97 | 5.82 |
| 7 | Oxidoreductase | dmgdH | Rattus norvegicus | 4p9s | 23 | 32 | 0.97 | 3.61 |
| 8 | AMT | gcvTg | Bacillus subtilis | 1yx2A | 30 | 31 | 0.93 | 6.83 |
| 9 | AMT | dmdA | Pelagibacter ubique (HTCC1062) | 3tfhA | 38 | 37 | 0.97 | 7.70 |
| 10 | AMT | dmdA | Pelagibacter ubique (HTCC1062) | 3tfhA | 38 | 37 | 0.97 | 6.75 |

aIden1 is the percentage sequence identity of the templates in the threading aligned region with the query sequence

bIden2 is the percentage sequence identity of the whole template chains with query sequence 

cCov-Represents the coverage of the threading alignment and is equal to the number of aligned residues divided by the length of the query protein

dNorm. Z-score is the normalized Z-score of the threading alignments. Alignment with a normalized Z-score>1 means a good alignment and vice versa.

eAminomethyltransferase

fDmdA DMSP-dependent demethylase

gGlycine cleavage system T protein

hDimethylglycine dehydrogenase complexed with tetrahydrofolate 

Predicted model: C-sore= 1.66; TM-score=0.95 dev= 0.05

| Rank | Class | Gene name | Organism | PDB ID | TM-scorea | RMSDb | IDENc | Covd |
| --- | --- | --- | --- | --- | --- | --- | --- | --- |
| 1 | AMT | dmdA | Pelagibacter ubique (HTCC1062) | 3tfhA | 0.966 | 0.67 | 0.374 | 0.974 |
| 2 | Oxidoreductase | dmgdH | Rattus norvegicus | 4p9sA | 0.928 | 1.83 | 0.231 | 0.982 |
| 3 | Oxidoreductase | dmge | Arthrobacter globiformis | 1pj6A | 0.905 | 2.34 | 0.247 | 0.982 |
| 4 | AMT | gcvT | Pyrococcus horikoshii ATCC700860 | 1v5vA | 0.891 | 2.11 | 0.275 | 0.958 |
| 5 | Oxidoreductase | soxAf | Stenotrophomonas maltophilia | 2gagA | 0.888 | 2.52 | 0.241 | 0.971 |
| 6 | Oxidoreductase | soxA | Corynebacterium sp. | 1vrqA | 0.886 | 2.44 | 0.228 | 0.968 |
| 7 | AMT | gcvT | Thermotoga maritima | 1wooA | 0.884 | 1.85 | 0.273 | 0.937 |
| 8 | AMT | AMT | Homo sapiens | 1wsrA | 0.876 | 2.19 | 0.248 | 0.947 |
| 9 | AMT | gcvT | Bartonella henselae | 3girA | 0.873 | 1.95 | 0.221 | 0.931 |
| 10 | AMT | gcvT | Bacillus subtilis | 1yx2B | 0.858 | 2.15 | 0.308 | 0.926 |

abIt is a standard for measuring structural similarity between two structures

cIt is the percentage sequence identity in the structurally aligned region 

dIt represents the coverage of the alignment by TM-align and is equal to the number of structurally aligned residues divided by length of the query protein

eDimethylglycine oxidase

fHeterotetrameric sarcosine oxidase alpha-subunit

Table 14. Up: Top ten analogs identified by LOMETS for threading alignments. Case of AFS48354. In the middle: Model predicted by I-TASSER. Down: Top ten identified structural analogs in PDB by TM-align

| Rank | Class | Gene name | Organism | PDB ID | Iden1 (%)a | Iden2 (%)b | Covc | Norm. Z-scored |
| --- | --- | --- | --- | --- | --- | --- | --- | --- |
| 1 | AMTe | dmdAf | Pelagibacter ubique (HTCC1062) | 3tfhA | 40 | 40 | 0.97 | 4.69 |
| 2 | AMT | dmdA | Pelagibacter ubique (HTCC1062) | 3tfhA | 40 | 40 | 0.97 | 5.33 |
| 3 | AMT | dmdA | Pelagibacter ubique (HTCC1062) | 3tfhA | 39 | 40 | 0.97 | 5.38 |
| 4 | Oxidoreductase | dmgdHh | Rattus norvegicus | 4p9s | 24 | 33 | 0.97 | 3.27 |
| 5 | Oxidoreductase | dmgdH | Rattus norvegicus | 4p9sA | 23 | 27 | 0.98 | 2.40 |
| 6 | AMT | dmdA | Pelagibacter ubique (HTCC1062) | 3tfhA | 39 | 40 | 0.97 | 5.81 |
| 7 | Oxidoreductase | dmgdH | Rattus norvegicus | 4p9s | 24 | 33 | 0.97 | 3.61 |
| 8 | AMT | gcvTg | Bacillus subtilis | 1yx2A | 29 | 29 | 0.93 | 6.58 |
| 9 | AMT | dmdA | Pelagibacter ubique (HTCC1062) | 3tfhA | 40 | 40 | 0.97 | 7.47 |
| 10 | AMT | dmdA | Pelagibacter ubique (HTCC1062) | 3tfhA | 40 | 40 | 0.97 | 6.62 |

aIden1 is the percentage sequence identity of the templates in the threading aligned region with the query sequence

bIden2 is the percentage sequence identity of the whole template chains with query sequence 

cCov-Represents the coverage of the threading alignment and is equal to the number of aligned residues divided by the length of the query protein

dNorm. Z-score is the normalized Z-score of the threading alignments. Alignment with a normalized Z-score>1 means a good alignment and vice versa.

eAminomethyltransferase

fDmdA DMSP-dependent demethylase

gGlycine cleavage system T protein

hDimethylglycine dehydrogenase complexed with tetrahydrofolate 

Predicted model: C-sore= 1.60; TM-score=0.94 dev= 0.05

| Rank | Class | Gene name | Organism | PDB ID | TM-scorea | RMSDb | IDENc | Covd |
| --- | --- | --- | --- | --- | --- | --- | --- | --- |
| 1 | AMT | dmdA | Pelagibacter ubique (HTCC1062) | 3tfhA | 0.963 | 0.77 | 0.398 | 0.974 |
| 2 | Oxidoreductase | dmgdH | Rattus norvegicus | 4p9sA | 0.938 | 1.63 | 0.239 | 0.982 |
| 3 | Oxidoreductase | dmge | Arthrobacter globiformis | 1pj6A | 0.913 | 2.16 | 0.261 | 0.979 |
| 4 | AMT | gcvT | Pyrococcus horikoshii ATCC700860 | 1v5vA | 0.897 | 2.09 | 0.258 | 0.960 |
| 5 | Oxidoreductase | soxAf | Stenotrophomonas maltophilia | 2gagA | 0.890 | 2.43 | 0.231 | 0.968 |
| 6 | Oxidoreductase | soxA | Corynebacterium sp. | 1vrqA | 0.888 | 2.35 | 0.228 | 0.966 |
| 7 | AMT | gcvT | Thermotoga maritima | 1wooA | 0.888 | 1.76 | 0.282 | 0.937 |
| 8 | AMT | AMT | Homo sapiens | 1wsrA | 0.885 | 2.05 | 0.234 | 0.947 |
| 9 | AMT | gcvT | Bartonella henselae | 3girA | 0.875 | 1.92 | 0.221 | 0.931 |
| 10 | AMT | gcvT | Bacillus subtilis | 1yx2B | 0.862 | 2.07 | 0.302 | 0.926 |

abIt is a standard for measuring structural similarity between two structures

cIt is the percentage sequence identity in the structurally aligned region 

dIt represents the coverage of the alignment by TM-align and is equal to the number of structurally aligned residues divided by length of the query protein

eDimethylglycine oxidase

fHeterotetrameric sarcosine oxidase alpha-subunit

Table 15. Up: Top ten analogs identified by LOMETS for threading alignments. Case of WP_065353845. In the middle: Model predicted by I-TASSER. Down: Top ten identified structural analogs in PDB by TM-align

| Rank | Class | Gene name | Organism | PDB ID | Iden1 (%)a | Iden2 (%)b | Covc | Norm. Z-scored |
| --- | --- | --- | --- | --- | --- | --- | --- | --- |
| 1 | AMTe | dmdAf | Pelagibacter ubique (HTCC1062) | 3tfhA | 37 | 38 | 0.97 | 4.72 |
| 2 | AMT | dmdA | Pelagibacter ubique (HTCC1062) | 3tfhA | 36 | 38 | 0.97 | 5.36 |
| 3 | AMT | dmdA | Pelagibacter ubique (HTCC1062) | 3tfhA | 36 | 38 | 0.97 | 5.59 |
| 4 Oxidoreductase | Oxidoreductase | dmgdH | Rattus norvegicus | 4p9s | 23 | 30 | 0.97 | 3.27 |
| 5 | Oxidoreductase | dmgdHh | Rattus norvegicus | 4p9sA | 22 | 24 | 0.98 | 2.40 |
| 6 | AMT | dmdA | Pelagibacter ubique (HTCC1062) | 3tfhA | 36 | 38 | 0.97 | 5.82 |
| 7 | Oxidoreductase | dmgi | Arthrobacter globiformis | 1pj6 | 26 | 30 | 0.97 | 3.62 |
| 8 | AMT | gcvTg | Bacillus subtilis | 1yx2A | 27 | 27 | 0.93 | 6.58 |
| 9 | AMT | dmdA | Pelagibacter ubique (HTCC1062) | 3tfhA | 37 | 38 | 0.97 | 7.59 |
| 10 | AMT | dmdA | Pelagibacter ubique (HTCC1062) | 3tfhA | 37 | 38 | 0.97 | 6.56 |

aIden1 is the percentage sequence identity of the templates in the threading aligned region with the query sequence

bIden2 is the percentage sequence identity of the whole template chains with query sequence 

cCov-Represents the coverage of the threading alignment and is equal to the number of aligned residues divided by the length of the query protein

dNorm. Z-score is the normalized Z-score of the threading alignments. Alignment with a normalized Z-score>1 means a good alignment and vice versa.

eAminomethyltransferase

fDmdA DMSP-dependent demethylase

gGlycine cleavage system T protein

hDimethylglycine dehydrogenase complexed with tetrahydrofolate 

iDimethylglycine oxidase

Predicted model: C-sore= 1.56; TM-score=0.93 dev= 0.06

| Rank | Class | Gene name | Organism | PDB ID | TM-scorea | RMSDb | IDENc | Covd |
| --- | --- | --- | --- | --- | --- | --- | --- | --- |
| 1 | AMT | dmdA | Pelagibacter ubique (HTCC1062) | 3tfhA | 0.961 | 0.74 | 0.361 | 0.971 |
| 2 | Oxidoreductase | dmgdH | Rattus norvegicus | 4p9sA | 0.939 | 1.60 | 0.226 | 0.982 |
| 3 | Oxidoreductase | dmg | Arthrobacter globiformis | 1pj6A | 0.915 | 2.12 | 0.251 | 0.979 |
| 4 | AMT | gcvT | Pyrococcus horikoshii ATCC700860 | 1v5vA | 0.898 | 2.15 | 0.238 | 0.963 |
| 5 | Oxidoreductase | soxAe | Stenotrophomonas maltophilia | 2gagA | 0.895 | 2.34 | 0.220 | 0.968 |
| 6 | Oxidoreductase | soxA | Corynebacterium sp. | 1vrqA | 0.893 | 2.27 | 0.218 | 0.966 |
| 7 | AMT | gcvT | Thermotoga maritima | 1wooA | 0.888 | 1.76 | 0.265 | 0.937 |
| 8 | AMT | AMT | Homo sapiens | 1wsrA | 0.886 | 2.05 | 0.217 | 0.947 |
| 9 | AMT | gcvT | Bartonella henselae | 3girA | 0.875 | 1.92 | 0.207 | 0.931 |
| 10 | AMT | gcvT | Bacillus subtilis | 1yx2B | 0.863 | 2.07 | 0.276 | 0.926 |

abIt is a standard for measuring structural similarity between two structures

cIt is the percentage sequence identity in the structurally aligned region 

dIt represents the coverage of the alignment by TM-align and is equal to the number of structurally aligned residues divided by length of the query protein

eHeterotetrameric sarcosine oxidase alpha-subunit

Table 16. Up: Top ten analogs identified by LOMETS for threading alignments. Case of WP_053820730.1. In the middle: Model predicted by I-TASSER. Down: Top ten identified structural analogs in PDB by TM-align

| Rank | Class | Gene name | Organism | PDB ID | Iden1 (%)a | Iden2 (%)b | Covc | Norm. Z-scored |
| --- | --- | --- | --- | --- | --- | --- | --- | --- |
| 1 | AMTe | dmdAf | Pelagibacter ubique (HTCC1062) | 3tfhA | 37 | 21 | 0.53 | 2.51 |
| 2 | Oxidoreductase | dmgdHh | Rattus norvegicus | 4pabA | 17 | 22 | 0.90 | 4.36 |
| 3 | AMT | dmdA | Pelagibacter ubique (HTCC1062) | 3tfhA | 36 | 21 | 0.54 | 4.25 |
| 4 | Oxidoreductase | dmgdH | Rattus norvegicus | 4p9sA | 24 | 17 | 0.54 | 3.30 |
| 5 | Oxidoreductase | dmg | Arthrobacter globiformis | 1pj6 | 20 | 25 | 0.94 | 2.65 |
| 6 | Oxidoreductase | dmgdH | Rattus norvegicus | 4p9sA | 17 | 22 | 0.91 | 4.79 |
| 7 | Oxidoreductase | dmgi | Arthrobacter globiformis | 1pj6 | 26 | 25 | 0.54 | 3.59 |
| 8 | AMT | gcvTg | Bacillus subtilis | 1yx2A | 27 | 15 | 0.51 | 6.47 |
| 9 | AMT | dmdA | Pelagibacter ubique (HTCC1062) | 3tfhA | 37 | 21 | 0.53 | 2.78 |
| 10 | AMT | dmdA | Pelagibacter ubique (HTCC1062) | 3tfhA | 36 | 21 | 0.54 | 5.61 |

aIden1 is the percentage sequence identity of the templates in the threading aligned region with the query sequence

bIden2 is the percentage sequence identity of the whole template chains with query sequence 

cCov-Represents the coverage of the threading alignment and is equal to the number of aligned residues divided by the length of the query protein

dNorm. Z-score is the normalized Z-score of the threading alignments. Alignment with a normalized Z-score>1 means a good alignment and vice versa.

eAminomethyltransferase

fDmdA DMSP-dependent demethylase

gGlycine cleavage system T protein

hDimethylglycine dehydrogenase complexed with tetrahydrofolate 

iDimethylglycine oxidase

Predicted model: C-sore= 0.34; TM-score=0.67 dev= 0.13

| Rank | Class | Gene name | Organism | PDB ID | TM-scorea | RMSDb | IDENc | Covd |
| --- | --- | --- | --- | --- | --- | --- | --- | --- |
| 1 | Oxidoreductase | dmgdH | Rattus norvegicus | 4p9sA | 0.874 | 2.56 | 0.157 | 0.921 |
| 2 | Oxidoreductase | dmg | Arthrobacter globiformis | 1pj6A | 0.867 | 2.72 | 0.184 | 0.923 |
| 3 | Oxidoreductase | soxAe | Stenotrophomonas maltophilia | 2gagA | 0.536 | 3.70 | 0.146 | 0.590 |
| 4 | Oxidoreductase | soxA | Corynebacterium sp. | 1vrqA | 0.534 | 3.65 | 0.141 | 0.587 |
| 5 | AMT | dmdA | Pelagibacter ubique (HTCC1062) | 3tfhA | 0.520 | 1.57 | 0.360 | 0.534 |
| 6 | AMT | gcvT | Pyrococcus horikoshii | 1v5vA | 0.510 | 2.05 | 0.238 | 0.531 |
| 7 | AMT | gcvT | Thermotoga maritima | 1wooA | 0.506 | 1.47 | 0.272 | 0.518 |
| 8 | AMT | AMT | Homo sapiens | 1wsrA | 0.503 | 1.73 | 0.216 | 0.520 |
| 9 | AMT | gcvT | Bartonella henselae | 3girA | 0.495 | 1.88 | 0.204 | 0.514 |
| 10 | AMT | gcvT | Bacillus subtilis | 1yx2B | 0.494 | 1.67 | 0.274 | 0.509 |

abIt is a standard for measuring structural similarity between two structures

cIt is the percentage sequence identity in the structurally aligned region 

dIt represents the coverage of the alignment by TM-align and is equal to the number of structurally aligned residues divided by length of the query protein

eHeterotetrameric sarcosine oxidase alpha-subunit

Table 17. Up: Top ten analogs identified by LOMETS for threading alignments. Case of CCC39909.1. In the middle: Model predicted by I-TASSER. Down: Top ten identified structural analogs in PDB by TM-align

| Rank | Class | Gene name | Organism | PDB ID | Iden1 (%)a | Iden2 (%)b | Covc | Norm. Z-scored |
| --- | --- | --- | --- | --- | --- | --- | --- | --- |
| 1 | AMTe | gcvTg | Thermotoga maritima | 1wopA | 26 | 25 | 0.78 | 3.50 |
| 2 | AMT | gcvT | Pyrococcus horikoshii | 1v5vA | 25 | 24 | 0.80 | 4.91 |
| 3 | AMT | gcvT | Thermotoga maritima | 1worA | 27 | 25 | 0.78 | 4.66 |
| 4 | Oxidoreductase | dmgdHh | Rattus norvegicus | 4p9s | 17 | 19 | 0.88 | 3.44 |
| 5 | Oxidoreductase | dmgdH | Rattus norvegicus | 4p9sA | 16 | 19 | 0.83 | 2.44 |
| 6 | AMT | dmdAf | Pelagibacter ubique (HTCC1062) | 3tfhA | 25 | 22 | 0.80 | 4.87 |
| 7 | Oxidoreductase | dmgdH | Rattus norvegicus | 4p9s | 18 | 19 | 0.82 | 3.63 |
| 8 | AMT | gcvT | Bacillus subtilis | 1yx2A | 26 | 22 | 0.77 | 5.74 |
| 9 | AMT | gcvT | Thermotoga maritima | 1wopA | 26 | 25 | 0.78 | 5.21 |
| 10 | Oxidoreductase | dmgdH | Rattus norvegicus | 4p9sA | 17 | 19 | 0.84 | 5.80 |

aIden1 is the percentage sequence identity of the templates in the threading aligned region with the query sequence

bIden2 is the percentage sequence identity of the whole template chains with query sequence 

cCov-Represents the coverage of the threading alignment and is equal to the number of aligned residues divided by the length of the query protein

dNorm. Z-score is the normalized Z-score of the threading alignments. Alignment with a normalized Z-score>1 means a good alignment and vice versa.

eAminomethyltransferase

fDmdA DMSP-dependent demethylase

gGlycine cleavage system T protein

hDimethylglycine dehydrogenase complexed with tetrahydrofolate 

Predicted model: C-sore= -0.06; TM-score=0.71 dev= 0.12

| Rank | Class | Gene name | Organism | PDB ID | TM-scorea | RMSDb | IDENc | Covd |
| --- | --- | --- | --- | --- | --- | --- | --- | --- |
| 1 | Oxidoreductase | dmgdH | Rattus norvegicus | 4p9sA | 0.865 | 1.32 | 0.152 | 0.889 |
| 2 | Oxidoreductase | dmge | Arthrobacter globiformis | 1pj6A | 0.838 | 1.93 | 0.193 | 0.882 |
| 3 | Oxidoreductase | soxAf | Stenotrophomonas maltophilia | 2gagA | 0.779 | 2.65 | 0.203 | 0.841 |
| 4 | Oxidoreductase | soxA | Corynebacterium sp. | 1vrqA | 0.776 | 2.68 | 0.195 | 0.838 |
| 5 | AMT | gcvT | Pyrococcus horikoshii | 1v5vA | 0.773 | 1.66 | 0.237 | 0.801 |
| 6 | AMT | gcvT | Thermotoga maritima | 1worA | 0.763 | 1.11 | 0.261 | 0.777 |
| 7 | AMT | AMT | Homo sapiens | 1wsvB | 0.754 | 1.71 | 0.200 | 0.786 |
| 8 | AMT | dmdA | Pelagibacter ubique (HTCC1062) | 3tfiA | 0.752 | 2..02 | 0.246 | 0.799 |
| 9 | AMT | gcvT | Bartonella henselae | 3girA | 0.740 | 1.86 | 0.202 | 0.777 |
| 10 | AMT | gcvT | Escherichia coli BL21 | 3a8iA | 0.739 | 1.82 | 0.203 | 0.773 |

abIt is a standard for measuring structural similarity between two structures

cIt is the percentage sequence identity in the structurally aligned region 

dIt represents the coverage of the alignment by TM-align and is equal to the number of structurally aligned residues divided by length of the query protein

eDimethylglycine oxidase

fHeterotetrameric sarcosine oxidase alpha-subunit

Table 18. Up: Top ten analogs identified by LOMETS for threading alignments. Case of CAJ51984.2. In the middle: Model predicted by I-TASSER. Down: Top ten identified structural analogs in PDB by TM-align

| Rank | Class | Gene name | Organism | PDB ID | Iden1 (%)a | Iden2 (%)b | Covc | Norm. Z-scored |
| --- | --- | --- | --- | --- | --- | --- | --- | --- |
| 1 | AMTe | gcvTg | Thermotoga maritima | 1wopA | 26 | 25 | 0.78 | 3.57 |
| 2 | AMT | gcvT | Pyrococcus horikoshii | 1v5vA | 25 | 24 | 0.80 | 4.79 |
| 3 | AMT | gcvT | Thermotoga maritima | 1worA | 27 | 25 | 0.78 | 4.65 |
| 4 | Oxidoreductase | dmgdHh | Rattus norvegicus | 4p9s | 17 | 19 | 0.88 | 3.45 |
| 5 | Oxidoreductase | dmgdH | Rattus norvegicus | 4p9sA | 16 | 19 | 0.83 | 2.43 |
| 6 | AMT | dmdAf | Pelagibacter ubique (HTCC1062) | 3tfhA | 25 | 22 | 0.80 | 4.89 |
| 7 | Oxidoreductase | dmgdH | Rattus norvegicus | 4p9s | 17 | 19 | 0.83 | 3.62 |
| 8 | AMT | gcvT | Bacillus subtilis | 1yx2A | 26 | 22 | 0.77 | 5.74 |
| 9 | AMT | gcvT | Thermotoga maritima | 1wopA | 26 | 25 | 0.78 | 5.26 |
| 10 | AMT | dmdA | Pelagibacter ubique (HTCC1062) | 3tfhA | 27 | 22 | 0.80 | 5.83 |

aIden1 is the percentage sequence identity of the templates in the threading aligned region with the query sequence

bIden2 is the percentage sequence identity of the whole template chains with query sequence 

cCov-Represents the coverage of the threading alignment and is equal to the number of aligned residues divided by the length of the query protein

dNorm. Z-score is the normalized Z-score of the threading alignments. Alignment with a normalized Z-score>1 means a good alignment and vice versa.

eAminomethyltransferase

fDmdA DMSP-dependent demethylase

gGlycine cleavage system T protein

hDimethylglycine dehydrogenase complexed with tetrahydrofolate 

Predicted model: C-sore= 0.23; TM-score=0.68 dev= 0.12

| Rank | Class | Gene name | Organism | PDB ID | TM-scorea | RMSDb | IDENc | Covd |
| --- | --- | --- | --- | --- | --- | --- | --- | --- |
| 1 | Oxidoreductase | dmgdH | Rattus norvegicus | 4p9sA | 0.855 | 1.70 | 0.140 | 0.889 |
| 2 | Oxidoreductase | dmge | Arthrobacter globiformis | 1pj7A | 0.825 | 2.29 | 0.193 | 0.882 |
| 3 | Oxidoreductase | soxAf | Stenotrophomonas maltophilia | 2gagA | 0.781 | 2.46 | 0.198 | 0.836 |
| 4 | Oxidoreductase | soxA | Corynebacterium sp. | 1vrqA | 0.778 | 2.39 | 0.193 | 0.832 |
| 5 | AMT | gcvT | Pyrococcus horikoshii | 1v5vA | 0.774 | 1.67 | 0.240 | 0.801 |
| 6 | AMT | gcvT | Thermotoga maritima | 1worA | 0.765 | 1.01 | 0.258 | 0.777 |
| 7 | AMT | AMT | Homo sapiens | 1wsvB | 0.756 | 1.57 | 0.206 | 0.784 |
| 8 | AMT | dmdA | Pelagibacter ubique (HTCC1062) | 3tfiA | 0.752 | 2.03 | 0.238 | 0.799 |
| 9 | AMT | gcvT | Bartonella henselae | 3girA | 0.742 | 1.77 | 0.208 | 0.775 |
| 10 | AMT | gcvT | Escherichia coli BL21 | 3a8iA | 0.739 | 1.81 | 0.209 | 0.773 |

abIt is a standard for measuring structural similarity between two structures

cIt is the percentage sequence identity in the structurally aligned region 

dIt represents the coverage of the alignment by TM-align and is equal to the number of structurally aligned residues divided by length of the query protein

eDimethylglycine oxidase

fHeterotetrameric sarcosine oxidase alpha-subunit

Table 19. Up: Top ten analogs identified by LOMETS for threading alignments. Case of AEM59334.1. In the middle: Model predicted by I-TASSER. Down: Top ten identified structural analogs in PDB by TM-align

| Rank | Class | Gene name | Organism | PDB ID | Iden1 (%)a | Iden2 (%)b | Covc | Norm. Z-scored |
| --- | --- | --- | --- | --- | --- | --- | --- | --- |
| 1 | AMTe | dmdAf | Pelagibacter ubique (HTCC1062) | 3tfhA | 27 | 18 | 0.59 | 2.15 |
| 2 | AMT | gcvTg | Pyrococcus horikoshii | 1v5vA | 26 | 19 | 0.58 | 4.37 |
| 3 | AMT | gcvT | Thermotoga maritima | 1worA | 27 | 18 | 0.57 | 3.64 |
| 4 | Oxidoreductase | dmgdHh | Rattus norvegicus | 4p9s | 17 | 16 | 0.65 | 2.89 |
| 5 | Oxidoreductase | dmgdH | Rattus norvegicus | 4p9sA | 17 | 15 | 0.60 | 2.26 |
| 6 | AMT | dmdA | Pelagibacter ubique (HTCC1062) | 3tfhA | 27 | 18 | 0.59 | 3.22 |
| 7 | Oxidoreductase | dmgdH | Rattus norvegicus | 4p9s | 17 | 16 | 0.62 | 3.03 |
| 8 | AMT | gcvT | Bacillus subtilis | 1yx2A | 24 | 16 | 0.56 | 5.85 |
| 9 | AMT | dmdA | Pelagibacter ubique (HTCC1062) | 3tfhA | 27 | 18 | 0.59 | 3.32 |
| 10 | AMT | dmdA | Pelagibacter ubique (HTCC1062) | 3tfhA | 27 | 18 | 0.59 | 4.46 |

aIden1 is the percentage sequence identity of the templates in the threading aligned region with the query sequence

bIden2 is the percentage sequence identity of the whole template chains with query sequence 

cCov-Represents the coverage of the threading alignment and is equal to the number of aligned residues divided by the length of the query protein

dNorm. Z-score is the normalized Z-score of the threading alignments. Alignment with a normalized Z-score>1 means a good alignment and vice versa.

eAminomethyltransferase

fDmdA DMSP-dependent demethylase

gGlycine cleavage system T protein

hDimethylglycine dehydrogenase complexed with tetrahydrofolate 

Predicted model: C-sore=-2.53; TM-score=0.42 dev= 0.14

| Rank | Class | Gene name | Organism | PDB ID | TM-scorea | RMSDb | IDENc | Covd |
| --- | --- | --- | --- | --- | --- | --- | --- | --- |
| 1 | Oxidoreductase | dmgdH | Rattus norvegicus | 4p9sA | 0.637 | 1.14 | 0.151 | 0.647 |
| 2 | Oxidoreductase | dmge | Arthrobacter globiformis | 1pj6A | 0.613 | 1.91 | 0.204 | 0.639 |
| 3 | Oxidoreductase | soxAf | Stenotrophomonas maltophilia | 2gagA | 0.562 | 2.99 | 0.192 | 0.610 |
| 4 | AMT | gcvT | Pyrococcus horikoshii | 1v5vA | 0.562 | 2.01 | 0.249 | 0.588 |
| 5 | Oxidoreductase | soxA | Corynebacterium sp. | 1vrqA | 0.561 | 3.03 | 0.190 | 0.608 |
| 6 | AMT | dmdA | Pelagibacter ubique (HTCC1062) | 3tfiA | 0.554 | 2.33 | 0.259 | 0.589 |
| 7 | AMT | gcvT | Thermotoga maritima | 1wopA | 0.553 | 1.57 | 0.265 | 0.570 |
| 8 | AMT | AMT | Homo sapiens | 1wsvB | 0.551 | 1.76 | 0.219 | 0.571 |
| 9 | AMT | gcvT | Bacillus subtilis (168) | 1yx2B | 0.541 | 1.77 | 0.249 | 0.562 |
| 10 | AMT | gcvT | Bartonella henselae | 3girA | 0.540 | 2.00 | 0.216 | 0.565 |

abIt is a standard for measuring structural similarity between two structures

cIt is the percentage sequence identity in the structurally aligned region 

dIt represents the coverage of the alignment by TM-align and is equal to the number of structurally aligned residues divided by length of the query protein

eDimethylglycine oxidase

fHeterotetrameric sarcosine oxidase alpha-subunit

Table 20. Up: Top ten analogs identified by LOMETS for threading alignments. Case of AFS48830. In the middle: Model predicted by I-TASSER. Down: Top ten identified structural analogs in PDB by TM-align

| Rank | Class | Gene name | Organism | PDB ID | Iden1 (%)a | Iden2 (%)b | Covc | Norm. Z-scored |
| --- | --- | --- | --- | --- | --- | --- | --- | --- |
| 1 | AMTe | gcvT | Thermotoga maritima | 1wopA | 29 | 26 | 0.82 | 3.78 |
| 2 | AMT | gcvTg | Pyrococcus horikoshii | 1v5vA | 25 | 24 | 0.84 | 4.85 |
| 3 | AMT | gcvT | Thermotoga maritima | 1worA | 28 | 26 | 0.82 | 4.85 |
| 4 | Oxidoreductase | dmgdHh | Rattus norvegicus | 4p9sA | 18 | 19 | 0.86 | 3.31 |
| 5 | Oxidoreductase | dmgdH | Rattus norvegicus | 4p9sA | 19 | 19 | 0.86 | 2.40 |
| 6 | AMT | gcvT | Thermotoga maritima | 1wopA | 28 | 26 | 0.82 | 4.97 |
| 7 | Oxidoreductase | dmgdH | Rattus norvegicus | 4p9s | 18 | 20 | 0.87 | 3.67 |
| 8 | AMT | gcvT | Bacillus subtilis | 1yx2A | 25 | 22 | 0.81 | 6.32 |
| 9 | AMT | gcvT | Thermotoga maritima | 1wopA | 28 | 26 | 0.82 | 5.48 |
| 10 | AMT | dmdAf | Pelagibacter ubique (HTCC1062) | 3tfhA | 27 | 25 | 0.85 | 6.04 |

aIden1 is the percentage sequence identity of the templates in the threading aligned region with the query sequence

bIden2 is the percentage sequence identity of the whole template chains with query sequence 

cCov-Represents the coverage of the threading alignment and is equal to the number of aligned residues divided by the length of the query protein

dNorm. Z-score is the normalized Z-score of the threading alignments. Alignment with a normalized Z-score>1 means a good alignment and vice versa.

eAminomethyltransferase

fDmdA DMSP-dependent demethylase

gGlycine cleavage system T protein

hDimethylglycine dehydrogenase complexed with tetrahydrofolate 

Predicted model: C-sore=0.64; TM-score=0.80 dev= 0.09

| Rank | Class | Gene name | Organism | PDB ID | TM-scorea | RMSDb | IDENc | Covd |
| --- | --- | --- | --- | --- | --- | --- | --- | --- |
| 1 | Oxidoreductase | dmgdH | Rattus norvegicus | 4p9sA | 0.894 | 1.17 | 0.184 | 0.915 |
| 2 | Oxidoreductase | dmge | Arthrobacter globiformis | 1pj6A | 0.858 | 2.06 | 0.221 | 0.908 |
| 3 | AMT | gcvT | Pyrococcus horikoshii | 1v5vA | 0.813 | 1.70 | 0.249 | 0.843 |
| 4 | AMT | gcvT | Thermotoga maritima | 1worA | 0.809 | 0.89 | 0.284 | 0.820 |
| 5 | Oxidoreductase | soxAf | Stenotrophomonas maltophilia | 2gagA | 0.804 | 2.47 | 0.207 | 0.864 |
| 6 | Oxidoreductase | soxA | Corynebacterium sp. U-96 | 1vrqA | 0.801 | 2.49 | 0.202 | 0.862 |
| 7 | AMT | AMT | Homo sapiens | 1wsvB | 0.799 | 1.52 | 0.228 | 0.827 |
| 8 | AMT | dmdA | Pelagibacter ubique (HTCC1062) | 3tfiA | 0.792 | 1.97 | 0.258 | 0.839 |
| 9 | AMT | gcvT | Bartonella henselae | 3girA | 0.787 | 1.61 | 0.237 | 0.818 |
| 10 | AMT | gcvT | Escherichia coli K12 | 3a8iA | 0.778 | 1.90 | 0.251 | 0.816 |

abIt is a standard for measuring structural similarity between two structures

cIt is the percentage sequence identity in the structurally aligned region 

dIt represents the coverage of the alignment by TM-align and is equal to the number of structurally aligned residues divided by length of the query protein

eDimethylglycine oxidase

fHeterotetrameric sarcosine oxidase alpha-subunit

Table 21. Up: Top ten analogs identified by LOMETS for threading alignments. Case of AGI68776.1. In the middle: Model predicted by I-TASSER. Down: Top ten identified structural analogs in PDB by TM-align

| Rank | Class | Gene name | Organism | PDB ID | Iden1 (%)a | Iden2 (%)b | Covc | Norm. Z-scored |
| --- | --- | --- | --- | --- | --- | --- | --- | --- |
| 1 | AMTe | dmdAf | Pelagibacter ubique (HTCC1062) | 3tfhA | 43 | 43 | 1.00 | 4.80 |
| 2 | AMT | dmdA | Pelagibacter ubique (HTCC1062) | 3tfhA | 43 | 43 | 1.00 | 5.33 |
| 3 | AMT | dmdA | Pelagibacter ubique (HTCC1062) | 3tfhA | 43 | 43 | 1.00 | 5.72 |
| 4 | AMT | dmdA | Pelagibacter ubique (HTCC1062) | 3tfh | 44 | 43 | 0.99 | 3.27 |
| 5 | Oxidoreductase | dmgdHh | Rattus norvegicus | 4p9sA | 17 | 21 | 0.98 | 2.42 |
| 6 | AMT | dmdA | Pelagibacter ubique (HTCC1062) | 3tfhA | 43 | 43 | 1.00 | 5.80 |
| 7 | Oxidoreductase | dmgdH | Rattus norvegicus | 4p9sA | 17 | 21 | 0.98 | 3.59 |
| 8 | AMT | gcvTg | Bacillus subtilis | 1yx2A | 25 | 28 | 0.96 | 6.64 |
| 9 | AMT | dmdA | Pelagibacter ubique (HTCC1062) | 3tfhA | 43 | 43 | 1.00 | 6.79 |
| 10 | AMT | dmdA | Pelagibacter ubique (HTCC1062) | 3tfhA | 43 | 43 | 1.00 | 6.84 |

aIden1 is the percentage sequence identity of the templates in the threading aligned region with the query sequence

bIden2 is the percentage sequence identity of the whole template chains with query sequence 

cCov-Represents the coverage of the threading alignment and is equal to the number of aligned residues divided by the length of the query protein

dNorm. Z-score is the normalized Z-score of the threading alignments. Alignment with a normalized Z-score>1 means a good alignment and vice versa.

eAminomethyltransferase

fDmdA DMSP-dependent demethylase

gGlycine cleavage system T protein

hDimethylglycine dehydrogenase complexed with tetrahydrofolate 

Predicted model: C-sore=2; TM-score=0.99 dev= 0.04

| Rank | Class | Gene name | Organism | PDB ID | TM-scorea | RMSDb | IDENc | Covd |
| --- | --- | --- | --- | --- | --- | --- | --- | --- |
| 1 | AMT | dmdA | Pelagibacter ubique (HTCC1062) | 3tfhA | 0.997 | 0.40 | 0.429 | 1.000 |
| 2 | Oxidoreductase | dmgdH | Rattus norvegicus | 4p9sA | 0.932 | 1.80 | 0.180 | 0.986 |
| 3 | Oxidoreductase | dmge | Arthrobacter globiformes | 1pj6A2 | 0.915 | 2.18 | 0.244 | 0.986 |
| 4 | Oxidoreductase | dmg | Arthrobacter globiformes | 1pj7A | 0.914 | 2.18 | 0.244 | 0.986 |
| 5 | AMT | gcvT | Pyrococcus horikoshii | 1v5vA | 0.906 | 1.97 | 0.237 | 0.970 |
| 6 | Oxidoreductase | soxAf | Stenotrophomonas maltophilia | 2gagA | 0.900 | 2.29 | 0.194 | 0.975 |
| 7 | Oxidoreductase | soxA | Corynebacterium sp. U-96 | 3ad7A | 0.900 | 2.26 | 0.197 | 0.975 |
| 8 | AMT | gcvT | Thermotoga maritima | 1wooA | 0.899 | 1.74 | 0.250 | 0.951 |
| 9 | AMT | AMT | Homo sapiens | 1wsrA | 0.891 | 2.08 | 0.259 | 0.959 |
| 10 | AMT | gcvT | Bartonella henselae | 3girA | 0.885 | 1.97 | 0.210 | 0.948 |

abIt is a standard for measuring structural similarity between two structures

cIt is the percentage sequence identity in the structurally aligned region 

dIt represents the coverage of the alignment by TM-align and is equal to the number of structurally aligned residues divided by length of the query protein

eDimethylglycine oxidase

fHeterotetrameric sarcosine oxidase alpha-subunit

Table 22. Up: Top ten analogs identified by LOMETS for threading alignments. Case of AGI72139.1. In the middle: Model predicted by I-TASSER. Down: Top ten identified structural analogs in PDB by TM-align

| Rank | Class | Gene name | Organism | PDB ID | Iden1 (%)a | Iden2 (%)b | Covc | Norm. Z-scored |
| --- | --- | --- | --- | --- | --- | --- | --- | --- |
| 1 | AMTe | dmdAf | Pelagibacter ubique (HTCC1062) | 3tfhA | 43 | 43 | 1.00 | 4.79 |
| 2 | AMT | dmdA | Pelagibacter ubique (HTCC1062) | 3tfhA | 43 | 43 | 1.00 | 5.36 |
| 3 | AMT | dmdA | Pelagibacter ubique (HTCC1062) | 3tfhA | 43 | 43 | 1.00 | 5.77 |
| 4 | AMT | dmdA | Pelagibacter ubique (HTCC1062) | 3tfh | 44 | 43 | 0.99 | 3.26 |
| 5 | Oxidoreductase | dmgdHh | Rattus norvegicus | 4p9sA | 17 | 21 | 0.98 | 2.42 |
| 6 | AMT | dmdA | Pelagibacter ubique (HTCC1062) | 3thA | 43 | 43 | 1.00 | 5.77 |
| 7 | Oxidoreductase | dmgdH | Rattus norvegicus | 4p9sA | 17 | 21 | 0.97 | 3.57 |
| 8 | AMT | gcvTg | Bacillus subtilis | 1yx2A | 26 | 28 | 0.96 | 6.72 |
| 9 | AMT | dmdA | Pelagibacter ubique (HTCC1062) | 3tfhA | 43 | 43 | 1.00 | 6.75 |
| 10 | AMT | dmdA | Pelagibacter ubique (HTCC1062) | 3tfhA | 44 | 43 | 1.00 | 6.85 |

aIden1 is the percentage sequence identity of the templates in the threading aligned region with the query sequence

bIden2 is the percentage sequence identity of the whole template chains with query sequence 

cCov-Represents the coverage of the threading alignment and is equal to the number of aligned residues divided by the length of the query protein

dNorm. Z-score is the normalized Z-score of the threading alignments. Alignment with a normalized Z-score>1 means a good alignment and vice versa.

eAminomethyltransferase

fDmdA DMSP-dependent demethylase

gGlycine cleavage system T protein

hDimethylglycine dehydrogenase complexed with tetrahydrofolate 

Predicted model: C-sore=2; TM-score=0.99 dev= 0.04

| Rank | Class | Gene name | Organism | PDB ID | TM-scorea | RMSDb | IDENc | Covd |
| --- | --- | --- | --- | --- | --- | --- | --- | --- |
| 1 | AMT | dmdA | Pelagibacter ubique (HTCC1062) | 3tfhA | 0.997 | 0.39 | 0.432 | 1.000 |
| 2 | Oxidoreductase | dmgdH | Rattus norvegicus | 4p9sA | 0.932 | 1.79 | 0.183 | 0.986 |
| 3 | Oxidoreductase | dmge | Arthrobacter globiformes | 1pj6A2 | 0.915 | 2.18 | 0.252 | 0.986 |
| 4 | Oxidoreductase | dmg | Arthrobacter globiformes | 1pj7A | 0.914 | 2.18 | 0.252 | 0.986 |
| 5 | AMT | gcvT | Pyrococcus horikoshii | 1v5vA | 0.906 | 1.97 | 0.248 | 0.970 |
| 6 | Oxidoreductase | soxAf | Stenotrophomonas maltophilia | 2gagA | 0.899 | 2.30 | 0.199 | 0.975 |
| 7 | AMT | gcvT | Thermotoga maritima | 1wooA | 0.899 | 1.75 | 0.253 | 0.951 |
| 8 | Oxidoreductase | soxA | Corynebacterium sp. U-96 | 3ad7A | 0.899 | 2.28 | 0.202 | 0.975 |
| 9 | AMT | AMT | Homo sapiens | 1wsrA | 0.891 | 2.08 | 0.265 | 0.959 |
| 10 | AMT | gcvT | Bartonella henselae | 3girA | 0.885 | 1.99 | 0.210 | 0.948 |

abIt is a standard for measuring structural similarity between two structures

cIt is the percentage sequence identity in the structurally aligned region 

dIt represents the coverage of the alignment by TM-align and is equal to the number of structurally aligned residues divided by length of the query protein

eDimethylglycine oxidase

fHeterotetrameric sarcosine oxidase alpha-subunit

Table 23. Up: Top ten analogs identified by LOMETS for threading alignments. Case of AGM10509.1 In the middle: Model predicted by I-TASSER. Down: Top ten identified structural analogs in PDB by TM-align

| Rank | Class | Gene name | Organism | PDB ID | Iden1 (%)a | Iden2 (%)b | Covc | Norm. Z-scored |
| --- | --- | --- | --- | --- | --- | --- | --- | --- |
| 1 | AMTe | gcvT | Thermotoga maritima | 1wopA | 30 | 27 | 0.79 | 3.83 |
| 2 | AMT | gcvT | Pyrococcus horikoshii | 1v5vA | 26 | 26 | 0.81 | 4.85 |
| 3 | AMT | gcvT | Thermotoga maritima | 1worA | 29 | 27 | 0.79 | 4.59 |
| 4 | Oxidoreductase | dmgdH | Rattus norvegicus | 4p9s | 18 | 21 | 0.92 | 3.43 |
| 5 | Oxidoreductase | dmgdHh | Rattus norvegicus | 4p9sA | 19 | 20 | 0.83 | 2.41 |
| 6 | AMT | gcvT | Thermotoga maritima | 1wopA | 30 | 27 | 0.79 | 4.95 |
| 7 | Oxidoreductase | dmgdH | Rattus norvegicus | 4p9s | 17 | 21 | 0.92 | 3.90 |
| 8 | AMT | gcvTg | Bacillus subtilis | 1yx2A | 25 | 21 | 0.79 | 6.29 |
| 9 | AMT | dmdAf | Pelagibacter ubique (HTCC1062) | 3tfhA | 28 | 24 | 0.82 | 6.09 |
| 10 | AMT | dmdA | Pelagibacter ubique (HTCC1062) | 3tfhA | 27 | 24 | 0.82 | 5.93 |

aIden1 is the percentage sequence identity of the templates in the threading aligned region with the query sequence

bIden2 is the percentage sequence identity of the whole template chains with query sequence 

cCov-Represents the coverage of the threading alignment and is equal to the number of aligned residues divided by the length of the query protein

dNorm. Z-score is the normalized Z-score of the threading alignments. Alignment with a normalized Z-score>1 means a good alignment and vice versa.

eAminomethyltransferase

fDmdA DMSP-dependent demethylase

gGlycine cleavage system T protein

hDimethylglycine dehydrogenase complexed with tetrahydrofolate 

Predicted model: C-sore=0.55; TM-score=0.79 dev= 0.09

| Rank | Class | Gene name | Organism | PDB ID | TM-scorea | RMSDb | IDENc | Covd |
| --- | --- | --- | --- | --- | --- | --- | --- | --- |
| 1 | Oxidoreductase | dmgdH | Rattus norvegicus | 4p9sA | 0.887 | 0.40 | 0.186 | 0.913 |
| 2 | Oxidoreductase | dmge | Arthrobacter globiformis | 1pj6A | 0.855 | 2.07 | 0.214 | 0.906 |
| 3 | AMT | gcvT | Pyrococcus horikoshii | 1v5vA | 0.788 | 1.55 | 0.260 | 0.815 |
| 4 | AMT | gcvT | Thermotoga maritima | 1worA | 0.784 | 0.91 | 0.301 | 0.795 |
| 5 | Oxidoreductase | soxAf | Stenotrophomonas maltophilia | 2gagA | 0.783 | 2.52 | 0.204 | 0.842 |
| 6 | Oxidoreductase | soxA | Corynebacterium sp. U-96 | 1vrqA | 0.780 | 2.46 | 0.200 | 0.837 |
| 7 | AMT | AMT(GCST) | Homo sapiens | 1wsvB | 0.769 | 1.56 | 0.221 | 0.797 |
| 8 | AMT | dmdA | Pelagibacter ubique HTCC1062 | 3tfiA | 0.768 | 2.02 | 0.257 | 0.817 |
| 9 | AMT | gcvT | Bartonella henselae | 3girA | 0.757 | 1.66 | 0.241 | 0.788 |
| 10 | AMT | gcvT | Bacillus subtilis 168 | 1yx2B | 0.753 | 1.58 | 0.246 | 0.781 |

abIt is a standard for measuring structural similarity between two structures

cIt is the percentage sequence identity in the structurally aligned region 

dIt represents the coverage of the alignment by TM-align and is equal to the number of structurally aligned residues divided by length of the query protein

eDimethylglycine oxidase

fHeterotetrameric sarcosine oxidase alpha-subunit

Table 24. Up: Top ten analogs identified by LOMETS for threading alignments. Case of AHD01041.1. In the middle: Model predicted by I-TASSER. Down: Top ten identified structural analogs in PDB by TM-align

| Rank | Class | Gene name | Organism | PDB ID | Iden1 (%)a | Iden2 (%)b | Covc | Norm. Z-scored |
| --- | --- | --- | --- | --- | --- | --- | --- | --- |
| 1 | AMTe | dmdAf | Pelagibacter ubique (HTCC1062) | 3tfhA | 42 | 42 | 1.00 | 4.76 |
| 2 | AMT | dmdA | Pelagibacter ubique (HTCC1062) | 3tfhA | 41 | 42 | 1.00 | 5.33 |
| 3 | AMT | dmdA | Pelagibacter ubique (HTCC1062) | 3tfhA | 42 | 42 | 1.00 | 5.73 |
| 4 | Oxidoreductase | dmgdH | Rattus norvegicus | 4p9sA | 20 | 22 | 0.98 | 3.27 |
| 5 | Oxidoreductase | dmgdHh | Rattus norvegicus | 4p9sA | 18 | 22 | 0.98 | 2.42 |
| 6 | AMT | gcvT | Pelagibacter ubique (HTCC1062) | 3tfhA | 41 | 42 | 1.00 | 5.67 |
| 7 | Oxidoreductase | dmgdH | Rattus norvegicus | 4p9sA | 18 | 22 | 0.98 | 3.60 |
| 8 | AMT | gcvTg | Bacillus subtilis | 1yx2A | 24 | 25 | 0.96 | 6.32 |
| 9 | AMT | dmdA | Pelagibacter ubique (HTCC1062) | 3tfhA | 42 | 42 | 1.00 | 7.74 |
| 10 | AMT | dmdA | Pelagibacter ubique (HTCC1062) | 3tfhA | 41 | 42 | 1.00 | 6.82 |

aIden1 is the percentage sequence identity of the templates in the threading aligned region with the query sequence

bIden2 is the percentage sequence identity of the whole template chains with query sequence 

cCov-Represents the coverage of the threading alignment and is equal to the number of aligned residues divided by the length of the query protein

dNorm. Z-score is the normalized Z-score of the threading alignments. Alignment with a normalized Z-score>1 means a good alignment and vice versa.

eAminomethyltransferase

fDmdA DMSP-dependent demethylase

gGlycine cleavage system T protein

hDimethylglycine dehydrogenase complexed with tetrahydrofolate 

Predicted model: C-sore=1.69; TM-score=0.95 dev= 0.05

| Rank | Class | Gene name | Organism | PDB ID | TM-scorea | RMSDb | IDENc | Covd |
| --- | --- | --- | --- | --- | --- | --- | --- | --- |
| 1 | Oxidoreductase | dmdA | Pelagibacter ubique HTCC1062 | 3tfhA | 0.990 | 0.71 | 0.412 | 1.000 |
| 2 | Oxidoreductase | dmge | Arthrobacter globiformis | 1pj6A | 0.943 | 1.59 | 0.189 | 0.986 |
| 3 | AMT | gcvT | Pyrococcus horikoshii | 1v5vA | 0.927 | 2.01 | 0.253 | 0.989 |
| 4 | AMT | gcvT | Thermotoga maritima | 1worA | 0.926 | 2.02 | 0.253 | 0.989 |
| 5 | Oxidoreductase | soxAf | Stenotrophomonas maltophilia | 2gagA | 0.916 | 1.89 | 0.228 | 0.975 |
| 6 | Oxidoreductase | soxA | Corynebacterium sp. U-96 | 1vrqA | 0.910 | 1.69 | 0.235 | 0.959 |
| 7 | AMT | AMT(GCST) | Homo sapiens | 1wsvB | 0.909 | 2.22 | 0.199 | 0.981 |
| 8 | AMT | dmdA | Pelagibacter ubique HTCC1062 | 3tfiA | 0.908 | 2.19 | 0.191 | 0.981 |
| 9 | AMT | gcvT | Bartonella henselae | 3girA | 0.901 | 1.87 | 0.244 | 0.959 |
| 10 | AMT | gcvT | Bacillus subtilis 168 | 1yx2B | 0.891 | 1.82 | 0.230 | 0.945 |

abIt is a standard for measuring structural similarity between two structures

cIt is the percentage sequence identity in the structurally aligned region 

dIt represents the coverage of the alignment by TM-align and is equal to the number of structurally aligned residues divided by length of the query protein

eDimethylglycine oxidase

fHeterotetrameric sarcosine oxidase alpha-subunit

Table 25. Up: Top ten analogs identified by LOMETS for threading alignments. Case of AHI32422.1. In the middle: Model predicted by I-TASSER. Down: Top ten identified structural analogs in PDB by TM-align

| Rank | Class | Gene name | Organism | PDB ID | Iden1 (%)a | Iden2 (%)b | Covc | Norm. Z-scored |
| --- | --- | --- | --- | --- | --- | --- | --- | --- |
| 1 | AMTe | gcvT | Thermotoga maritima | 1wopA | 29 | 25 | 0.79 | 3.70 |
| 2 | AMT | gcvT | Pyrococcus horikoshii | 1v5vA | 24 | 24 | 0.81 | 4.82 |
| 3 | AMT | gcvT | Thermotoga maritima | 1worA | 29 | 25 | 0.79 | 4.59 |
| 4 | Oxidoreductase | dmgdH | Rattus norvegicus | 4p9s | 17 | 19 | 0.92 | 3.51 |
| 5 | Oxidoreductase | dmgdHh | Rattus norvegicus | 4p9sA | 19 | 19 | 0.82 | 2.42 |
| 6 | AMT | gcvT | Thermotoga maritima | 1wopA | 29 | 25 | 0.79 | 4.90 |
| 7 | Oxidoreductase | dmgdH | Rattus norvegicus | 4p9s | 18 | 19 | 0.83 | 3.64 |
| 8 | AMT | gcvTg | Bacillus subtilis | 1yx2A | 25 | 20 | 0.78 | 6.21 |
| 9 | AMT | dmdAf | Pelagibacter ubique (HTCC1062) | 3tfhA | 28 | 24 | 0.81 | 6.13 |
| 10 | AMT | dmdA | Pelagibacter ubique (HTCC1062) | 3tfhA | 28 | 24 | 0.81 | 5.96 |

aIden1 is the percentage sequence identity of the templates in the threading aligned region with the query sequence

bIden2 is the percentage sequence identity of the whole template chains with query sequence 

cCov-Represents the coverage of the threading alignment and is equal to the number of aligned residues divided by the length of the query protein

dNorm. Z-score is the normalized Z-score of the threading alignments. Alignment with a normalized Z-score>1 means a good alignment and vice versa.

eAminomethyltransferase

fDmdA DMSP-dependent demethylase

gGlycine cleavage system T protein

hDimethylglycine dehydrogenase complexed with tetrahydrofolate 

Predicted model: C-sore=0.61; TM-score=0.80 dev= 0.09

| Rank | Class | Gene name | Organism | PDB ID | TM-scorea | RMSDb | IDENc | Covd |
| --- | --- | --- | --- | --- | --- | --- | --- | --- |
| 1 | Oxidoreductase | dmgdH | Rattus norvegicus | 4p9sA | 0.896 | 1.16 | 0.176 | 0.914 |
| 2 | Oxidoreductase | dmge | Arthrobacter globiformis | 1pj6A | 0.860 | 1.97 | 0.207 | 0.907 |
| 3 | AMT | gcvT | Pyrococcus horikoshii | 1v5vA | 0.776 | 1.78 | 0.238 | 0.808 |
| 4 | AMT | gcvT | Thermotoga maritima | 1worA | 0.774 | 0.96 | 0.289 | 0.786 |
| 5 | Oxidoreductase | soxAf | Stenotrophomonas maltophilia | 2gagA | 0.774 | 2.71 | 0.211 | 0.837 |
| 6 | Oxidoreductase | soxA | Corynebacterium sp. U-96 | 3ad7A | 0.771 | 2.46 | 0.202 | 0.828 |
| 7 | AMT | dmdA | Pelagibacter ubique HTCC1062 | 3tfiA | 0.758 | 2.09 | 0.262 | 0.808 |
| 8 | AMT | AMT (GCST) | Homo sapiens | 1wsvB | 0.758 | 1.55 | 0.216 | 0.786 |
| 9 | AMT | gcvT | Bartonella henselae | 3girA | 0.747 | 1.67 | 0.247 | 0.777 |
| 10 | AMT | gcvT | Bacillus subtilis 168 | 1yx2B | 0.743 | 1.65 | 0.251 | 0.773 |

abIt is a standard for measuring structural similarity between two structures

cIt is the percentage sequence identity in the structurally aligned region 

dIt represents the coverage of the alignment by TM-align and is equal to the number of structurally aligned residues divided by length of the query protein

eDimethylglycine oxidase

fHeterotetrameric sarcosine oxidase alpha-subunit

Table 26. Up: Top ten analogs identified by LOMETS for threading alignments. Case of AHM03102.1. In the middle: Model predicted by I-TASSER. Down: Top ten identified structural analogs in PDB by TM-align

| Rank | Class | Gene name | Organism | PDB ID | Iden1 (%)a | Iden2 (%)b | Covc | Norm. Z-scored |
| --- | --- | --- | --- | --- | --- | --- | --- | --- |
| 1 | AMTe | dmdAf | Pelagibacter ubique (HTCC1062) | 3tfhA | 37 | 36 | 0.99 | 4.64 |
| 2 | AMT | dmdA | Pelagibacter ubique (HTCC1062) | 3tfhA | 37 | 36 | 0.99 | 5.36 |
| 3 | AMT | dmdA | Pelagibacter ubique (HTCC1062) | 3tfhA | 36 | 36 | 0.99 | 5.48 |
| 4 | Oxidoreductase | dmgdH | Rattus norvegicus | 4p9s | 21 | 24 | 0.97 | 3.25 |
| 5 | Oxidoreductase | dmgdHh | Rattus norvegicus | 4p9sA | 20 | 24 | 0.98 | 2.42 |
| 6 | AMT | dmdA | Pelagibacter ubique (HTCC1062) | 3tfhA | 36 | 36 | 0.98 | 5.68 |
| 7 | Oxidoreductase | dmgdH | Rattus norvegicus | 4p9s | 19 | 24 | 0.97 | 3.59 |
| 8 | AMT | gcvTg | Bacillus subtilis | 1yx2A | 24 | 26 | 0.95 | 6.66 |
| 9 | AMT | dmdA | Pelagibacter ubique (HTCC1062) | 3tfhA | 37 | 36 | 0.99 | 7.41 |
| 10 | AMT | dmdA | Pelagibacter ubique (HTCC1062) | 3tfhA | 36 | 36 | 0.99 | 6.61 |

aIden1 is the percentage sequence identity of the templates in the threading aligned region with the query sequence

bIden2 is the percentage sequence identity of the whole template chains with query sequence 

cCov-Represents the coverage of the threading alignment and is equal to the number of aligned residues divided by the length of the query protein

dNorm. Z-score is the normalized Z-score of the threading alignments. Alignment with a normalized Z-score>1 means a good alignment and vice versa.

eAminomethyltransferase

fDmdA DMSP-dependent demethylase

gGlycine cleavage system T protein

hDimethylglycine dehydrogenase complexed with tetrahydrofolate 

Predicted model: C-sore=1.69; TM-score=0.95 dev= 0.05

| Rank | Class | Gene name | Organism | PDB ID | TM-scorea | RMSDb | IDENc | Covd |
| --- | --- | --- | --- | --- | --- | --- | --- | --- |
| 1 | AMT | dmdA | Pelagibacter ubique HTCC1062 | 3tfiA | 0.981 | 0.63 | 0.367 | 0.989 |
| 2 | Oxidoreductase | dmgdH | Rattus norvegicus | 4p9sA | 0.931 | 1.65 | 0.209 | 0.976 |
| 3 | Oxidoreductase | dmge | Arthrobacter globiformis | 1pj6A | 0.912 | 2.10 | 0.259 | 0.976 |
| 4 | AMT | gcvT | Pyrococcus horikoshii | 1v5vA | 0.906 | 1.94 | 0.226 | 0.965 |
| 5 | Oxidoreductase | soxAf | Stenotrophomonas maltophilia | 2gagA | 0.894 | 2.24 | 0.217 | 0.965 |
| 6 | Oxidoreductase | soxA | Corynebacterium sp. U-96 | 3ad7A | 0.894 | 2.21 | 0.228 | 0.965 |
| 7 | AMT | gcvT | Thermotoga maritima | 1worA | 0.894 | 1.69 | 0.251 | 0.941 |
| 8 | AMT | AMT (GCST) | Homo sapiens | 1wsvA | 0.888 | 1.98 | 0.232 | 0.949 |
| 9 | AMT | gcvT | Bartonella henselae | 3girA | 0.880 | 1.87 | 0.210 | 0.935 |
| 10 | AMT | gcvT | Bacillus subtilis 168 | 1yx2B | 0.875 | 2.09 | 0.243 | 0.941 |

abIt is a standard for measuring structural similarity between two structures

cIt is the percentage sequence identity in the structurally aligned region 

dIt represents the coverage of the alignment by TM-align and is equal to the number of structurally aligned residues divided by length of the query protein

eDimethylglycine oxidase

fHeterotetrameric sarcosine oxidase alpha-subunit

Table 27. Up: Top ten analogs identified by LOMETS for threading alignments. Case of AHM05061.1. In the middle: Model predicted by I-TASSER. Down: Top ten identified structural analogs in PDB by TM-align

| Rank | Class | Gene name | Organism | PDB ID | Iden1 (%)a | Iden2 (%)b | Covc | Norm. Z-scored |
| --- | --- | --- | --- | --- | --- | --- | --- | --- |
| 1 | AMTe | dmdAf | Pelagibacter ubique (HTCC1062) | 3tfhA | 43 | 43 | 0.99 | 4.59 |
| 2 | AMT | dmdA | Pelagibacter ubique (HTCC1062) | 3tfhA | 43 | 43 | 0.99 | 5.24 |
| 3 | AMT | dmdA | Pelagibacter ubique (HTCC1062) | 3tfhA | 42 | 43 | 0.99 | 5.45 |
| 4 | AMT | dmdA | Pelagibacter ubique HTCC1062 | 3tfh | 43 | 43 | 0.99 | 3.29 |
| 5 | Oxidoreductase | dmgdHh | Rattus norvegicus | 4p9sA | 17 | 19 | 0.98 | 2.42 |
| 6 | AMT | dmdA | Pelagibacter ubique (HTCC1062) | 3tfhA | 42 | 43 | 0.99 | 5.59 |
| 7 | AMT | dmdA | Pelagibacter ubique HTCC1062 | 3tfh | 42 | 43 | 0.98 | 3.63 |
| 8 | AMT | gcvTg | Bacillus subtilis | 1yx2A | 27 | 28 | 0.96 | 6.47 |
| 9 | AMT | dmdA | Pelagibacter ubique (HTCC1062) | 3tfhA | 43 | 43 | 1.00 | 7.59 |
| 10 | AMT | dmdA | Pelagibacter ubique (HTCC1062) | 3tfhA | 42 | 43 | 1.00 | 6.55 |

aIden1 is the percentage sequence identity of the templates in the threading aligned region with the query sequence

bIden2 is the percentage sequence identity of the whole template chains with query sequence 

cCov-Represents the coverage of the threading alignment and is equal to the number of aligned residues divided by the length of the query protein

dNorm. Z-score is the normalized Z-score of the threading alignments. Alignment with a normalized Z-score>1 means a good alignment and vice versa.

eAminomethyltransferase

fDmdA DMSP-dependent demethylase

gGlycine cleavage system T protein

hDimethylglycine dehydrogenase complexed with tetrahydrofolate 

Predicted model: C-sore=2.00; TM-score=0.99 dev= 0.04

| Rank | Class | Gene name | Organism | PDB ID | TM-scorea | RMSDb | IDENc | Covd |
| --- | --- | --- | --- | --- | --- | --- | --- | --- |
| 1 | AMT | dmdA | Pelagibacter ubique HTCC1062 | 3tfhA | 0.981 | 0.63 | 0.367 | 0.989 |
| 2 | Oxidoreductase | dmgdH | Rattus norvegicus | 4p9sA | 0.931 | 1.65 | 0.209 | 0.976 |
| 3 | Oxidoreductase | dmge | Arthrobacter globiformis | 1pj6A2 | 0.912 | 2.10 | 0.259 | 0.976 |
| 4 | Oxidoreductase | dmg | Arthrobacter globiformis | 1pj7A | 0.906 | 1.94 | 0.226 | 0.965 |
| 5 | AMT | gcvT | Pyrococcus horikoshii | 1v5vA | 0.894 | 2.24 | 0.217 | 0.965 |
| 6 | Oxidoreductase | soxA | Corynebacterium sp. U-96 | 3ad7A | 0.894 | 2.21 | 0.228 | 0.965 |
| 7 | Oxidoreductase | soxAf | Stenotrophomonas maltophilia | 2gagA | 0.894 | 1.69 | 0.251 | 0.941 |
| 8 | AMT | gcvT | Thermotoga maritima | 1wooA | 0.888 | 1.98 | 0.232 | 0.949 |
| 9 | AMT | AMT(GCST) | Homo sapiens | 1wsrA | 0.880 | 1.87 | 0.210 | 0.935 |
| 10 | AMT | gcvT | Bartonella henselae | 3girA | 0.875 | 2.09 | 0.243 | 0.941 |

abIt is a standard for measuring structural similarity between two structures

cIt is the percentage sequence identity in the structurally aligned region 

dIt represents the coverage of the alignment by TM-align and is equal to the number of structurally aligned residues divided by length of the query protein

eDimethylglycine oxidase

fHeterotetrameric sarcosine oxidase alpha-subunit

Table 28. Up: Top ten analogs identified by LOMETS for threading alignments. Case of ASJ73090.1. In the middle: Model predicted by I-TASSER. Down: Top ten identified structural analogs in PDB by TM-align

| Rank | Class | Gene name | Organism | PDB ID | Iden1 (%)a | Iden2 (%)b | Covc | Norm. Z-scored |
| --- | --- | --- | --- | --- | --- | --- | --- | --- |
| 1 | AMTe | dmdAf | Pelagibacter ubique (HTCC1062) | 3tfhA | 41 | 40 | 0.96 | 4.69 |
| 2 | AMT | dmdA | Pelagibacter ubique (HTCC1062) | 3tfhA | 41 | 40 | 0.96 | 5.45 |
| 3 | AMT | dmdA | Pelagibacter ubique (HTCC1062) | 3tfhA | 41 | 40 | 0.96 | 5.56 |
| 4 | AMT | dmdA | Pelagibacter ubique HTCC1062 | 3tfh | 41 | 40 | 0.95 | 3.24 |
| 5 | Oxidoreductase | dmgdHh | Rattus norvegicus | 4p9sA | 20 | 24 | 0.96 | 2.40 |
| 6 | AMT | dmdA | Pelagibacter ubique (HTCC1062) | 3tfhA | 41 | 40 | 0.96 | 5.72 |
| 7 | Oxidoreductase | dmgdH | Rattus norvegicus | 4p9s | 19 | 24 | 0.95 | 3.58 |
| 8 | AMT | gcvTg | Bacillus subtilis | 1yx2A | 21 | 23 | 0.92 | 6.47 |
| 9 | AMT | dmdA | Pelagibacter ubique (HTCC1062) | 3tfhA | 41 | 40 | 0.96 | 7.97 |
| 10 | AMT | dmdA | Pelagibacter ubique (HTCC1062) | 3tfhA | 41 | 40 | 0.96 | 6.69 |

aIden1 is the percentage sequence identity of the templates in the threading aligned region with the query sequence

bIden2 is the percentage sequence identity of the whole template chains with query sequence 

cCov-Represents the coverage of the threading alignment and is equal to the number of aligned residues divided by the length of the query protein

dNorm. Z-score is the normalized Z-score of the threading alignments. Alignment with a normalized Z-score>1 means a good alignment and vice versa.

eAminomethyltransferase

fDmdA DMSP-dependent demethylase

gGlycine cleavage system T protein

hDimethylglycine dehydrogenase complexed with tetrahydrofolate 

Predicted model: C-sore=1.77; TM-score=0.96 dev= 0.05

| Rank | Class | Gene name | Organism | PDB ID | TM-scorea | RMSDb | IDENc | Covd |
| --- | --- | --- | --- | --- | --- | --- | --- | --- |
| 1 | AMT | dmdA | Pelagibacter ubique HTCC1062 | 3tfhA | 0.951 | 0.51 | 0.409 | 0.956 |
| 2 | Oxidoreductase | dmgdH | Rattus norvegicus | 4p9sA | 0.914 | 1.90 | 0.199 | 0.969 |
| 3 | Oxidoreductase | dmge | Arthrobacter globiformis | 1pj6A2 | 0.886 | 2.51 | 0.226 | 0.966 |
| 4 | AMT | gcvT | Pyrococcus horikoshii | 1v5vA | 0.877 | 2.24 | 0.245 | 0.948 |
| 5 | Oxidoreductase | soxAf | Stenotrophomonas maltophilia | 2gagA | 0.874 | 2.52 | 0.175 | 0.953 |
| 6 | Oxidoreductase | soxA | Corynebacterium sp. U-96 | 3ad7A | 0.873 | 2.49 | 0.183 | 0.953 |
| 7 | AMT | gcvT | Thermotoga maritima | 1wooA | 0.867 | 1.87 | 0.244 | 0.919 |
| 8 | AMT | AMT(GCST) | Homo sapiens | 1wsrA | 0.860 | 2.27 | 0.221 | 0.932 |
| 9 | AMT | gcvT | Bartonella henselae | 3girA | 0.856 | 2.02 | 0.205 | 0.917 |
| 10 | AMT | gcvT | Bacillus subtilis | 1yx2B | 0.846 | 2.11 | 0.211 | 0.911 |

abIt is a standard for measuring structural similarity between two structures

cIt is the percentage sequence identity in the structurally aligned region 

dIt represents the coverage of the alignment by TM-align and is equal to the number of structurally aligned residues divided by length of the query protein

eDimethylglycine oxidase

fHeterotetrameric sarcosine oxidase alpha-subunit

Table 29. Up: Top ten analogs identified by LOMETS for threading alignments. Case of CBV41552.1. In the middle: Model predicted by I-TASSER. Down: Top ten identified structural analogs in PDB by TM-align

| Rank | Class | Gene name | Organism | PDB ID | Iden1 (%)a | Iden2 (%)b | Covc | Norm. Z-scored |
| --- | --- | --- | --- | --- | --- | --- | --- | --- |
| 1 | AMTe | gcvT | Thermotoga maritima | 1wopA | 32 | 26 | 0.75 | 3.52 |
| 2 | AMT | dmdAf | Pelagibacter ubique (HTCC1062) | 3tfhA | 29 | 24 | 0.77 | 4.82 |
| 3 | AMT | gcvT | Thermotoga  maritima | 1worA | 31 | 26 | 0.75 | 4.65 |
| 4 | AMT | dmgdH | Rattus norvegicus | 4p9s | 19 | 22 | 0.92 | 3.50 |
| 5 | Oxidoreductase | dmgdHh | Rattus norvegicus | 4p9sA | 20 | 20 | 0.80 | 2.42 |
| 6 | AMT | gcvT | Thermotoga maritima | 1wopA | 32 | 26 | 0.75 | 4.81 |
| 7 | Oxidoreductase | dmgdH | Rattus norvegicus | 4p9s | 18 | 22 | 0.88 | 3.91 |
| 8 | AMT | gcvTg | Bacillus subtilis | 1yx2A | 27 | 21 | 0.74 | 6.40 |
| 9 | AMT | dmdA | Pelagibacter ubique (HTCC1062) | 3tfhA | 29 | 24 | 0.77 | 5.62 |
| 10 | AMT | dmdA | Pelagibacter ubique (HTCC1062) | 3tfhA | 29 | 24 | 0.77 | 5.96 |

aIden1 is the percentage sequence identity of the templates in the threading aligned region with the query sequence

bIden2 is the percentage sequence identity of the whole template chains with query sequence 

cCov-Represents the coverage of the threading alignment and is equal to the number of aligned residues divided by the length of the query protein

dNorm. Z-score is the normalized Z-score of the threading alignments. Alignment with a normalized Z-score>1 means a good alignment and vice versa.

eAminomethyltransferase

fDmdA DMSP-dependent demethylase

gGlycine cleavage system T protein

hDimethylglycine dehydrogenase complexed with tetrahydrofolate 

Predicted model: C-sore=0.68; TM-score=0.81 dev= 0.09

| Rank | Class | Gene name | Organism | PDB ID | TM-scorea | RMSDb | IDENc | Covd |
| --- | --- | --- | --- | --- | --- | --- | --- | --- |
| 1 | AMT | dmgdH | Rattus norvegicus | 4p9sA | 0.906 | 1.31 | 0.184 | 0.928 |
| 2 | Oxidoreductase | dmge | Arthrobacter globiformis | 1pj6A2 | 0.878 | 2.00 | 0.200 | 0.924 |
| 3 | Oxidoreductase | soxAf | Stenotrophomonas maltophilia | 2gagA | 0.762 | 2.78 | 0.209 | 0.825 |
| 4 | Oxidoreductase | soxA | Corynebacterium sp. | 3ad8A | 0.760 | 2.88 | 0.202 | 0.825 |
| 5 | AMT | dmdA | Pelagibacter ubique HTCC1062 | 3tfiA | 0.742 | 1.81 | 0.285 | 0.777 |
| 6 | AMT | gcvT | Thermotoga maritima | 1worA | 0.738 | 1.10 | 0.314 | 0.752 |
| 7 | AMT | gcvT | Pyrococcus horikoshii | 1v5vA | 0.737 | 1.63 | 0.266 | 0.766 |
| 8 | AMT | AMT(GCST) | Homo sapiens | 1wsrA | 0.731 | 1.66 | 0.202 | 0.760 |
| 9 | AMT | gcvT | Bartonella henselae | 3girA | 0.722 | 1.70 | 0.246 | 0.752 |
| 10 | AMT | gcvT | Bacillus subtilis | 1yx2B | 0.712 | 1.52 | 0.269 | 0.737 |

abIt is a standard for measuring structural similarity between two structures

cIt is the percentage sequence identity in the structurally aligned region 

dIt represents the coverage of the alignment by TM-align and is equal to the number of structurally aligned residues divided by length of the query protein

eDimethylglycine oxidase

fHeterotetrameric sarcosine oxidase alpha-subunit

Table 30. Up: Top ten analogs identified by LOMETS for threading alignments. Case of WP_047029467. In the middle: Model predicted by I-TASSER. Down: Top ten identified structural analogs in PDB by TM-align

| Rank | Class | Gene name | Organism | PDB ID | Iden1 (%)a | Iden2 (%)b | Covc | Norm. Z-scored |
| --- | --- | --- | --- | --- | --- | --- | --- | --- |
| 1 | AMTe | dmdAf | Pelagibacter ubique (HTCC1062) | 3tfhA | 42 | 42 | 1.00 | 4.90 |
| 2 | AMT | dmdA | Pelagibacter ubique (HTCC1062) | 3tfhA | 42 | 42 | 1.00 | 5.45 |
| 3 | AMT | dmdA | Pelagibacter ubique (HTCC1062) | 3tfhA | 42 | 42 | 1.00 | 5.87 |
| 4 | AMT | dmdA | Pelagibacter ubique (HTCC1062) | 3tfh | 42 | 42 | 0.99 | 3.30 |
| 5 | Oxidoreductase | dmgdHh | Rattus norvegicus | 4p9sA | 19 | 20 | 0.98 | 2.42 |
| 6 | AMT | dmdA | Pelagibacter ubique (HTCC1062) | 3tfhA | 42 | 42 | 0.99 | 5.76 |
| 7 | Oxidoreductase | dmdA | Pelagibacter ubique (HTCC1062) | 3tfh | 42 | 42 | 0.98 | 3.62 |
| 8 | AMT | gcvTg | Bacillus subtilis | 1yx2A | 25 | 26 | 0.96 | 6.40 |
| 9 | AMT | dmdA | Pelagibacter ubique (HTCC1062) | 3tfhA | 42 | 42 | 1.00 | 8.06 |
| 10 | AMT | dmdA | Pelagibacter ubique (HTCC1062) | 3tfhA | 42 | 42 | 1.00 | 6.93 |

aIden1 is the percentage sequence identity of the templates in the threading aligned region with the query sequence

bIden2 is the percentage sequence identity of the whole template chains with query sequence 

cCov-Represents the coverage of the threading alignment and is equal to the number of aligned residues divided by the length of the query protein

dNorm. Z-score is the normalized Z-score of the threading alignments. Alignment with a normalized Z-score>1 means a good alignment and vice versa.

eAminomethyltransferase

fDmdA DMSP-dependent demethylase

gGlycine cleavage system T protein

hDimethylglycine dehydrogenase complexed with tetrahydrofolate 

Predicted model: C-sore=2; TM-score=0.99 dev= 0.04

| Rank | Class | Gene name | Organism | PDB ID | TM-scorea | RMSDb | IDENc | Covd |
| --- | --- | --- | --- | --- | --- | --- | --- | --- |
| 1 | AMT | dmdA | Pelagibacter ubique (HTCC1062) | 3tfhA | 0.997 | 0.36 | 0.421 | 1.000 |
| 2 | Oxidoreductase | dmgdH | Rattus norvegicus | 4p9sA | 0.928 | 1.86 | 0.185 | 0.984 |
| 3 | Oxidoreductase | dmge | Arthrobacter globiformis | 1pj6A | 0.911 | 2.23 | 0.260 | 0.984 |
| 4 | AMT | gcvT | Pyrococcus horikoshii | 1v5vA | 0.902 | 1.95 | 0.225 | 0.965 |
| 5 | Oxidoreductase | soxAf | Stenotrophomonas maltophilia | 2gagA | 0.897 | 2.31 | 0.209 | 0.973 |
| 6 | Oxidoreductase | soxA | Corynebacterium sp. U-96 | 3ad7A | 0.897 | 2.37 | 0.207 | 0.976 |
| 7 | AMT | gcvT | Thermotoga maritima | 1wooA | 0.895 | 1.74 | 0.261 | 0.946 |
| 8 | AMT | AMT(GCST) | Homo sapiens | 1wsrA | 0.889 | 2.08 | 0.239 | 0.957 |
| 9 | AMT | gcvT | Bartonella henselae | 3girA | 0.883 | 1.94 | 0.222 | 0.943 |
| 10 | AMT | gcvT | Bacillus subtilis | 1yx2B | 0.877 | 2.10 | 0.253 | 0.946 |

abIt is a standard for measuring structural similarity between two structures

cIt is the percentage sequence identity in the structurally aligned region 

dIt represents the coverage of the alignment by TM-align and is equal to the number of structurally aligned residues divided by length of the query protein

eDimethylglycine oxidase

fHeterotetrameric sarcosine oxidase alpha-subunit

Table 31. Up: Top ten analogs identified by LOMETS for threading alignments. Case of WP_048536000. In the middle: Model predicted by I-TASSER. Down: Top ten identified structural analogs in PDB by TM-align

| Rank | Class | Gene name | Organism | PDB ID | Iden1 (%)a | Iden2 (%)b | Covc | Norm. Z-scored |
| --- | --- | --- | --- | --- | --- | --- | --- | --- |
| 1 | AMTe | dmdAf | Pelagibacter ubique (HTCC1062) | 3tfhA | 44 | 44 | 1.00 | 4.77 |
| 2 | AMT | dmdA | Pelagibacter ubique (HTCC1062) | 3tfhA | 44 | 44 | 1.00 | 5.36 |
| 3 | AMT | dmdA | Pelagibacter ubique (HTCC1062) | 3tfhA | 44 | 44 | 1.00 | 5.80 |
| 4 | AMT | dmdA | Pelagibacter ubique (HTCC1062) | 3tfh | 44 | 44 | 0.99 | 3.19 |
| 5 | Oxidoreductase | dmgdHh | Rattus norvegicus | 4p9sA | 17 | 20 | 0.97 | 2.35 |
| 6 | AMTe | dmdA | Pelagibacter ubique (HTCC1062) | 3tfhA | 44 | 44 | 0.99 | 5.70 |
| 7 | Oxidoreductase | dmdA | Pelagibacter ubique (HTCC1062) | 3tfh | 43 | 44 | 0.98 | 3.52 |
| 8 | AMT | gcvTg | Bacillus subtilis | 1yx2A | 25 | 27 | 0.96 | 6.47 |
| 9 | AMT | dmdA | Pelagibacter ubique (HTCC1062) | 3tfhA | 44 | 44 | 1.00 | 7.58 |
| 10 | AMT | dmdA | Pelagibacter ubique (HTCC1062) | 3tfhA | 44 | 44 | 1.00 | 6.78 |

aIden1 is the percentage sequence identity of the templates in the threading aligned region with the query sequence

bIden2 is the percentage sequence identity of the whole template chains with query sequence 

cCov-Represents the coverage of the threading alignment and is equal to the number of aligned residues divided by the length of the query protein

dNorm. Z-score is the normalized Z-score of the threading alignments. Alignment with a normalized Z-score>1 means a good alignment and vice versa.

eAminomethyltransferase

fDmdA DMSP-dependent demethylase

gGlycine cleavage system T protein

hDimethylglycine dehydrogenase complexed with tetrahydrofolate 

Predicted model: C-sore=2; TM-score=0.99 dev= 0.04

| Rank | Class | Gene name | Organism | PDB ID | TM-scorea | RMSDb | IDENc | Covd |
| --- | --- | --- | --- | --- | --- | --- | --- | --- |
| 1 | AMT | dmdA | Pelagibacter ubique (HTCC1062) | 3tfhA | 0.997 | 0.36 | 0.421 | 1.000 |
| 2 | Oxidoreductase | dmgdH | Rattus norvegicus | 4p9sA | 0.928 | 1.86 | 0.185 | 0.984 |
| 3 | Oxidoreductase | dmge | Arthrobacter globiformis | 1pj6A2 | 0.911 | 2.23 | 0.260 | 0.984 |
| 4 | Oxidoreductase | dmg | Arthrobacter globiformis | 1pj7A | 0.902 | 1.95 | 0.225 | 0.965 |
| 5 | AMT | gcvT | Pyrococcus horikoshii | 1v5vA | 0.897 | 2.31 | 0.209 | 0.973 |
| 6 | Oxidoreductase | soxAf | Stenotrophomonas maltophilia | 2gagA | 0.897 | 2.37 | 0.207 | 0.976 |
| 7 | Oxidoreductase | soxA | Corynebacterium sp. U-96 | 1vrqA | 0.895 | 1.74 | 0.261 | 0.946 |
| 8 | AMT | gcvT | Thermotoga maritima | 1wooA | 0.889 | 2.08 | 0.239 | 0.957 |
| 9 | AMT | AMT(GCST) | Homo sapiens | 1wsrA | 0.883 | 1.94 | 0.222 | 0.943 |
| 10 | AMT | gcvT | Bartonella henselae | 3girA | 0.877 | 2.10 | 0.253 | 0.946 |

abIt is a standard for measuring structural similarity between two structures

cIt is the percentage sequence identity in the structurally aligned region 

dIt represents the coverage of the alignment by TM-align and is equal to the number of structurally aligned residues divided by length of the query protein

eDimethylglycine oxidase

fHeterotetrameric sarcosine oxidase alpha-subunit

Table 32. Up: Top ten analogs identified by LOMETS for threading alignments. Case of WP_049834197. In the middle: Model predicted by I-TASSER. Down: Top ten identified structural analogs in PDB by TM-align

| Rank | Class | Gene name | Organism | PDB ID | Iden1 (%)a | Iden2 (%)b | Covc | Norm. Z-scored |
| --- | --- | --- | --- | --- | --- | --- | --- | --- |
| 1 | AMTe | dmdAf | Pelagibacter ubique (HTCC1062) | 3tfhA | 44 | 45 | 1.00 | 4.83 |
| 2 | AMT | dmdA | Pelagibacter ubique (HTCC1062) | 3tfhA | 44 | 45 | 1.00 | 5.42 |
| 3 | AMT | dmdA | Pelagibacter ubique (HTCC1062) | 3tfhA | 44 | 45 | 1.00 | 5.76 |
| 4 | AMT | dmdA | Pelagibacter ubique (HTCC1062) | 3tfh | 44 | 45 | 0.99 | 3.20 |
| 5 | Oxidoreductase | dmgdHh | Rattus norvegicus | 4p9sA | 18 | 22 | 0.98 | 2.36 |
| 6 | AMT | dmdA | Pelagibacter ubique (HTCC1062) | 3tfhA | 44 | 45 | 0.99 | 5.87 |
| 7 | Oxidoreductase | dmgdH | Rattus norvegicus | 4p9sA | 18 | 22 | 0.98 | 3.51 |
| 8 | AMT | gcvTg | Bacillus subtilis | 1yx2A | 25 | 28 | 0.96 | 6.72 |
| 9 | AMT | dmdA | Pelagibacter ubique (HTCC1062) | 3tfhA | 45 | 45 | 1.00 | 7.85 |
| 10 | AMT | dmdA | Pelagibacter ubique (HTCC1062) | 3tfhA | 45 | 45 | 1.00 | 6.80 |

aIden1 is the percentage sequence identity of the templates in the threading aligned region with the query sequence

bIden2 is the percentage sequence identity of the whole template chains with query sequence 

cCov-Represents the coverage of the threading alignment and is equal to the number of aligned residues divided by the length of the query protein

dNorm. Z-score is the normalized Z-score of the threading alignments. Alignment with a normalized Z-score>1 means a good alignment and vice versa.

eAminomethyltransferase

fDmdA DMSP-dependent demethylase

gGlycine cleavage system T protein

hDimethylglycine dehydrogenase complexed with tetrahydrofolate 

Predicted model: C-sore=2; TM-score=0.99 dev= 0.04

| Rank | Class | Gene name | Organism | PDB ID | TM-scorea | RMSDb | IDENc | Covd |
| --- | --- | --- | --- | --- | --- | --- | --- | --- |
| 1 | AMT | dmdA | Pelagibacter ubique (HTCC1062) | 3tfhA | 0.997 | 0.39 | 0.443 | 1.000 |
| 2 | Oxidoreductase | dmgdH | Rattus norvegicus | 4p9sA | 0.932 | 1.79 | 0.194 | 0.986 |
| 3 | Oxidoreductase | dmge | Arthrobacter globiformis | 1pj6A2 | 0.915 | 2.18 | 0.258 | 0.986 |
| 4 | Oxidoreductase | dmg | Arthrobacter globiformis | 1pj7A | 0.914 | 2.18 | 0.258 | 0.986 |
| 5 | AMT | gcvT | Pyrococcus horikoshii | 1v5vA | 0.906 | 1.97 | 0.251 | 0.970 |
| 6 | AMT | gcvT | Thermotoga maritima | 1wooA | 0.899 | 1.74 | 0.250 | 0.951 |
| 7 | Oxidoreductase | soxAf | Stenotrophomonas maltophilia | 2gagA | 0.899 | 2.30 | 0.197 | 0.975 |
| 8 | Oxidoreductase | soxA | Corynebacterium sp. U-96 | 3ad7A | 0.899 | 2.36 | 0.202 | 0.978 |
| 9 | AMT | AMT(GCST) | Homo sapiens | 1wsrA | 0.891 | 2.07 | 0.259 | 0.959 |
| 10 | AMT | gcvT | Bartonella henselae | 3girA | 0.885 | 1.99 | 0.219 | 0.948 |

abIt is a standard for measuring structural similarity between two structures

cIt is the percentage sequence identity in the structurally aligned region 

dIt represents the coverage of the alignment by TM-align and is equal to the number of structurally aligned residues divided by length of the query protein

eDimethylglycine oxidase

fHeterotetrameric sarcosine oxidase alpha-subunit

Table 33. Up: Top ten analogs identified by LOMETS for threading alignments. Case of WP_053112834. In the middle: Model predicted by I-TASSER. Down: Top ten identified structural analogs in PDB by TM-align

| Rank | Class | Gene name | Organism | PDB ID | Iden1 (%)a | Iden2 (%)b | Covc | Norm. Z-scored |
| --- | --- | --- | --- | --- | --- | --- | --- | --- |
| 1 | AMTe | gcvT | Thermotoga maritima | 1wopA | 30 | 26 | 0.79 | 3.70 |
| 2 | AMT | gcvT | Pyrococcus horikoshii | 1v5vA | 23 | 23 | 0.81 | 4.82 |
| 3 | AMT | gcvT | Thermotoga maritima | 1worA | 29 | 26 | 0.79 | 4.52 |
| 4 | Oxidoreductase | dmgdH | Rattus norvegicus | 4p9s | 17 | 20 | 0.91 | 3.45 |
| 5 | Oxidoreductase | dmgdHh | Rattus norvegicus | 4p9sA | 18 | 20 | 0.82 | 2.36 |
| 6 | AMT | gcvT | Thermotoga maritima | 1wopA | 30 | 26 | 0.79 | 4.90 |
| 7 | Oxidoreductase | dmgdH | Rattus norvegicus | 4p9s | 18 | 20 | 0.85 | 3.59 |
| 8 | AMT | gcvTg | Bacillus subtilis | 1yx2A | 24 | 20 | 0.78 | 6.27 |
| 9 | AMT | dmdAf | Pelagibacter ubique (HTCC1062) | 3tfhA | 26 | 23 | 0.81 | 6.16 |
| 10 | AMT | dmdA | Pelagibacter ubique (HTCC1062) | 3tfhA | 26 | 23 | 0.81 | 5.91 |

aIden1 is the percentage sequence identity of the templates in the threading aligned region with the query sequence

bIden2 is the percentage sequence identity of the whole template chains with query sequence 

cCov-Represents the coverage of the threading alignment and is equal to the number of aligned residues divided by the length of the query protein

dNorm. Z-score is the normalized Z-score of the threading alignments. Alignment with a normalized Z-score>1 means a good alignment and vice versa.

eAminomethyltransferase

fDmdA DMSP-dependent demethylase

gGlycine cleavage system T protein

hDimethylglycine dehydrogenase complexed with tetrahydrofolate 

Predicted model: C-sore=0.56; TM-score=0.79 dev= 0.09

| Rank | Class | Gene name | Organism | PDB ID | TM-scorea | RMSDb | IDENc | Covd |
| --- | --- | --- | --- | --- | --- | --- | --- | --- |
| 1 | Oxidoreductase | dmgdH | Rattus norvegicus | 4p9sA | 0.997 | 0.39 | 0.443 | 1.000 |
| 2 | Oxidoreductase | dmge | Arthrobacter globiformis | 1pj6A | 0.932 | 1.79 | 0.194 | 0.986 |
| 3 | AMT | gcvT | Pyrococcus horikoshii | 1v5vA | 0.915 | 2.18 | 0.258 | 0.986 |
| 4 | AMT | gcvT | Thermotoga maritima | 1worA | 0.914 | 2.18 | 0.258 | 0.986 |
| 5 | Oxidoreductase | soxAf | Stenotrophomonas maltophilia | 2gagA | 0.906 | 1.97 | 0.251 | 0.970 |
| 6 | Oxidoreductase | soxA | Corynebacterium sp. U-96 | 1vrqA | 0.899 | 1.74 | 0.250 | 0.951 |
| 7 | AMT | AMT(GCST) | Homo sapiens | 1wsvB | 0.899 | 2.30 | 0.197 | 0.975 |
| 8 | AMT | dmdA | Pelagibacter ubique (HTCC1062) | 3tfiA | 0.899 | 2.36 | 0.202 | 0.978 |
| 9 | AMT | gcvT | Bartonella henselae | 3girA | 0.891 | 2.07 | 0.259 | 0.959 |
| 10 | AMT | gcvT | Bacillus subtilis | 1yx2B | 0.885 | 1.99 | 0.219 | 0.948 |

abIt is a standard for measuring structural similarity between two structures

cIt is the percentage sequence identity in the structurally aligned region 

dIt represents the coverage of the alignment by TM-align and is equal to the number of structurally aligned residues divided by length of the query protein

eDimethylglycine oxidase

fHeterotetrameric sarcosine oxidase alpha-subunit

Table 34. Up: Top ten analogs identified by LOMETS for threading alignments. Case of WP_053819980_PS1. In the middle: Model predicted by I-TASSER. Down: Top ten identified structural analogs in PDB by TM-align

| Rank | Class | Gene name | Organism | PDB ID | Iden1 (%)a | Iden2 (%)b | Covc | Norm. Z-scored |
| --- | --- | --- | --- | --- | --- | --- | --- | --- |
| 1 | AMTe | dmdAf | Pelagibacter ubique (HTCC1062) | 3tfhA | 40 | 40 | 0.99 | 4.74 |
| 2 | AMT | dmdA | Pelagibacter ubique (HTCC1062) | 3tfhA | 40 | 40 | 0.99 | 5.36 |
| 3 | AMT | dmdA | Pelagibacter ubique (HTCC1062) | 3tfhA | 40 | 40 | 0.99 | 5.62 |
| 4 | AMT | dmdA | Pelagibacter ubique (HTCC1062) | 3tfh | 40 | 40 | 0.99 | 3.23 |
| 5 | Oxidoreductase | dmgdHh | Rattus norvegicus | 4p9sA | 20 | 24 | 0.98 | 2.35 |
| 6 | AMT | dmdA | Pelagibacter ubique (HTCC1062) | 3tfhA | 39 | 40 | 0.99 | 5.74 |
| 7 | AMT | dmdA | Pelagibacter ubique (HTCC1062) | 3tfh | 39 | 40 | 0.98 | 3.55 |
| 8 | AMT | gcvTg | Bacillus subtilis | 1yx2A | 24 | 26 | 0.95 | 6.47 |
| 9 | AMT | dmdA | Pelagibacter ubique (HTCC1062) | 3tfhA | 41 | 40 | 0.99 | 8.01 |
| 10 | AMT | dmdA | Pelagibacter ubique (HTCC1062) | 3tfhA | 40 | 40 | 0.99 | 6.81 |

aIden1 is the percentage sequence identity of the templates in the threading aligned region with the query sequence

bIden2 is the percentage sequence identity of the whole template chains with query sequence 

cCov-Represents the coverage of the threading alignment and is equal to the number of aligned residues divided by the length of the query protein

dNorm. Z-score is the normalized Z-score of the threading alignments. Alignment with a normalized Z-score>1 means a good alignment and vice versa.

eAminomethyltransferase

fDmdA DMSP-dependent demethylase

gGlycine cleavage system T protein

hDimethylglycine dehydrogenase complexed with tetrahydrofolate 

Predicted model: C-sore=1.71; TM-score=0.95 dev= 0.05

| Rank | Class | Gene name | Organism | PDB ID | TM-scorea | RMSDb | IDENc | Covd |
| --- | --- | --- | --- | --- | --- | --- | --- | --- |
| 1 | AMT | dmdA | Pelagibacter ubique (HTCC1062) | 3tfhA | 0.988 | 0.60 | 0.404 | 0.995 |
| 2 | Oxidoreductase | dmgdH | Rattus norvegicus | 4p9sA | 0.924 | 1.89 | 0.214 | 0.981 |
| 3 | Oxidoreductase | dmge | Arthrobacter globiformis | 1pj6A | 0.907 | 2.26 | 0.247 | 0.981 |
| 4 | AMT | gcvT | Pyrococcus horikoshii | 1v5vA | 0.894 | 1.97 | 0.254 | 0.957 |
| 5 | Oxidoreductase | soxAf | Corynebacterium sp. U-96 | 3ad7A | 0.890 | 2.41 | 0.216 | 0.968 |
| 6 | Oxidoreductase | soxA | Stenotrophomonas maltophilia | 2gagA | 0.890 | 2.30 | 0.219 | 0.965 |
| 7 | AMT | gcvT | Thermotoga maritima | 1wooA | 0.887 | 1.83 | 0.246 | 0.941 |
| 8 | AMT | AMT(GCST) | Homo sapiens | 1wsrA | 0.879 | 2.13 | 0.236 | 0.949 |
| 9 | AMT | gcvT | Bartonella henselae | 3girA | 0.876 | 1.95 | 0.231 | 0.935 |
| 10 | AMT | gcvT | Bacillus subtilis | 1yx2B | 0.868 | 2.18 | 0.246 | 0.941 |

abIt is a standard for measuring structural similarity between two structures

cIt is the percentage sequence identity in the structurally aligned region 

dIt represents the coverage of the alignment by TM-align and is equal to the number of structurally aligned residues divided by length of the query protein

eDimethylglycine oxidase

fHeterotetrameric sarcosine oxidase alpha-subunit

Table 35. Up: Top ten analogs identified by LOMETS for threading alignments. Case of WP_065273401.1. In the middle: Model predicted by I-TASSER. Down: Top ten identified structural analogs in PDB by TM-align

| Rank | Class | Gene name | Organism | PDB ID | Iden1 (%)a | Iden2 (%)b | Covc | Norm. Z-scored |
| --- | --- | --- | --- | --- | --- | --- | --- | --- |
| 1 | AMTe | dmdAf | Pelagibacter ubique (HTCC1062) | 3tfhA | 45 | 44 | 1.00 | 4.79 |
| 2 | AMT | dmdA | Pelagibacter ubique (HTCC1062) | 3tfhA | 44 | 44 | 1.00 | 5.36 |
| 3 | AMT | dmdA | Pelagibacter ubique (HTCC1062) | 3tfhA | 44 | 44 | 1.00 | 5.69 |
| 4 | AMT | dmdA | Pelagibacter ubique (HTCC1062) | 3tfh | 45 | 44 | 0.99 | 3.19 |
| 5 | Oxidoreductase | dmgdHh | Rattus norvegicus | 4p9sA | 17 | 20 | 0.98 | 2.36 |
| 6 | AMT | dmdA | Pelagibacter ubique (HTCC1062) | 3tfhA | 44 | 44 | 1.00 | 5.81 |
| 7 | AMT | dmdA | Pelagibacter ubique (HTCC1062) | 3tfh | 44 | 44 | 0.98 | 3.53 |
| 8 | AMT | gcvTg | Bacillus subtilis | 1yx2A | 24 | 26 | 0.96 | 6.64 |
| 9 | AMT | dmdA | Pelagibacter ubique (HTCC1062) | 3tfhA | 44 | 44 | 1.00 | 7.77 |
| 10 | AMT | dmdA | Pelagibacter ubique (HTCC1062) | 3tfhA | 44 | 44 | 1.00 | 6.76 |

aIden1 is the percentage sequence identity of the templates in the threading aligned region with the query sequence

bIden2 is the percentage sequence identity of the whole template chains with query sequence 

cCov-Represents the coverage of the threading alignment and is equal to the number of aligned residues divided by the length of the query protein

dNorm. Z-score is the normalized Z-score of the threading alignments. Alignment with a normalized Z-score>1 means a good alignment and vice versa.

eAminomethyltransferase

fDmdA DMSP-dependent demethylase

gGlycine cleavage system T protein

hDimethylglycine dehydrogenase complexed with tetrahydrofolate 

Predicted model: C-sore=2; TM-score=0.99 dev= 0.04

| Rank | Class | Gene name | Organism | PDB ID | TM-scorea | RMSDb | IDENc | Covd |
| --- | --- | --- | --- | --- | --- | --- | --- | --- |
| 1 | AMT | dmdA | Pelagibacter ubique (HTCC1062) | 3tfhA | 0.997 | 0.39 | 0.439 | 1.00 |
| 2 | Oxidoreductase | dmgdH | Rattus norvegicus | 4p9sA | 0.929 | 1.81 | 0.180 | 0.984 |
| 3 | Oxidoreductase | dmge | Arthrobacter globiformis | 1pj6A | 0.913 | 2.17 | 0.249 | 0.984 |
| 4 | Oxidoreductase | dmg | Arthrobacter globiformis | 1pj7A | 0.913 | 2.17 | 0.249 | 0.984 |
| 5 | AMT | gcvT | Pyrococcus horikoshii | 1v5vA | 0.904 | 1.96 | 0.223 | 0.967 |
| 6 | Oxidoreductase | soxAf | Stenotrophomonas maltophilia | 2gagA | 0.899 | 2.31 | 0.199 | 0.976 |
| 7 | Oxidoreductase | soxA | Corynebacterium sp. U-96 | 3ad7A | 0.899 | 2.28 | 0.182 | 0.976 |
| 8 | AMT | gcvT | Thermotoga maritima | 1wooA | 0.897 | 1.76 | 0.227 | 0.948 |
| 9 | AMT | AMT(GCST) | Homo sapiens | 1wsrA | 0.890 | 2.10 | 0.230 | 0.959 |
| 10 | AMT | gcvT | Bartonella henselae | 3girA | 0.885 | 1.94 | 0.228 | 0.946 |

abIt is a standard for measuring structural similarity between two structures

cIt is the percentage sequence identity in the structurally aligned region 

dIt represents the coverage of the alignment by TM-align and is equal to the number of structurally aligned residues divided by length of the query protein

eDimethylglycine oxidase

fHeterotetrameric sarcosine oxidase alpha-subunit

Table 36. Up: Top ten analogs identified by LOMETS for threading alignments. Case of WP_071941841.1. In the middle: Model predicted by I-TASSER. Down: Top ten identified structural analogs in PDB by TM-align

| Rank | Class | Gene name | Organism | PDB ID | Iden1 (%)a | Iden2 (%)b | Covc | Norm. Z-scored |
| --- | --- | --- | --- | --- | --- | --- | --- | --- |
| 1 | AMTe | dmdAf | Pelagibacter ubique (HTCC1062) | 3tfhA | 33 | 32 | 0.92 | 4.23 |
| 2 | AMT | gcvT | Pyrococcus horikoshii | 1v5vA | 27 | 28 | 0.91 | 5.28 |
| 3 | AMT | dmdA | Pelagibacter ubique (HTCC1062) | 3tfhA | 34 | 32 | 0.92 | 5.23 |
| 4 | Oxidoreductase | dmgdH | Rattus norvegicus | 4p9s | 24 | 25 | 0.95 | 3.20 |
| 5 | Oxidoreductase | dmgdHh | Rattus norvegicus | 4p9sA | 21 | 25 | 0.93 | 2.34 |
| 6 | AMT | dmdA | Pelagibacter ubique (HTCC1062) | 3tfhA | 33 | 32 | 0.92 | 5.36 |
| 7 | Oxidoreductase | dmg | Arthrobacter globiformis | 1pj6 | 23 | 27 | 0.94 | 3.53 |
| 8 | AMT | gcvTg | Bacillus subtilis | 1yx2A | 29 | 28 | 0.89 | 6.40 |
| 9 | AMT | dmdA | Pelagibacter ubique (HTCC1062) | 3tfhA | 34 | 32 | 0.92 | 6.94 |
| 10 | AMT | dmdA | Pelagibacter ubique (HTCC1062) | 3tfhA | 34 | 32 | 0.92 | 6.35 |

aIden1 is the percentage sequence identity of the templates in the threading aligned region with the query sequence

bIden2 is the percentage sequence identity of the whole template chains with query sequence 

cCov-Represents the coverage of the threading alignment and is equal to the number of aligned residues divided by the length of the query protein

dNorm. Z-score is the normalized Z-score of the threading alignments. Alignment with a normalized Z-score>1 means a good alignment and vice versa.

eAminomethyltransferase

fDmdA DMSP-dependent demethylase

gGlycine cleavage system T protein

hDimethylglycine dehydrogenase complexed with tetrahydrofolate 

Predicted model: C-sore=1.11; TM-score=0.87 dev= 0.07

| Rank | Class | Gene name | Organism | PDB ID | TM-scorea | RMSDb | IDENc | Covd |
| --- | --- | --- | --- | --- | --- | --- | --- | --- |
| 1 | Oxidoreductase | dmgdH | Rattus norvegicus | 4p9sA | 0.997 | 0.39 | 0.439 | 1.00 |
| 2 | AMT | dmdA | Pelagibacter ubique HTCC1062 | 3tfiA | 0.929 | 1.81 | 0.180 | 0.984 |
| 3 | Oxidoreductase | dmge | Arthrobacter globiformis | 1pj6A | 0.913 | 2.17 | 0.249 | 0.984 |
| 4 | AMT | gcvT | Pyrococcus horikoshii | 1v5vA | 0.913 | 2.17 | 0.249 | 0.984 |
| 5 | Oxidoreductase | soxAf | Stenotrophomonas maltophilia | 2gagA | 0.904 | 1.96 | 0.223 | 0.967 |
| 6 | Oxidoreductase | soxA | Corynebacterium sp. U-96 | 1vrqA | 0.899 | 2.31 | 0.199 | 0.976 |
| 7 | AMT | gcvT | Thermotoga maritima | 1worA | 0.899 | 2.28 | 0.182 | 0.976 |
| 8 | AMT | AMT(GCST) | Homo sapiens | 1wsrA | 0.897 | 1.76 | 0.227 | 0.948 |
| 9 | AMT | gcvT | Bartonella henselae | 3girA | 0.890 | 2.10 | 0.230 | 0.959 |
| 10 | AMT | gcvT | Bacillus subtilis | 1yx2B | 0.885 | 1.94 | 0.228 | 0.946 |

abIt is a standard for measuring structural similarity between two structures

cIt is the percentage sequence identity in the structurally aligned region 

dIt represents the coverage of the alignment by TM-align and is equal to the number of structurally aligned residues divided by length of the query protein

eDimethylglycine oxidase

fHeterotetrameric sarcosine oxidase alpha-subunit

Table 37. Up: Top ten analogs identified by LOMETS for threading alignments. Case of WP_076627280.1. In the middle: Model predicted by I-TASSER. Down: Top ten identified structural analogs in PDB by TM-align

| Rank | Class | Gene name | Organism | PDB ID | Iden1 (%)a | Iden2 (%)b | Covc | Norm. Z-scored |
| --- | --- | --- | --- | --- | --- | --- | --- | --- |
| 1 | AMTe | dmdAf | Pelagibacter ubique (HTCC1062) | 3tfhA | 42 | 43 | 1.00 | 4.70 |
| 2 | AMT | dmdA | Pelagibacter ubique (HTCC1062) | 3tfhA | 43 | 43 | 1.00 | 5.33 |
| 3 | AMT | dmdA | Pelagibacter ubique (HTCC1062) | 3tfhA | 42 | 43 | 1.00 | 5.69 |
| 4 | AMT | dmdA | Pelagibacter ubique (HTCC1062) | 3tfh | 44 | 43 | 0.99 | 3.19 |
| 5 | Oxidoreductase | dmgdHh | Rattus norvegicus | 4p9sA | 18 | 21 | 0.98 | 2.36 |
| 6 | AMT | dmdA | Pelagibacter ubique (HTCC1062) | 3tfhA | 41 | 43 | 0.99 | 5.82 |
| 7 | AMT | dmdA | Pelagibacter ubique (HTCC1062) | 3tfh | 41 | 43 | 0.98 | 3.53 |
| 8 | AMT | gcvTg | Bacillus subtilis | 1yx2A | 25 | 27 | 0.96 | 6.61 |
| 9 | AMT | dmdA | Pelagibacter ubique (HTCC1062) | 3tfhA | 43 | 43 | 1.00 | 7.43 |
| 10 | AMT | dmdA | Pelagibacter ubique (HTCC1062) | 3tfhA | 43 | 43 | 1.00 | 6.73 |

aIden1 is the percentage sequence identity of the templates in the threading aligned region with the query sequence

bIden2 is the percentage sequence identity of the whole template chains with query sequence 

cCov-Represents the coverage of the threading alignment and is equal to the number of aligned residues divided by the length of the query protein

dNorm. Z-score is the normalized Z-score of the threading alignments. Alignment with a normalized Z-score>1 means a good alignment and vice versa.

eAminomethyltransferase

fDmdA DMSP-dependent demethylase

gGlycine cleavage system T protein

hDimethylglycine dehydrogenase complexed with tetrahydrofolate 

Predicted model: C-sore=2; TM-score=0.99 dev= 0.04

| Rank | Class | Gene name | Organism | PDB ID | TM-scorea | RMSDb | IDENc | Covd |
| --- | --- | --- | --- | --- | --- | --- | --- | --- |
| 1 | AMT | dmdA | Pelagibacter ubique HTCC1062 | 3tfhA | 0.997 | 0.38 | 0.428 | 1.00 |
| 2 | Oxidoreductase | dmgdh | Rattus norvegicus | 4p9sA | 0.930 | 1.79 | 0.183 | 0.984 |
| 3 | Oxidoreductase | dmge | Arthrobacter globiformis | 1pj6A2 | 0.913 | 2.16 | 0.255 | 0.984 |
| 4 | Oxidoreductase | dmg | Arthrobacter globiformis | 1pj7A | 0.913 | 2.17 | 0.255 | 0.984 |
| 5 | AMT | gcvT | Pyrococcus horikoshii | 1v5vA | 0.905 | 1.94 | 0.223 | 0.967 |
| 6 | Oxidoreductase | soxAf | Stenotrophomonas maltophilia | 2gagA | 0.900 | 2.27 | 0.188 | 0.976 |
| 7 | Oxidoreductase | soxA | Corynebacterium sp. U-96 | 3ad7A | 0.899 | 2.27 | 0.182 | 0.976 |
| 8 | AMT | gcvT | Thermotoga maritima | 1wooA | 0.897 | 1.74 | 0.239 | 0.946 |
| 9 | AMT | AMT(GCST) | Homo sapiens | 1wsrA | 0.891 | 2.08 | 0.264 | 0.959 |
| 10 | AMT | gcvT | Bartonella henselae | 3girA | 0.886 | 1.92 | 0.239 | 0.946 |

abIt is a standard for measuring structural similarity between two structures

cIt is the percentage sequence identity in the structurally aligned region 

dIt represents the coverage of the alignment by TM-align and is equal to the number of structurally aligned residues divided by length of the query protein

eDimethylglycine oxidase

fHeterotetrameric sarcosine oxidase alpha-subunit

Table 38. Up: Top ten analogs identified by LOMETS for threading alignments. Case of WP_096389816.1. In the middle: Model predicted by I-TASSER. Down: Top ten identified structural analogs in PDB by TM-align

| Rank | Class | Gene name | Organism | PDB ID | Iden1 (%)a | Iden2 (%)b | Covc | Norm. Z-scored |
| --- | --- | --- | --- | --- | --- | --- | --- | --- |
| 1 | AMTe | gcvT | Thermotoga maritima | 1wopA | 30 | 27 | 0.79 | 3.58 |
| 2 | AMT | gcvT | Pyrococcus horikoshii | 1v5vA | 27 | 25 | 0.81 | 4.94 |
| 3 | AMT | gcvT | Rhermotoga maritima | 1worA | 31 | 27 | 0.79 | 4.63 |
| 4 | Oxidoreductase | dmgdH | Rattus norvegicus | 4p9s | 18 | 20 | 0.89 | 3.35 |
| 5 | Oxidoreductase | dmgdHh | Rattus norvegicus | 4p9sA | 18 | 20 | 0.84 | 2.38 |
| 6 | AMT | gcvT | Thermotoga maritima | 1wopA | 30 | 27 | 0.79 | 4.90 |
| 7 | Oxidoreductase | dmgdH | Rattus norvegicus | 4p9s | 18 | 20 | 0.84 | 3.57 |
| 8 | AMT | gcvTg | Bacillus subtilis | 1yx2A | 26 | 23 | 0.78 | 5.96 |
| 9 | AMT | dmdAf | Pelagibacter ubique (HTCC1062) | 3tfhA | 27 | 23 | 0.82 | 5.78 |
| 10 | AMT | dmdA | Pelagibacter ubique (HTCC1062) | 3tfhA | 28 | 23 | 0.82 | 5.76 |

aIden1 is the percentage sequence identity of the templates in the threading aligned region with the query sequence

bIden2 is the percentage sequence identity of the whole template chains with query sequence 

cCov-Represents the coverage of the threading alignment and is equal to the number of aligned residues divided by the length of the query protein

dNorm. Z-score is the normalized Z-score of the threading alignments. Alignment with a normalized Z-score>1 means a good alignment and vice versa.

eAminomethyltransferase

fDmdA DMSP-dependent demethylase

gGlycine cleavage system T protein

hDimethylglycine dehydrogenase complexed with tetrahydrofolate 

Predicted model: C-sore=0.48; TM-score=0.78 dev= 0.10

| Rank | Class | Gene name | Organism | PDB ID | TM-scorea | RMSDb | IDENc | Covd |
| --- | --- | --- | --- | --- | --- | --- | --- | --- |
| 1 | Oxidoreductase | dmgdH | Rattus norvegicus | 4p9sA | 0.885 | 1.02 | 0.170 | 0.900 |
| 2 | Oxidoreductase | dmge | Arthrobacter globiformis | 1pj6A | 0.848 | 2.00 | 0.216 | 0.894 |
| 3 | Oxidoreductase | soxAf | Stenotrophomonas maltophilia | 2gagA | 0.790 | 2.68 | 0.198 | 0.854 |
| 4 | Oxidoreductase | soxA | Corynebacterium sp. U-96 | 1x31A | 0.787 | 2.67 | 0.193 | 0.851 |
| 5 | AMT | gcvT | Thermotoga maritima | 1wooA | 0.777 | 1.07 | 0.297 | 0.792 |
| 6 | AMT | gcvT | Pyrococcus horikoshii | 1v5vA | 0.777 | 1.69 | 0.252 | 0.809 |
| 7 | AMT | AMT(GCST) | Homo sapiens | 1wsvB | 0.766 | 1.56 | 0.229 | 0.794 |
| 8 | AMT | dmdA | Pelagibacter ubique HTCC1062 | 3tfiA | 0.762 | 2.09 | 0.254 | 0.811 |
| 9 | AMT | gcvT | Bartonella henselae | 3girA | 0.752 | 1.74 | 0.220 | 0.785 |
| 10 | AMT | gcvT | Bacillus subtilis | 1yx2B | 0.750 | 1.58 | 0.251 | 0.778 |

abIt is a standard for measuring structural similarity between two structures

cIt is the percentage sequence identity in the structurally aligned region 

dIt represents the coverage of the alignment by TM-align and is equal to the number of structurally aligned residues divided by length of the query protein

eDimethylglycine oxidase

fHeterotetrameric sarcosine oxidase alpha-subunit

Table 39. Up: Top ten analogs identified by LOMETS for threading alignments. Case of ABF64177.1. In the middle: Model predicted by I-TASSER. Down: Top ten identified structural analogs in PDB by TM-align

| Rank | Class | Gene name | Organism | PDB ID | Iden1 (%)a | Iden2 (%)b | Covc | Norm. Z-scored |
| --- | --- | --- | --- | --- | --- | --- | --- | --- |
| 1 | AMTe | dmdAf | Pelagibacter ubique (HTCC1062) | 3tfhA | 44 | 42 | 0.96 | 4.59 |
| 2 | AMT | dmdA | Pelagibacter ubique (HTCC1062) | 3tfhA | 44 | 42 | 0.95 | 5.21 |
| 3 | AMT | dmdA | Pelagibacter ubique (HTCC1062) | 3tfhA | 44 | 42 | 0.96 | 5.55 |
| 4 | Oxidoreductase | dmgdH | Rattus norvegicus | 4p9s | 17 | 22 | 0.97 | 3.28 |
| 5 | Oxidoreductase | dmgdHh | Rattus norvegicus | 4p9sA | 18 | 21 | 0.94 | 2.38 |
| 6 | AMT | dmdA | Pelagibacter ubique (HTCC1062) | 3tfhA | 44 | 42 | 0.95 | 5.58 |
| 7 | Oxidoreductase | dmgdH | Rattus norvegicus | 4p9s | 17 | 22 | 0.97 | 3.63 |
| 8 | AMT | gcvTg | Bacillus subtilis | 1yx2A | 25 | 26 | 0.91 | 6.61 |
| 9 | AMT | dmdA | Pelagibacter ubique (HTCC1062) | 3tfhA | 44 | 42 | 0.96 | 7.88 |
| 10 | AMT | dmdA | Pelagibacter ubique (HTCC1062) | 3tfhA | 44 | 42 | 0.95 | 11.88 |

aIden1 is the percentage sequence identity of the templates in the threading aligned region with the query sequence

bIden2 is the percentage sequence identity of the whole template chains with query sequence 

cCov-Represents the coverage of the threading alignment and is equal to the number of aligned residues divided by the length of the query protein

dNorm. Z-score is the normalized Z-score of the threading alignments. Alignment with a normalized Z-score>1 means a good alignment and vice versa.

eAminomethyltransferase

fDmdA DMSP-dependent demethylase

gGlycine cleavage system T protein

hDimethylglycine dehydrogenase complexed with tetrahydrofolate 

Predicted model: C-sore=1.62; TM-score=0.94 dev= 0.05

| Rank | Class | Gene name | Organism | PDB ID | TM-scorea | RMSDb | IDENc | Covd |
| --- | --- | --- | --- | --- | --- | --- | --- | --- |
| 1 | AMT | dmdA | Pelagibacter ubique HTCC1062 | 3tfiA | 0.947 | 0.85 | 0.436 | 0.958 |
| 2 | Oxidoreductase | dmgdH | Rattus norvegicus | 4p9sA | 0.939 | 1.62 | 0.175 | 0.982 |
| 3 | Oxidoreductase | dmge | Arthrobacter globiformis | 1pj6A | 0.921 | 2.06 | 0.246 | 0.982 |
| 4 | AMT | gcvT | Pyrococcus horikoshii | 1v5vA | 0.870 | 1.84 | 0.228 | 0.922 |
| 5 | Oxidoreductase | soxAf | Stenotrophomonas maltophilia | 2gagA | 0.866 | 2.29 | 0.192 | 0.932 |
| 6 | Oxidoreductase | soxA | Corynebacterium sp. U-96 | 3ad7A | 0.865 | 2.37 | 0.184 | 0.935 |
| 7 | AMT | gcvT | Thermotoga maritima | 1wooA | 0.864 | 1.60 | 0.247 | 0.904 |
| 8 | AMT | AMT(GCST) | Homo sapiens | 1wsvA | 0.858 | 1.96 | 0.256 | 0.914 |
| 9 | AMT | gcvT | Bartonella henselae | 3girA | 0.849 | 1.88 | 0.242 | 0.901 |
| 10 | AMT | gcvT | Bacillus subtilis | 1yx2B | 0.846 | 2.05 | 0.252 | 0.906 |

abIt is a standard for measuring structural similarity between two structures

cIt is the percentage sequence identity in the structurally aligned region 

dIt represents the coverage of the alignment by TM-align and is equal to the number of structurally aligned residues divided by length of the query protein

eDimethylglycine oxidase

fHeterotetrameric sarcosine oxidase alpha-subunit

Table 40. Up: Top ten analogs identified by LOMETS for threading alignments. Case of AEI94210.1. In the middle: Model predicted by I-TASSER. Down: Top ten identified structural analogs in PDB by TM-align

| Rank | Class | Gene name | Organism | PDB ID | Iden1 (%)a | Iden2 (%)b | Covc | Norm. Z-scored |
| --- | --- | --- | --- | --- | --- | --- | --- | --- |
| 1 | AMTe | dmdAf | Pelagibacter ubique (HTCC1062) | 3tfhA | 44 | 44 | 1.00 | 4.83 |
| 2 | AMT | dmdA | Pelagibacter ubique (HTCC1062) | 3tfhA | 44 | 44 | 1.00 | 5.36 |
| 3 | AMT | dmdA | Pelagibacter ubique (HTCC1062) | 3tfhA | 44 | 44 | 1.00 | 5.76 |
| 4 | AMT | dmdA | Pelagibacter ubique (HTCC1062) | 3tfh | 44 | 44 | 0.99 | 3.17 |
| 5 | Oxidoreductase | dmgdHh | Rattus norvegicus | 4p9sA | 16 | 23 | 0.98 | 2.36 |
| 6 | AMT | dmdA | Pelagibacter ubique (HTCC1062) | 3tfhA | 44 | 44 | 1.00 | 5.86 |
| 7 | AMT | dmdA | Pelagibacter ubique (HTCC1062) | 3tfh | 42 | 44 | 0.98 | 3.53 |
| 8 | AMT | gcvTg | Bacillus subtilis | 1yx2A | 24 | 26 | 0.96 | 6.58 |
| 9 | AMT | dmdA | Pelagibacter ubique (HTCC1062) | 3tfhA | 44 | 44 | 1.00 | 7.87 |
| 10 | AMT | dmdA | Pelagibacter ubique (HTCC1062) | 3tfhA | 44 | 44 | 1.00 | 6.74 |

aIden1 is the percentage sequence identity of the templates in the threading aligned region with the query sequence

bIden2 is the percentage sequence identity of the whole template chains with query sequence 

cCov-Represents the coverage of the threading alignment and is equal to the number of aligned residues divided by the length of the query protein

dNorm. Z-score is the normalized Z-score of the threading alignments. Alignment with a normalized Z-score>1 means a good alignment and vice versa.

eAminomethyltransferase

fDmdA DMSP-dependent demethylase

gGlycine cleavage system T protein

hDimethylglycine dehydrogenase complexed with tetrahydrofolate 

Predicted model: C-sore=2; TM-score=0.99 dev= 0.04

| Rank | Class | Gene name | Organism | PDB ID | TM-scorea | RMSDb | IDENc | Covd |
| --- | --- | --- | --- | --- | --- | --- | --- | --- |
| 1 | AMT | dmdA | Pelagibacter ubique HTCC1062 | 3tfhA | 0.997 | 0.41 | 0.436 | 1.000 |
| 2 | Oxidoreductase | dmgdH | Rattus norvegicus | 4p9sA | 0.930 | 1.80 | 0.175 | 0.984 |
| 3 | Oxidoreductase | dmge | Arthrobacter globiformis | 1pj6A2 | 0.913 | 2.17 | 0.249 | 0.984 |
| 4 | Oxidoreductase | dmg | Arthrobacter globiformis | 1pj7A | 0.912 | 2.18 | 0.249 | 0.984 |
| 5 | AMT | gcvT | Pyrococcus horikoshii | 1v5vA | 0.907 | 1.92 | 0.231 | 0.967 |
| 6 | Oxidoreductase | soxAf | Stenotrophomonas maltophilia | 2gagA | 0.901 | 2.28 | 0.191 | 0.976 |
| 7 | Oxidoreductase | soxA | Corynebacterium sp. U-96 | 3ad7A | 0.900 | 2.26 | 0.191 | 0.976 |
| 8 | AMT | gcvT | Thermotoga maritima | 1worA | 0.898 | 1.79 | 0.255 | 0.951 |
| 9 | AMT | AMT(GCST) | Homo sapiens | 1wsrA | 0.892 | 2.07 | 0.259 | 0.959 |
| 10 | AMT | gcvT | Bartonella henselae | 3girA | 0.886 | 1.93 | 0.225 | 0.946 |

abIt is a standard for measuring structural similarity between two structures

cIt is the percentage sequence identity in the structurally aligned region 

dIt represents the coverage of the alignment by TM-align and is equal to the number of structurally aligned residues divided by length of the query protein

eDimethylglycine oxidase

fHeterotetrameric sarcosine oxidase alpha-subunit

Table 41. Up: Top ten analogs identified by LOMETS for threading alignments. Case of ABG31871. In the middle: Model predicted by I-TASSER. Down: Top ten identified structural analogs in PDB by TM-align

| Rank | Class | Gene name | Organism | PDB ID | Iden1 (%)a | Iden2 (%)b | Covc | Norm. Z-scored |
| --- | --- | --- | --- | --- | --- | --- | --- | --- |
| 1 | AMTe | dmdAf | Pelagibacter ubique (HTCC1062) | 3tfhA | 44 | 44 | 1.00 | 4.86 |
| 2 | AMT | dmdA | Pelagibacter ubique (HTCC1062) | 3tfhA | 44 | 44 | 1.00 | 5.39 |
| 3 | AMT | dmdA | Pelagibacter ubique (HTCC1062) | 3tfhA | 44 | 44 | 1.00 | 5.80 |
| 4 | AMT | dmdA | Pelagibacter ubique (HTCC1062) | 3tfh | 44 | 44 | 0.99 | 3.18 |
| 5 | Oxidoreductase | dmgdHh | Rattus norvegicus | 4p9sA | 16 | 22 | 0.98 | 2.36 |
| 6 | AMT | dmdA | Pelagibacter ubique (HTCC1062) | 3tfhA | 44 | 44 | 1.00 | 5.93 |
| 7 | AMT | dmdA | Pelagibacter ubique (HTCC1062) | 3tfh | 44 | 44 | 0.98 | 3.53 |
| 8 | AMT | gcvTg | Bacillus subtilis | 1yx2A | 24 | 26 | 0.96 | 6.66 |
| 9 | AMT | dmdA | Pelagibacter ubique (HTCC1062) | 3tfhA | 44 | 44 | 1.00 | 7.94 |
| 10 | AMT | dmdA | Pelagibacter ubique (HTCC1062) | 3tfhA | 44 | 44 | 1.00 | 6.82 |

aIden1 is the percentage sequence identity of the templates in the threading aligned region with the query sequence

bIden2 is the percentage sequence identity of the whole template chains with query sequence 

cCov-Represents the coverage of the threading alignment and is equal to the number of aligned residues divided by the length of the query protein

dNorm. Z-score is the normalized Z-score of the threading alignments. Alignment with a normalized Z-score>1 means a good alignment and vice versa.

eAminomethyltransferase

fDmdA DMSP-dependent demethylase

gGlycine cleavage system T protein

hDimethylglycine dehydrogenase complexed with tetrahydrofolate 

Predicted model: C-sore=2; TM-score=0.99 dev= 0.04

| Rank | Class | Gene name | Organism | PDB ID | TM-scorea | RMSDb | IDENc | Covd |
| --- | --- | --- | --- | --- | --- | --- | --- | --- |
| 1 | AMT | dmdA | Pelagibacter ubique HTCC1062 | 3tfhA | 0.997 | 0.41 | 0.436 | 1.000 |
| 2 | Oxidoreductase | dmgdH | Rattus norvegicus | 4p9sA | 0.931 | 1.79 | 0.175 | 0.984 |
| 3 | Oxidoreductase | dmge | Arthrobacter globiformis | 1pj6A2 | 0.914 | 2.16 | 0.258 | 0.984 |
| 4 | Oxidoreductase | dmg | Arthrobacter globiformis | 1pj7A | 0.913 | 2.18 | 0.258 | 0.984 |
| 5 | AMT | gcvT | Pyrococcus horikoshii | 1v5vA | 0.905 | 1.94 | 0.237 | 0.967 |
| 6 | Oxidoreductase | soxAf | Stenotrophomonas maltophilia | 2gagA | 0.900 | 2.25 | 0.202 | 0.976 |
| 7 | Oxidoreductase | soxA | Corynebacterium sp. U-96 | 3ad7A | 0.900 | 2.26 | 0.196 | 0.976 |
| 8 | AMT | gcvT | Thermotoga maritima | 1wooA | 0.898 | 1.73 | 0.261 | 0.948 |
| 9 | AMT | AMT(GCST) | Homo sapiens | 1wsrA | 0.892 | 2.07 | 0.267 | 0.959 |
| 10 | AMT | gcvT | Bartonella henselae | 3girA | 0.887 | 1.92 | 0.228 | 0.946 |

abIt is a standard for measuring structural similarity between two structures

cIt is the percentage sequence identity in the structurally aligned region 

dIt represents the coverage of the alignment by TM-align and is equal to the number of structurally aligned residues divided by length of the query protein

eDimethylglycine oxidase

fHeterotetrameric sarcosine oxidase alpha-subunit

Table 42. Up: Top ten analogs identified by LOMETS for threading alignments. Case of ABD55296.1. In the middle: Model predicted by I-TASSER. Down: Top ten identified structural analogs in PDB by TM-align

| Rank | Class | Gene name | Organism | PDB ID | Iden1 (%)a | Iden2 (%)b | Covc | Norm. Z-scored |
| --- | --- | --- | --- | --- | --- | --- | --- | --- |
| 1 | AMTe | dmdAf | Pelagibacter ubique (HTCC1062) | 3tfhA | 43 | 44 | 1.00 | 4.70 |
| 2 | AMT | dmdA | Pelagibacter ubique (HTCC1062) | 3tfhA | 44 | 44 | 1.00 | 5.33 |
| 3 | AMT | dmdA | Pelagibacter ubique (HTCC1062) | 3tfhA | 43 | 44 | 1.00 | 5.76 |
| 4 | AMT | dmdA | Pelagibacter ubique (HTCC1062) | 3tfh | 45 | 44 | 0.99 | 3.18 |
| 5 | Oxidoreductase | dmgdHh | Rattus norvegicus | 4p9sA | 18 | 21 | 0.98 | 2.36 |
| 6 | AMT | dmdA | Pelagibacter ubique (HTCC1062) | 3tfhA | 43 | 44 | 0.99 | 5.76 |
| 7 | AMT | dmdA | Pelagibacter ubique (HTCC1062) | 3tfh | 43 | 44 | 0.98 | 3.52 |
| 8 | AMT | gcvTg | Bacillus subtilis | 1yx2A | 24 | 26 | 0.96 | 6.61 |
| 9 | AMT | dmdA | Pelagibacter ubique (HTCC1062) | 3tfhA | 44 | 44 | 1.00 | 7.68 |
| 10 | AMT | dmdA | Pelagibacter ubique (HTCC1062) | 3tfhA | 43 | 44 | 1.00 | 6.76 |

aIden1 is the percentage sequence identity of the templates in the threading aligned region with the query sequence

bIden2 is the percentage sequence identity of the whole template chains with query sequence 

cCov-Represents the coverage of the threading alignment and is equal to the number of aligned residues divided by the length of the query protein

dNorm. Z-score is the normalized Z-score of the threading alignments. Alignment with a normalized Z-score>1 means a good alignment and vice versa.

eAminomethyltransferase

fDmdA DMSP-dependent demethylase

gGlycine cleavage system T protein

hDimethylglycine dehydrogenase complexed with tetrahydrofolate 

Predicted model: C-sore=2; TM-score=0.99 dev= 0.04

| Rank | Class | Gene name | Organism | PDB ID | TM-scorea | RMSDb | IDENc | Covd |
| --- | --- | --- | --- | --- | --- | --- | --- | --- |
| 1 | AMT | dmdA | Pelagibacter ubique HTCC1062 | 3tfhA | 0.997 | 0.37 | 0.439 | 1.000 |
| 2 | Oxidoreductase | dmgdH | Rattus norvegicus | 4p9sA | 0.929 | 1.82 | 0.183 | 0.984 |
| 3 | Oxidoreductase | dmge | Arthrobacter globiformis | 1pj6A2 | 0.913 | 2.16 | 0.263 | 0.984 |
| 4 | Oxidoreductase | dmg | Arthrobacter globiformis | 1pj7A | 0.913 | 2.17 | 0.263 | 0.984 |
| 5 | AMT | gcvT | Pyrococcus horikoshii | 1v5vA | 0.904 | 1.95 | 0.239 | 0.967 |
| 6 | Oxidoreductase | soxAf | Stenotrophomonas maltophilia | 2gagA | 0.899 | 2.31 | 0.202 | 0.976 |
| 7 | Oxidoreductase | soxA | Corynebacterium sp. U-96 | 3ad7A | 0.898 | 2.28 | 0.202 | 0.976 |
| 8 | AMT | gcvT | Thermotoga maritima | 1worA | 0.896 | 1.82 | 0.249 | 0.951 |
| 9 | AMT | AMT(GCST) | Homo sapiens | 1wsrA | 0.890 | 2.09 | 0.244 | 0.959 |
| 10 | AMT | gcvT | Bartonella henselae | 3girA | 0.885 | 1.94 | 0.236 | 0.946 |

abIt is a standard for measuring structural similarity between two structures

cIt is the percentage sequence identity in the structurally aligned region 

dIt represents the coverage of the alignment by TM-align and is equal to the number of structurally aligned residues divided by length of the query protein

eDimethylglycine oxidase

fHeterotetrameric sarcosine oxidase alpha-subunit

Table 43. Up: Top ten analogs identified by LOMETS for threading alignments. Case of ABV94056.1. In the middle: Model predicted by I-TASSER. Down: Top ten identified structural analogs in PDB by TM-align

| Rank | Class | Gene name | Organism | PDB ID | Iden1 (%)a | Iden2 (%)b | Covc | Norm. Z-scored |
| --- | --- | --- | --- | --- | --- | --- | --- | --- |
| 1 | AMTe | dmdAf | Pelagibacter ubique (HTCC1062) | 3tfhA | 41 | 41 | 1.00 | 4.91 |
| 2 | AMT | dmdA | Pelagibacter ubique (HTCC1062) | 3tfhA | 41 | 41 | 1.00 | 5.45 |
| 3 | AMT | dmdA | Pelagibacter ubique (HTCC1062) | 3tfhA | 41 | 41 | 1.00 | 5.84 |
| 4 | AMT | dmdA | Pelagibacter ubique (HTCC1062) | 3tfh | 41 | 41 | 0.99 | 3.20 |
| 5 | Oxidoreductase | dmgdHh | Rattus norvegicus | 4p9sA | 18 | 21 | 0.98 | 2.35 |
| 6 | AMT | dmdA | Pelagibacter ubique (HTCC1062) | 3tfhA | 41 | 41 | 1.00 | 5.92 |
| 7 | AMT | dmdA | Pelagibacter ubique (HTCC1062) | 3tfh | 40 | 41 | 0.98 | 3.52 |
| 8 | AMT | gcvTg | Bacillus subtilis | 1yx2A | 25 | 25 | 0.96 | 6.40 |
| 9 | AMT | dmdA | Pelagibacter ubique (HTCC1062) | 3tfhA | 41 | 41 | 1.00 | 8.02 |
| 10 | AMT | dmdA | Pelagibacter ubique (HTCC1062) | 3tfhA | 41 | 41 | 1.00 | 6.90 |

aIden1 is the percentage sequence identity of the templates in the threading aligned region with the query sequence

bIden2 is the percentage sequence identity of the whole template chains with query sequence 

cCov-Represents the coverage of the threading alignment and is equal to the number of aligned residues divided by the length of the query protein

dNorm. Z-score is the normalized Z-score of the threading alignments. Alignment with a normalized Z-score>1 means a good alignment and vice versa.

eAminomethyltransferase

fDmdA DMSP-dependent demethylase

gGlycine cleavage system T protein

hDimethylglycine dehydrogenase complexed with tetrahydrofolate 

Predicted model: C-sore=2; TM-score=0.99 dev= 0.04

| Rank | Class | Gene name | Organism | PDB ID | TM-scorea | RMSDb | IDENc | Covd |
| --- | --- | --- | --- | --- | --- | --- | --- | --- |
| 1 | AMT | dmdA | Pelagibacter ubique HTCC1062 | 3tfhA | 0.998 | 0.35 | 0.408 | 1.000 |
| 2 | Oxidoreductase | dmgdH | Rattus norvegicus | 4p9sA | 0.927 | 1.87 | 0.180 | 0.984 |
| 3 | Oxidoreductase | dmge | Arthrobacter globiformis | 1pj6A2 | 0.910 | 2.25 | 0.276 | 0.984 |
| 4 | Oxidoreductase | dmg | Arthrobacter globiformis | 1pj7A | 0.910 | 2.25 | 0.276 | 0.984 |
| 5 | AMT | gcvT | Pyrococcus horikoshii | 1v5vA | 0.902 | 1.94 | 0.237 | 0.965 |
| 6 | Oxidoreductase | soxAf | Stenotrophomonas maltophilia | 2gagA | 0.897 | 2.40 | 0.201 | 0.976 |
| 7 | Oxidoreductase | soxA | Corynebacterium sp. U-96 | 3ad7A | 0.897 | 2.37 | 0.201 | 0.976 |
| 8 | AMT | gcvT | Thermotoga maritima | 1wooA | 0.895 | 1.75 | 0.253 | 0.946 |
| 9 | AMT | AMT(GCST) | Homo sapiens | 1wsrA | 0.888 | 2.09 | 0.261 | 0.957 |
| 10 | AMT | gcvT | Bartonella henselae | 3girA | 0.883 | 1.94 | 0.242 | 0.943 |

abIt is a standard for measuring structural similarity between two structures

cIt is the percentage sequence identity in the structurally aligned region 

dIt represents the coverage of the alignment by TM-align and is equal to the number of structurally aligned residues divided by length of the query protein

eDimethylglycine oxidase

fHeterotetrameric sarcosine oxidase alpha-subunit

Table 44. Up: Top ten analogs identified by LOMETS for threading alignments. Case of AAZ21068.1. In the middle: Model predicted by I-TASSER. Down: Top ten identified structural analogs in PDB by TM-align

| Rank | Class | Gene name | Organism | PDB ID | Iden1 (%)a | Iden2 (%)b | Covc | Norm. Z-scored |
| --- | --- | --- | --- | --- | --- | --- | --- | --- |
| 1 | AMTe | dmdAf | Pelagibacter ubique (HTCC1062) | 3tfhA | 100 | 100 | 1.00 | 4.99 |
| 2 | AMT | dmdA | Pelagibacter ubique (HTCC1062) | 3tfhA | 100 | 100 | 1.00 | 5.58 |
| 3 | AMT | dmdA | Pelagibacter ubique (HTCC1062) | 3tfhA | 100 | 100 | 1.00 | 5.83 |
| 4 | AMT | dmdA | Pelagibacter ubique (HTCC1062) | 3tfh | 100 | 100 | 1.00 | 3.31 |
| 5 | Oxidoreductase | dmgdHg | Rattus norvegicus | 4p9sA | 18 | 21 | 0.98 | 2.35 |
| 6 | AMT | dmdA | Pelagibacter ubique (HTCC1062) | 3tfhA | 100 | 100 | 1.00 | 6.20 |
| 7 | AMT | dmdA | Pelagibacter ubique (HTCC1062) | 3tfh | 0.94 | 100 | 0.99 | 3.57 |
| 8 | AMT | dmdA | Pelagibacter ubique (HTCC1062) | 3tfjA | 100 | 100 | 1.00 | 6.47 |
| 9 | AMT | dmdA | Pelagibacter ubique (HTCC1062) | 3tfhA | 100 | 100 | 1.00 | 8.10 |
| 10 | AMT | dmdA | Pelagibacter ubique (HTCC1062) | 3tfhA | 100 | 100 | 1.00 | 7.00 |

aIden1 is the percentage sequence identity of the templates in the threading aligned region with the query sequence

bIden2 is the percentage sequence identity of the whole template chains with query sequence 

cCov-Represents the coverage of the threading alignment and is equal to the number of aligned residues divided by the length of the query protein

dNorm. Z-score is the normalized Z-score of the threading alignments. Alignment with a normalized Z-score>1 means a good alignment and vice versa.

eAminomethyltransferase

fDmdA DMSP-dependent demethylase

gDimethylglycine dehydrogenase complexed with tetrahydrofolate 

Predicted model: C-sore=2; TM-score=0.99 dev= 0.04

| Rank | Class | Gene name | Organism | PDB ID | TM-scorea | RMSDb | IDENc | Covd |
| --- | --- | --- | --- | --- | --- | --- | --- | --- |
| 1 | AMT | dmdA | Pelagibacter ubique HTCC1062 | 3tfhA | 0.997 | 0.41 | 1.000 | 1.000 |
| 2 | Oxidoreductase | dmgdH | Rattus norvegicus | 4p9sA | 0.929 | 1.83 | 0.193 | 0.984 |
| 3 | Oxidoreductase | dmge | Arthrobacter globiformis | 1pj6A | 0.912 | 2.20 | 0.220 | 0.984 |
| 4 | AMT | gcvT | Pyrococcus horikoshii | 1v5vA | 0.901 | 1.93 | 0.231 | 0.962 |
| 5 | Oxidoreductase | soxAf | Corynebacterium sp. U-96 | 3ad7A | 0.896 | 2.34 | 0.206 | 0.973 |
| 6 | Oxidoreductase | soxA | Stenotrophomonas maltophilia | 2gagA | 0.896 | 2.28 | 0.198 | 0.970 |
| 7 | AMT | gcvT | Thermotoga maritima | 1wooA | 0.894 | 1.72 | 0.236 | 0.943 |
| 8 | AMT | AMT(GCST) | Homo sapiens | 1wsrA | 0.887 | 2.07 | 0.185 | 0.954 |
| 9 | AMT | gcvT | Bartonella henselae | 3girA | 0.882 | 1.91 | 0.196 | 0.940 |
| 10 | AMT | gcvT | Bacillus subtilis | 1yx2B | 0.875 | 2.15 | 0.246 | 0.946 |

abIt is a standard for measuring structural similarity between two structures

cIt is the percentage sequence identity in the structurally aligned region 

dIt represents the coverage of the alignment by TM-align and is equal to the number of structurally aligned residues divided by length of the query protein

eDimethylglycine oxidase

fHeterotetrameric sarcosine oxidase alpha-subunit

Table 45. Up: Top ten analogs identified by LOMETS for threading alignments. Case of AFS46782.1. In the middle: Model predicted by I-TASSER. Down: Top ten identified structural analogs in PDB by TM-align

| Rank | Class | Gene name | Organism | PDB ID | Iden1 (%)a | Iden2 (%)b | Covc | Norm. Z-scored |
| --- | --- | --- | --- | --- | --- | --- | --- | --- |
| 1 | AMTe | dmdAf | Pelagibacter ubique (HTCC1062) | 3tfhA | 76 | 76 | 1.00 | 4.94 |
| 2 | AMT | dmdA | Pelagibacter ubique (HTCC1062) | 3tfhA | 76 | 76 | 1.00 | 5.45 |
| 3 | AMT | dmdA | Pelagibacter ubique (HTCC1062) | 3tfhA | 74 | 76 | 1.00 | 5.62 |
| 4 | AMT | dmdA | Pelagibacter ubique (HTCC1062) | 3tfh | 75 | 76 | 0.99 | 3.23 |
| 5 | Oxidoreductase | dmgdHh | Rattus norvegicus | 4p9sA | 17 | 21 | 0.98 | 2.36 |
| 6 | AMT | dmdA | Pelagibacter ubique (HTCC1062) | 3tfhA | 76 | 76 | 1.00 | 6.08 |
| 7 | AMT | dmdA | Pelagibacter ubique (HTCC1062) | 3tfh | 71 | 76 | 0.99 | 3.57 |
| 8 | AMT | gcvTg | Bacillus subtilis | 1yx2A | 26 | 27 | 0.96 | 6.77 |
| 9 | AMT | dmdA | Pelagibacter ubique (HTCC1062) | 3tfhA | 76 | 76 | 1.00 | 7.90 |
| 10 | AMT | dmdA | Pelagibacter ubique (HTCC1062) | 3tfhA | 74 | 76 | 1.00 | 6.85 |

aIden1 is the percentage sequence identity of the templates in the threading aligned region with the query sequence

bIden2 is the percentage sequence identity of the whole template chains with query sequence 

cCov-Represents the coverage of the threading alignment and is equal to the number of aligned residues divided by the length of the query protein

dNorm. Z-score is the normalized Z-score of the threading alignments. Alignment with a normalized Z-score>1 means a good alignment and vice versa.

eAminomethyltransferase

fDmdA DMSP-dependent demethylase

gGlycine cleavage system T protein

hDimethylglycine dehydrogenase complexed with tetrahydrofolate 

Predicted model: C-sore=1.95; TM-score=0.99 dev= 0.04

| Rank | Class | Gene name | Organism | PDB ID | TM-scorea | RMSDb | IDENc | Covd |
| --- | --- | --- | --- | --- | --- | --- | --- | --- |
| 1 | AMT | dmdA | Pelagibacter ubique HTCC1062 | 3tfhA | 0.997 | 0.41 | 0.753 | 1.000 |
| 2 | Oxidoreductase | dmgdH | Rattus norvegicus | 4p9sA | 0.931 | 1.84 | 0.182 | 0.986 |
| 3 | Oxidoreductase | dmge | Arthrobacter globiformis | 1pj6A | 0.913 | 2.22 | 0.212 | 0.986 |
| 4 | AMT | gcvT | Pyrococcus horikoshii | 1v5vA | 0.900 | 1.99 | 0.237 | 0.965 |
| 5 | Oxidoreductase | soxAf | Stenotrophomonas maltophilia | 2gagA | 0.895 | 2.30 | 0.201 | 0.970 |
| 6 | Oxidoreductase | soxA | Corynebacterium sp. U-96 | 3ad7A | 0.895 | 2.36 | 0.218 | 0.973 |
| 7 | AMT | gcvT | Thermotoga maritima | 1wooA | 0.893 | 1.77 | 0.253 | 0.946 |
| 8 | AMT | AMT(GCST) | Homo sapiens | 1wsrA | 0.885 | 2.10 | 0.199 | 0.954 |
| 9 | AMT | gcvT | Bartonella henselae | 3girA | 0.880 | 1.99 | 0.207 | 0.943 |
| 10 | AMT | gcvT | Bacillus subtilis | 1yx2B | 0.876 | 2.10 | 0.259 | 0.946 |

abIt is a standard for measuring structural similarity between two structures

cIt is the percentage sequence identity in the structurally aligned region 

dIt represents the coverage of the alignment by TM-align and is equal to the number of structurally aligned residues divided by length of the query protein

eDimethylglycine oxidase

fHeterotetrameric sarcosine oxidase alpha-subunit

Table 46. Up: Top ten analogs identified by LOMETS for threading alignments. Case of AFS48343.1. In the middle: Model predicted by I-TASSER. Down: Top ten identified structural analogs in PDB by TM-align

| Rank | Class | Gene name | Organism | PDB ID | Iden1 (%)a | Iden2 (%)b | Covc | Norm. Z-scored |
| --- | --- | --- | --- | --- | --- | --- | --- | --- |
| 1 | AMTe | dmdAf | Pelagibacter ubique (HTCC1062) | 3tfhA | 56 | 55 | 1.00 | 4.95 |
| 2 | AMT | dmdA | Pelagibacter ubique (HTCC1062) | 3tfhA | 55 | 55 | 1.00 | 5.39 |
| 3 | AMT | dmdA | Pelagibacter ubique (HTCC1062) | 3tfhA | 55 | 55 | 1.00 | 5.76 |
| 4 | AMT | dmdA | Pelagibacter ubique (HTCC1062) | 3tfh | 55 | 55 | 0.99 | 3.19 |
| 5 | Oxidoreductase | dmgdHh | Rattus norvegicus | 4p9sA | 18 | 23 | 0.98 | 2.36 |
| 6 | AMT | dmdA | Pelagibacter ubique (HTCC1062) | 3tfhA | 55 | 55 | 0.99 | 5.96 |
| 7 | AMT | dmdA | Pelagibacter ubique (HTCC1062) | 3tfh | 54 | 55 | 0.98 | 3.55 |
| 8 | AMT | gcvTg | Bacillus subtilis | 1yx2A | 23 | 25 | 0.96 | 6.47 |
| 9 | AMT | dmdA | Pelagibacter ubique (HTCC1062) | 3tfhA | 56 | 55 | 1.00 | 7.94 |
| 10 | AMT | dmdA | Pelagibacter ubique (HTCC1062) | 3tfhA | 56 | 55 | 1.00 | 6.73 |

aIden1 is the percentage sequence identity of the templates in the threading aligned region with the query sequence

bIden2 is the percentage sequence identity of the whole template chains with query sequence 

cCov-Represents the coverage of the threading alignment and is equal to the number of aligned residues divided by the length of the query protein

dNorm. Z-score is the normalized Z-score of the threading alignments. Alignment with a normalized Z-score>1 means a good alignment and vice versa.

eAminomethyltransferase

fDmdA DMSP-dependent demethylase

gGlycine cleavage system T protein

hDimethylglycine dehydrogenase complexed with tetrahydrofolate 

Predicted model: C-sore=2; TM-score=0.99 dev= 0.04

| Rank | Class | Gene name | Organism | PDB ID | TM-scorea | RMSDb | IDENc | Covd |
| --- | --- | --- | --- | --- | --- | --- | --- | --- |
| 1 | AMT | dmdA | Pelagibacter ubique HTCC1062 | 3tfhA | 0.995 | 0.36 | 0.553 | 0.997 |
| 2 | Oxidoreductase | dmgdH | Rattus norvegicus | 4p9sA | 0.930 | 1.84 | 0.193 | 0.986 |
| 3 | Oxidoreductase | dmge | Arthrobacter globiformis | 1pj6A | 0.913 | 2.22 | 0.218 | 0.986 |
| 4 | Oxidoreductase | dmg | Arthrobacter globiformis | 1pj6A2 | 0.913 | 2.22 | 0.218 | 0.986 |
| 5 | AMT | gcvT | Pyrococcus horikoshii | 1v5vA | 0.903 | 1.99 | 0.247 | 0.967 |
| 6 | Oxidoreductase | soxAf | Stenotrophomonas maltophilia | 2gagA | 0.898 | 2.30 | 0.196 | 0.973 |
| 7 | Oxidoreductase | soxA | Corynebacterium sp. U-96 | 3ad7A | 0.897 | 2.27 | 0.201 | 0.973 |
| 8 | AMT | gcvT | Thermotoga maritima | 1wooA | 0.896 | 1.77 | 0.244 | 0.948 |
| 9 | AMT | AMT(GCST) | Homo sapiens | 1wsrA | 0.889 | 2.09 | 0.233 | 0.957 |
| 10 | AMT | gcvT | Bartonella henselae | 3girA | 0.883 | 1.98 | 0.204 | 0.946 |

abIt is a standard for measuring structural similarity between two structures

cIt is the percentage sequence identity in the structurally aligned region 

dIt represents the coverage of the alignment by TM-align and is equal to the number of structurally aligned residues divided by length of the query protein

eDimethylglycine oxidase

fHeterotetrameric sarcosine oxidase alpha-subunit

Table 47. Up: Top ten analogs identified by LOMETS for threading alignments. Case of ADE38317.1. In the middle: Model predicted by I-TASSER. Down: Top ten identified structural analogs in PDB by TM-align

| Rank | Class | Gene name | Organism | PDB ID | Iden1 (%)a | Iden2 (%)b | Covc | Norm. Z-scored |
| --- | --- | --- | --- | --- | --- | --- | --- | --- |
| 1 | AMTe | dmdAf | Pelagibacter ubique (HTCC1062) | 3tfhA | 46 | 46 | 0.99 | 4.75 |
| 2 | AMT | dmdA | Pelagibacter ubique (HTCC1062) | 3tfhA | 46 | 46 | 0.99 | 5.36 |
| 3 | AMT | dmdA | Pelagibacter ubique (HTCC1062) | 3tfhA | 46 | 46 | 0.99 | 5.59 |
| 4 | AMT | dmdA | Pelagibacter ubique (HTCC1062) | 3tfh | 46 | 46 | 0.99 | 3.23 |
| 5 | Oxidoreductase | dmgdHh | Rattus norvegicus | 4p9sA | 19 | 22 | 0.97 | 2.35 |
| 6 | AMT | dmdA | Pelagibacter ubique (HTCC1062) | 3tfhA | 46 | 46 | 0.99 | 5.72 |
| 7 | AMT | dmdA | Pelagibacter ubique (HTCC1062) | 3tfh | 46 | 46 | 0.98 | 3.58 |
| 8 | AMT | gcvTg | Thermotoga maritima | 1wosA | 23 | 24 | 0.94 | 5.99 |
| 9 | AMT | dmdA | Pelagibacter ubique (HTCC1062) | 3tfhA | 46 | 46 | 0.99 | 7.99 |
| 10 | AMT | dmdA | Pelagibacter ubique (HTCC1062) | 3tfhA | 46 | 46 | 0.99 | 6.75 |

aIden1 is the percentage sequence identity of the templates in the threading aligned region with the query sequence

bIden2 is the percentage sequence identity of the whole template chains with query sequence 

cCov-Represents the coverage of the threading alignment and is equal to the number of aligned residues divided by the length of the query protein

dNorm. Z-score is the normalized Z-score of the threading alignments. Alignment with a normalized Z-score>1 means a good alignment and vice versa.

eAminomethyltransferase

fDmdA DMSP-dependent demethylase

gGlycine cleavage system T protein

hDimethylglycine dehydrogenase complexed with tetrahydrofolate 

Predicted model: C-sore=1.96; TM-score=0.99 dev= 0.04

| Rank | Class | Gene name | Organism | PDB ID | TM-scorea | RMSDb | IDENc | Covd |
| --- | --- | --- | --- | --- | --- | --- | --- | --- |
| 1 | AMT | dmdA | Pelagibacter ubique HTCC1062 | 3tfhA | 0.988 | 0.47 | 0.462 | 0.992 |
| 2 | Oxidoreductase | dmgdH | Rattus norvegicus | 4p9sA | 0.924 | 1.83 | 0.196 | 0.978 |
| 3 | Oxidoreductase | dmge | Arthrobacter globiformis | 1pj6A | 0.907 | 2.21 | 0.231 | 0.978 |
| 4 | AMT | gcvT | Pyrococcus horikoshii | 1v5vA | 0.895 | 1.92 | 0.254 | 0.954 |
| 5 | Oxidoreductase | soxAf | Corynebacterium sp. U-96 | 3ad7A | 0.894 | 2.24 | 0.214 | 0.968 |
| 6 | Oxidoreductase | soxA | Stenotrophomonas maltophilia | 2gagA | 0.894 | 2.24 | 0.211 | 0.968 |
| 7 | AMT | gcvT | Thermotoga maritima | 1wooA | 0.888 | 1.74 | 0.227 | 0.938 |
| 8 | AMT | AMT(GCST) | Homo sapiens | 1wsrA | 0.882 | 2.08 | 0.227 | 0.949 |
| 9 | AMT | gcvT | Bartonella henselae | 3girA | 0.878 | 1.92 | 0.248 | 0.935 |
| 10 | AMT | gcvT | Bacillus subtilis | 1yx2B | 0.868 | 2.09 | 0.265 | 0.935 |

abIt is a standard for measuring structural similarity between two structures

cIt is the percentage sequence identity in the structurally aligned region 

dIt represents the coverage of the alignment by TM-align and is equal to the number of structurally aligned residues divided by length of the query protein

eDimethylglycine oxidase

fHeterotetrameric sarcosine oxidase alpha-subunit

Table 48. Up: Top ten analogs identified by LOMETS for threading alignments. Case of AAV95190.1. In the middle: Model predicted by I-TASSER. Down: Top ten identified structural analogs in PDB by TM-align

| Rank | Class | Gene name | Organism | PDB ID | Iden1 (%)a | Iden2 (%)b | Covc | Norm. Z-scored |
| --- | --- | --- | --- | --- | --- | --- | --- | --- |
| 1 | AMTe | dmdAf | Pelagibacter ubique (HTCC1062) | 3tfhA | 40 | 41 | 0.99 | 4.65 |
| 2 | AMT | gcvTg | Pyrococcus horikoshii | 1v5vA | 22 | 26 | 0.98 | 5.21 |
| 3 | AMT | gcvT | Thermotoga maritima | 1worA | 22 | 25 | 0.96 | 5.69 |
| 4 | AMT | dmdA | Pelagibacter ubique (HTCC1062) | 3tfh | 41 | 41 | 1.00 | 3.20 |
| 5 | Oxidoreductase | dmgdHh | Rattus norvegicus | 4p9sA | 18 | 21 | 0.99 | 2.36 |
| 6 | AMT | dmdA | Pelagibacter ubique (HTCC1062) | 3tfhA | 39 | 41 | 0.99 | 5.60 |
| 7 | AMT | dmgdH | Rattus norvegicus | 4p9sA | 18 | 21 | 0.98 | 3.52 |
| 8 | AMT | gcvT | Bacillus subtilis | 1yx2A | 23 | 24 | 0.96 | 6.47 |
| 9 | AMT | dmdA | Pelagibacter ubique (HTCC1062) | 3tfhA | 41 | 41 | 1.00 | 7.46 |
| 10 | AMT | dmdA | Pelagibacter ubique (HTCC1062) | 3tfhA | 41 | 41 | 1.00 | 6.69 |

aIden1 is the percentage sequence identity of the templates in the threading aligned region with the query sequence

bIden2 is the percentage sequence identity of the whole template chains with query sequence 

cCov-Represents the coverage of the threading alignment and is equal to the number of aligned residues divided by the length of the query protein

dNorm. Z-score is the normalized Z-score of the threading alignments. Alignment with a normalized Z-score>1 means a good alignment and vice versa.

eAminomethyltransferase

fDmdA DMSP-dependent demethylase

gGlycine cleavage system T protein

hDimethylglycine dehydrogenase complexed with tetrahydrofolate 

Predicted model: C-sore=1.45; TM-score=0.92 dev= 0.06

| Rank | Class | Gene name | Organism | PDB ID | TM-scorea | RMSDb | IDENc | Covd |
| --- | --- | --- | --- | --- | --- | --- | --- | --- |
| 1 | AMT | dmdA | Pelagibacter ubique HTCC1062 | 3tfhA | 0.974 | 1.19 | 0.407 | 1.000 |
| 2 | Oxidoreductase | dmgdH | Rattus norvegicus | 4p9sA | 0.944 | 1.56 | 0.178 | 0.986 |
| 3 | AMT | gcvT | Pyrococcus horikoshii | 1v5vA | 0.935 | 1.53 | 0.214 | 0.975 |
| 4 | AMT | gcvT | Thermotoga maritima | 1worA | 0.930 | 1.33 | 0.234 | 0.962 |
| 5 | Oxidoreductase | dmge | Arthrobacter globiformis | 1pj6A2 | 0.928 | 2.02 | 0.239 | 0.989 |
| 6 | Oxidoreductase | dmg | Arthrobacter globiformis | 1pj7A | 0.928 | 2.02 | 0.239 | 0.989 |
| 7 | Oxidoreductase | soxAf | Stenotrophomonas maltophilia | 2gagA | 0.922 | 1.99 | 0.193 | 0.984 |
| 8 | Oxidoreductase | soxA | Corynebacterium sp. U-96 | 1x31A | 0.921 | 2.01 | 0.182 | 0.984 |
| 9 | AMT | AMT(GCST) | Homo sapiens | 1wsrA | 0.910 | 1.66 | 0.256 | 0.956 |
| 10 | AMT | gcvT | Bartonella henselae | 3girA | 0.903 | 1.71 | 0.228 | 0.951 |

abIt is a standard for measuring structural similarity between two structures

cIt is the percentage sequence identity in the structurally aligned region 

dIt represents the coverage of the alignment by TM-align and is equal to the number of structurally aligned residues divided by length of the query protein

eDimethylglycine oxidase

fHeterotetrameric sarcosine oxidase alpha-subunit

Table 49. Up: Top ten analogs identified by LOMETS for threading alignments. Case of “ancestral dmda and no-dmda sequence” inferred by FastML. In the middle: Model predicted by I-TASSER. Down: Top ten identified structural analogs in PDB by TM-align

| Rank | Class | Gene name | Organism | PDB ID | Iden1 (%)a | Iden2 (%)b | Covc | Norm. Z-scored |
| --- | --- | --- | --- | --- | --- | --- | --- | --- |
| 1 | AMTe | gcvTg | Thermotoga maritima | 1wopA | 29 | 34 | 1.00 | 3.97 |
| 2 | AMT | AMT(GCST) | Homo sapiens | 1wsrA | 25 | 30 | 1.00 | 3.91 |
| 3 | AMT | gcvT | Thermotoga maritima | 1worA | 30 | 34 | 0.99 | 4.77 |
| 4 | AMT | dmdAf | Pelagibacter ubique HTCC1062 | 3tfh | 31 | 32 | 1.00 | 3.19 |
| 5 | Oxidoreductase | dmgdHh | Rattus norvegicus | 4p9sA | 16 | 23 | 0.99 | 2.30 |
| 6 | AMT | gcvT | Bartonella henselae | 3girA | 23 | 29 | 1.00 | 4.49 |
| 7 | Oxidoreductase | dmgdHh | Rattus norvegicus | 4p9sA | 19 | 23 | 0.99 | 3.37 |
| 8 | AMT | gcvT | Bacillus subtilis | 1yx2A | 27 | 31 | 0.99 | 4.64 |
| 9 | AMT | dmdA | Pelagibacter ubique HTCC1062 | 3tfhA | 28 | 32 | 0.99 | 6.12 |
| 10 | AMT | gcvT | Bartonella henselae | 3girA | 24 | 29 | 0.99 | 5.62 |

aIden1 is the percentage sequence identity of the templates in the threading aligned region with the query sequence

bIden2 is the percentage sequence identity of the whole template chains with query sequence 

cCov-Represents the coverage of the threading alignment and is equal to the number of aligned residues divided by the length of the query protein

dNorm. Z-score is the normalized Z-score of the threading alignments. Alignment with a normalized Z-score>1 means a good alignment and vice versa.

eAminomethyltransferase

fDmdA DMSP-dependent demethylase

gGlycine cleavage system T protein

hDimethylglycine dehydrogenase complexed with tetrahydrofolate 

Predicted model: C-sore=1.25; TM-score=0.89 dev= 0.07

| Rank | Class | Gene name | Organism | PDB ID | TM-scorea | RMSDb | IDENc | Covd |
| --- | --- | --- | --- | --- | --- | --- | --- | --- |
| 1 | AMT | gcvT | Thermotoga maritima | 1wooA | 0.960 | 1.39 | 0.278 | 0.997 |
| 2 | Oxidoreductase | dmgdH | Rattus norvegicus | 4p9sA | 0.951 | 1.48 | 0.155 | 0.993 |
| 3 | AMT | AMT(GCST) | Homo sapiens | 1wsrA | 0.948 | 1.57 | 0.236 | 0.997 |
| 4 | AMT | gcvT | Pyrococcus horikoshii | 1v5vA | 0.947 | 1.57 | 0.254 | 0.997 |
| 5 | Oxidoreductase | soxA | Corynebacterium sp U-96 | 1vrqA2 | 0.942 | 1.72 | 0.232 | 1.000 |
| 6 | Oxidoreductase | soxA | Corynebacterium sp. U-96 | 1x31A | 0.941 | 1.73 | 0.232 | 1.000 |
| 7 | Oxidoreductase | soxAf | Stenotrophomonas maltophilia | 2gagA | 0.941 | 1.73 | 0.242 | 1.000 |
| 8 | Oxidoreductase | dmge | Arthrobacter globiformis | 1pj7A2 | 0.928 | 1.89 | 0.225 | 0.997 |
| 9 | Oxidoreductase | dmg | Arthrobacter globiformis | 1pj6A | 0.928 | 1.89 | 0.225 | 0.997 |
| 10 | AMT | gcvT | Bartonella henselae | 3girA | 0.927 | 1.87 | 0.234 | 0.990 |

abIt is a standard for measuring structural similarity between two structures

cIt is the percentage sequence identity in the structurally aligned region 

dIt represents the coverage of the alignment by TM-align and is equal to the number of structurally aligned residues divided by length of the query protein

eDimethylglycine oxidase

fHeterotetrameric sarcosine oxidase alpha-subunit

Table 50. Up: Top ten analogs identified by LOMETS for threading alignments. Case of “ancestral dmda sequence” inferred by FastML. In the middle: Model predicted by I-TASSER. Down: Top ten identified structural analogs in PDB by TM-align

| Rank | Class | Gene name | Organism | PDB ID | Iden1 (%)a | Iden2 (%)b | Covc | Norm. Z-scored |
| --- | --- | --- | --- | --- | --- | --- | --- | --- |
| 1 | AMTe | dmdAf | Pelagibacter ubique HTCC1062 | 3tfhA | 83 | 85 | 1.00 | 4.93 |
| 2 | AMT | dmdA | Pelagibacter ubique HTCC1062 | 3tfhA | 83 | 85 | 1.00 | 5.48 |
| 3 | AMT | dmdA | Pelagibacter ubique HTCC1062 | 3tfhA | 83 | 82 | 1.00 | 5.67 |
| 4 | AMT | dmdA | Pelagibacter ubique HTCC1062 | 3tfh | 83 | 85 | 1.00 | 3.19 |
| 5 | Oxidoreductase | dmgdHh | Rattus norvegicus | 4p9sA | 17 | 20 | 0.98 | 2.33 |
| 6 | AMT | dmdA | Pelagibacter ubique HTCC1062 | 3tfhA | 83 | 85 | 1.00 | 6.03 |
| 7 | Oxidoreductase | dmgdH | Rattus norvegicus | 4p9s | 17 | 20 | 0.98 | 3.50 |
| 8 | AMT | gcvTg | Bacillus subtilis | 1yx2A | 26 | 27 | 0.96 | 6.83 |
| 9 | AMT | dmdA | Pelagibacter ubique HTCC1062 | 3tfhA | 83 | 85 | 1.00 | 8.06 |
| 10 | AMT | dmdA | BPelagibacter ubique HTCC1062 | 3tfhA | 83 | 85 | 1.00 | 6.88 |

aIden1 is the percentage sequence identity of the templates in the threading aligned region with the query sequence

bIden2 is the percentage sequence identity of the whole template chains with query sequence 

cCov-Represents the coverage of the threading alignment and is equal to the number of aligned residues divided by the length of the query protein

dNorm. Z-score is the normalized Z-score of the threading alignments. Alignment with a normalized Z-score>1 means a good alignment and vice versa.

eAminomethyltransferase

fDmdA DMSP-dependent demethylase

gGlycine cleavage system T protein

hDimethylglycine dehydrogenase complexed with tetrahydrofolate 

Predicted model: C-sore=2; TM-score=0.99 dev= 0.04

| Rank | Class | Gene name | Organism | PDB ID | TM-scorea | RMSDb | IDENc | Covd |
| --- | --- | --- | --- | --- | --- | --- | --- | --- |
| 1 | AMTe | dmdA | Pelagibacter ubique HTCC1062 | 3tfhA | 0.997 | 0.42 | 0.825 | 1.000 |
| 2 | Oxidoreductase | dmgdH | Rattus norvegicus | 4p9sA | 0.930 | 1.84 | 0.181 | 0.986 |
| 3 | Oxidoreductase | dmge | Arthrobacter globiformis | 1pj6A2 | 0.914 | 2.19 | 0.228 | 0.986 |
| 4 | Oxidoreductase | dmg | Arthrobacter globiformis | 1pj7A | 0.914 | 2.19 | 0.228 | 0.986 |
| 5 | AMTe | gcvT | Pyrococcus horikoshii | 1v5vA | 0.905 | 2.03 | 0.239 | 0.973 |
| 6 | Oxidoreductase | soxAf | Corynebacterium sp. U-96 | 3ad7A | 0.899 | 2.31 | 0.216 | 0.978 |
| 7 | Oxidoreductase | soxA | Stenotrophomonas maltophilia | 2gagA | 0.899 | 2.34 | 0.194 | 0.978 |
| 8 | AMT | gcvT | Thermotoga maritima | 1wooA | 0.897 | 1.79 | 0.242 | 0.951 |
| 9 | AMT | AMT(GCST) | Homo sapiens | 1wsrA | 0.889 | 2.16 | 0.208 | 0.962 |
| 10 | AMT | gcvT | Bartonella henselae | 3girA | 0.885 | 1.98 | 0.211 | 0.948 |

abIt is a standard for measuring structural similarity between two structures

cIt is the percentage sequence identity in the structurally aligned region 

dIt represents the coverage of the alignment by TM-align and is equal to the number of structurally aligned residues divided by length of the query protein

eDimethylglycine oxidase

fHeterotetrameric sarcosine oxidase alpha-subunit

Table 51. Up: Top ten analogs identified by LOMETS for threading alignments. Case of “ancestral no-dmda sequence” inferred by FastML. In the middle: Model predicted by I-TASSER. Down: Top ten identified structural analogs in PDB by TM-align

| Rank | Class | Gene name | Organism | PDB ID | Iden1 (%)a | Iden2 (%)b | Covc | Norm. Z-scored |
| --- | --- | --- | --- | --- | --- | --- | --- | --- |
| 1 | AMTe | gcvTg | Thermotoga maritima | 1wopA | 29 | 31 | 0.91 | 3.93 |
| 2 | AMT | gcvT | Pyrococcus horikoshii | 1v5vA | 26 | 29 | 0.94 | 4.52 |
| 3 | AMT | gcvT | Thermotoga maritima | 1worA | 29 | 31 | 0.91 | 5.02 |
| 4 | Oxidoreductase | dmg | Arthrobacter globiformis | 1pj6 | 23 | 26 | 0.96 | 3.21 |
| 5 | Oxidoreductase | dmgdHh | Rattus norvegicus | 4p9sA | 16 | 24 | 0.96 | 2.38 |
| 6 | AMT | gcvT | Thermotoga maritima | 1wopA | 29 | 31 | 0.91 | 4.81 |
| 7 | Oxidoreductase | dmgi | Arthrobacter globiformis | 1pj6 | 22 | 26 | 0.97 | 3.57 |
| 8 | AMT | gcvT | Bacillus subtilis | 1yx2A | 26 | 26 | 0.89 | 5.31 |
| 9 | AMT | dmdAf | Pelagibacter ubique HTCC1062 | 3tfhA | 26 | 29 | 0.93 | 6.47 |
| 10 | Oxidoreductase | dmg | Arthrobacter globiformis | 1pj5A | 22 | 26 | 0.97 | 5.80 |

aIden1 is the percentage sequence identity of the templates in the threading aligned region with the query sequence

bIden2 is the percentage sequence identity of the whole template chains with query sequence 

cCov-Represents the coverage of the threading alignment and is equal to the number of aligned residues divided by the length of the query protein

dNorm. Z-score is the normalized Z-score of the threading alignments. Alignment with a normalized Z-score>1 means a good alignment and vice versa.

eAminomethyltransferase

fDmdA DMSP-dependent demethylase

gGlycine cleavage system T protein

hDimethylglycine dehydrogenase complexed with tetrahydrofolate 

iDimethylglycine oxidase

Predicted model: C-sore=0.76; TM-score=0.82 dev= 0.09

| Rank | Class | Gene name | Organism | PDB ID | TM-scorea | RMSDb | IDENc | Covd |
| --- | --- | --- | --- | --- | --- | --- | --- | --- |
| 1 | Oxidoreductase | dmgdH | Rattus norvegicus | 4p9sA | 0.940 | 1.63 | 0.158 | 0.983 |
| 2 | Oxidoreductase | dmg | Arthrobacter globiformis | 1pj7A | 0.914 | 2.23 | 0.225 | 0.986 |
| 3 | AMTe | gcvT | Pyrococcus horikoshii | 1v5vA | 0.892 | 1.68 | 0.250 | 0.933 |
| 4 | AMTe | gcvT | Thermotoga maritima | 1worA | 0.892 | 1.14 | 0.292 | 0.914 |
| 5 | AMTe | AMT(GCST) | Homo sapiens | 1wsvB | 0.882 | 1.59 | 0.217 | 0.922 |
| 6 | Oxidoreductase | soxAe | Stenotrophomonas maltophilia | 2gagA | 0.876 | 2.36 | 0.231 | 0.950 |
| 7 | Oxidoreductase | soxA | Corynebacterium_sp_U-96 | 1vrqA | 0.874 | 2.25 | 0.212 | 0.944 |
| 8 | AMT | gcvT | Bartonella henselae | 3girA | 0.864 | 1.80 | 0.207 | 0.911 |
| 9 | AMT | dmdA | Pelagibacter ubique HTCC1062 | 3tfiA | 0.859 | 2.18 | 0.254 | 0.931 |
| 10 | AMT | gcvT | Escherichia coli | 3a8iA | 0.859 | 1.82 | 0.208 | 0.903 |

abIt is a standard for measuring structural similarity between two structures

cIt is the percentage sequence identity in the structurally aligned region 

dIt represents the coverage of the alignment by TM-align and is equal to the number of structurally aligned residues divided by length of the query protein

eHeterotetrameric sarcosine oxidase alpha-subunit
